# High-Resolution Spatial Transcriptomics Reveals Pathological Microregion-Specific Luminal Subset Plasticity and Core Signaling Networks Underlying Prostate Cancer Malignancy

**DOI:** 10.3390/biomedicines14081710

**Published:** 2026-07-30

**Authors:** Wenzhe Hao, Zhigang Wang, Yandong Zhang, Zheng Qu, Dongting Chen, Gang Song

**Affiliations:** 1 Department of Urology, National Cancer Center/National Clinical Research Center for Cancer/Cancer Hospital, Chinese Academy of Medical Sciences and Peking Union Medical College, Beijing 100021, China; wolfcub2001@163.com; 2 Department of Biomedical Engineering, Institute of Basic Medical Sciences and School of Basic Medicine, Chinese Academy of Medical Sciences and Peking Union Medical College, Beijing 100005, China; wangzg@pumc.edu.cn; 3 Department of Pathology, Institute of Basic Medical Sciences and School of Basic Medicine, Chinese Academy of Medical Sciences and Peking Union Medical College, Beijing 100005, China; ydzhang@aliyun.com; 4 Department of Pharmacology, Institute of Materia Medica, Chinese Academy of Medical Sciences and Peking Union Medical College, Beijing 100050, China; quzheng@imm.ac.cn (Z.Q.); chendt@imm.ac.cn (D.C.)

**Keywords:** prostate cancer, luminal subsets, tumor microenvironment, cell communication, single-cell spatial transcriptomics

## Abstract

**Background:** Prostate cancer (PCa) exhibits strong multidimensional heterogeneity. The dynamic evolution of Luminal subsets and their crosstalk with the tumor microenvironment (TME) during malignant progression remain poorly understood. Conventional single-cell transcriptomics lacks spatial context, while low-resolution spatial technologies cannot resolve single-cell and subcellular interactions. This study aimed to map a high-resolution spatial transcriptomic atlas to characterize Luminal subset heterogeneity and stromal crosstalk across progressive PCa lesions. **Methods:** We used high-resolution CosMx spatial molecular imaging (SMI) to profile 6 treatment-naive PCa patients and constructed a single-cell spatial transcriptomic atlas covering 385 fields of view and 154,168 high-quality cells. Unsupervised clustering, functional enrichment, cell–cell communication analysis, and flow cytometry were performed. **Results:** We identified 28 cell clusters, including 8 Luminal subsets (0–7) and 9 Fibroblast subsets (0–8). Pathological microregion-specific spatial distribution was observed: Luminal 0–2 dominated in BPH/PIN, while Luminal 3–7 were enriched in poorly differentiated PCa and vacuolated adenocarcinoma. These subsets were functionally linked to androgen resistance, EMT, and immune regulation. COLLAGEN, FN1, and LAMININ were identified as key pathways mediating pathological-specific tumor–stromal crosstalk. Flow cytometry verified T-cell exhaustion and immunosuppression in the TME. **Conclusions:** This study delineated pathological microregion-specific Luminal subset plasticity and dynamic epithelial–stromal communication across the full spectrum of PCa lesions. The uncovered molecular signatures offered mechanistic clues linking EMT, hormone resistance, and local immune status during lesion evolution. Our spatial atlas provided preliminary molecular evidence to support pathological stratification and targeted precision therapeutic strategies for PCa.

## 1. Introduction

Cancer is a very lethal disease and a big challenge to the whole world. Mounting evidence indicates that aberrant expression of oncogenes and tumor suppressors drives the initiation and progression of carcinogenesis. A number of exogenous and endogenous risk factors including chemical carcinogens, ionizing radiation, and pathogenic microorganisms, contribute to tumor development across tissue types [1,2,3]. The current data for Prostate cancer (PCa) show that PCa is the second most common cancer in males, with 1,466,680 new cases of PCa and 396,792 deaths in 2022. The data have shown that approximately 5 million new cases of PCa are diagnosed yearly in different parts of the world [4,5]. Its intricate pathological heterogeneity, manifesting as multifocal lesions spanning benign prostatic hyperplasia (BPH), low-grade/high-grade prostatic intraepithelial neoplasia (LGPIN/HGPIN), well-differentiated to poorly differentiated PCa, and prostatic adenocarcinoma with vacuolization, poses a formidable barrier to precision diagnosis and treatment. Clinically, PCa heterogeneity encompasses genomic, transcriptomic, and phenotypic dimensions, driving interpatient variability in treatment response and prognosis and creating an efficacy bottleneck for standardized therapies [6,7].

Luminal epithelial cells or basal cells are recognized as key PCa cell of origin candidates [8], whose composition varies among patients and cannot be differentiated via visual inspection alone. Moreover, their interactions with the tumor microenvironment (TME), particularly cancer-associated Fibroblasts (CAFs), are a critical driver of disease progression [9]. However, conventional research approaches (e.g., immunohistochemistry and immunofluorescence) cannot elucidate the dynamic interplay between Luminal cells and the TME across distinct pathological microregions. Single-cell omics technologies have advanced our understanding of PCa heterogeneity [10], but their ability to generate dissociation-based natural strips of cells in a spatial context has given only limited insights into cell-cell interactions and microregion-specific functional states. Traditional spatial transcriptomics integrates histology with high-throughput sequencing [11], yet its low spatial resolution (confined to multicellular “spots”) does not allow the resolution of single-cell and subcellular features, which are critical for deciphering the precise mechanisms of Luminal subset evolution and TME crosstalk.

CosMx spatial molecular imaging (SMI) addresses these limitations by enabling mRNA profiling at single-cell and subcellular resolution while preserving tissue morphology [12]. This technology uniquely facilitates the simultaneous characterization of cellular heterogeneity and spatial localization (pathological microregions), making it ideal for dissecting the complex microenvironment of PCa. Despite progress, the evolutionary trajectory of Luminal cells from benign lesions (BPH/PIN) to malignant tumors (poorly differentiated PCa and prostatic adenocarcinoma with vacuolization) remains unclear. Moreover, how Luminal subsets dynamically interact with stromal components (e.g., Fibroblasts and Pericytes) across pathological stages to drive tumor progression, therapeutic resistance, and immune suppression remains largely unknown.

**Figure 1:**
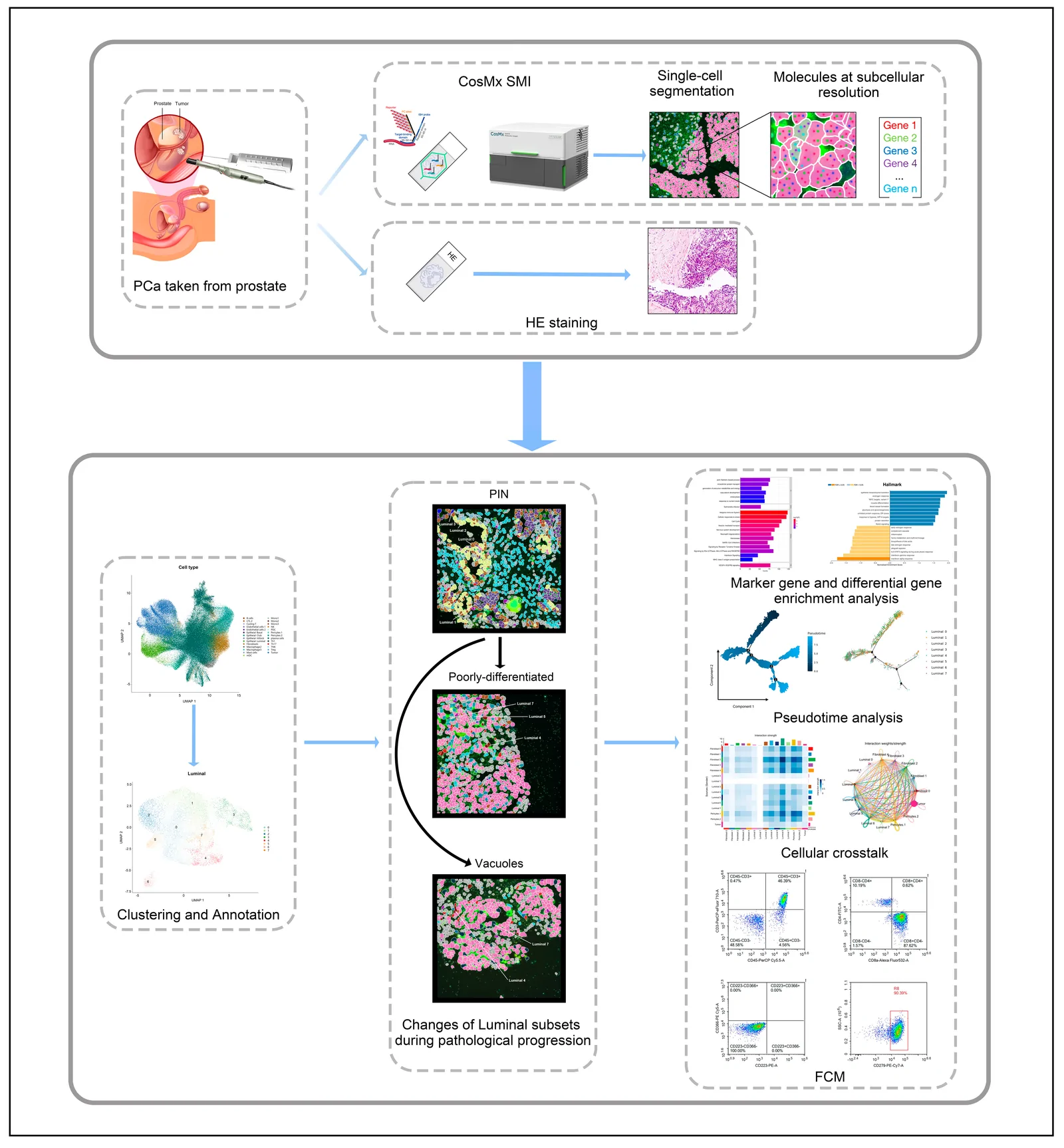
Schematic overview of the workflow for single-cell spatial transcriptomics.

To address the aforementioned research gaps, the present study centered on the multidimensional heterogeneity of PCa as the core research subject. Given that the spatial evolutionary trajectory of Luminal subsets and pathological microregion-specific epithelial–stromal communication across sequential prostate lesions has not been systematically characterized, we herein utilized high-resolution CosMx SMI (6K mRNA panel; NanoString Technologies, Washington, DC, USA) to construct a single-cell spatial transcriptomic atlas, which for the first time delineated Luminal evolutionary patterns and their crosstalk with the TME (Figure 1). By integrating pathological microregions with single-cell transcriptional profiling, our study compensated for the lack of spatially resolved tumor–stromal crosstalk analysis in the existing literature and yielded novel spatially defined insights into the stepwise progression of PCa.

## 2. Methods

### 2.1. Collection of PCa Specimens and Patient Consent

The research subjects were patients diagnosed with PCa at the Cancer Hospital, Chinese Academy of Medical Sciences, between December 2024 and June 2025. A total of 10 patients met the inclusion criteria and were enrolled.

**Inclusion Criteria:**

① Pathologically confirmed PCa via needle biopsy with no prior initial treatment (including endocrine therapy, radiotherapy, surgery, etc.);

② Age ≥ 18 years;

③ Willingness to cooperate with needle biopsy sample collection and clinical data follow-up.

Exclusion Criteria:

① History of other concurrent malignant tumors;

② Severe dysfunction of major organs such as the heart, liver, or kidneys;

③ Contraindications to needle biopsy (e.g., abnormal coagulation function, severe infection, etc.).

All procedures in this study were conducted in accordance with the ethical principles of the Declaration of Helsinki and were approved by the Ethics Committee of the Cancer Hospital, Chinese Academy of Medical Sciences (Approval No.: 24/562-4842). All patients provided full informed consent and signed a written informed consent form prior to enrollment.

### 2.2. Sample Collection and Processing

Tumor tissue samples were collected via ultrasound-guided transrectal or transperineal prostate needle biopsy. For the six patients P1–P6, 2–3 needle biopsy tissue samples were obtained, resulting in a total of 13 tumor tissue samples from needle biopsies. Immediately after collection, all the samples were placed in a fixative solution containing 4% paraformaldehyde (PFA) and fixed at room temperature for 24 h.

For the remaining four patients (P7–P10), one needle biopsy sample of normal prostate tissue and one of tumor tissue were collected from each patient, resulting in a total of 8 samples from needle biopsies. The biopsied tissues were placed in tumor tissue preservation solution.

Furthermore, clinical baseline data from the enrolled patients were systematically collected and included the following: ① bone metastasis status and ② other baseline characteristics (e.g., age and Gleason score (GS)). A database for establishing the sample–clinical data correspondence was constructed to ensure the clinical relevance of the subsequent analyses.

### 2.3. Pathological Region Classification

Cryostat-cut adjacent sections, corresponding to those used for CosMx SMI, were stained with H&E. All fields of view (FOVs) matched to tissue sections underwent blinded pathological microregion classification based on histological morphological phenotypes, which was independently completed by two senior pathologists. The lesions were stratified into six histological categories: BPH, LGPIN, HGPIN, well-differentiated PCa, poorly differentiated PCa (hereinafter abbreviated as T), and prostatic adenocarcinoma with vacuolization (hereinafter abbreviated as Vacuoles). Notably, prostatic adenocarcinoma with vacuolization is not an independent standardized pathological subtype; this term is only used herein to describe focal lesions with prominent cytoplasmic vacuolation occurring in conventional acinar prostate adenocarcinoma. Selection criteria: A single FOV contained only one pathological region, or the target pathological region accounted for ≥80% of the FOV. Final FOVs included in the analysis were BPH (*n* = 3), LGPIN (*n* = 7), HGPIN (*n* = 13), well-differentiated PCa (*n* = 4), T (*n* = 32), and Vacuoles (*n* = 8).

### 2.4. Single-Cell Spatial Transcriptome Profiling (CosMx SMI Platform)

The CosMx SMI platform enables in situ RNA detection in preserved tissues via barcoded probes, allowing subcellular-resolution multiplexed gene detection with retained morphology. In this project, the Human 6K Discovery RNA Panel and Human Universal Cell Segmentation Kit were used for cell marker labeling. Single-cell spatial transcriptomic profiling and partial data analysis were performed by Shanghai Biochip Co., Ltd., Shanghai, China.

Briefly, PCa FFPE tissues (5 μm sections) were processed per the CosMx SMI manual (MAN-10184): heat retrieval at 100 °C for 15 min; digestion with 3 µg/mL Proteinase K at 40 °C for 30 min; and 5 min of incubation in the dark with 0.001% fiducials in 2×SSC-T. Samples were stained with DAPI and antibodies against CD298/B2M, PanCK, and CD45 for morphology visualization and FOV selection.

A total of 385 H&E-matched FOVs were imaged on the CosMx instrument. After probe hybridization, the unbound probes were washed off, the fluorophore linkers were released under UV irradiation, and the fluorescent barcodes were detected and decoded.

### 2.5. Single-Cell Spatial Transcriptome Data Analysis

#### 2.5.1. Cell Population Clustering and Annotation

Dimensionality Reduction and Clustering: Seurat (v5.0.3) and Scanpy (v1.9.8) were used for joint analysis. Spatial single-cell expression data were preprocessed and subjected to PCA reduction, and the optimal principal components were selected for Leiden clustering. The results were visualized via t-distributed stochastic neighbor embedding (t-SNE) and uniform manifold approximation and projection (UMAP) dimensionality reduction.

Marker Gene Screening and Cell Type Annotation: Seurat identified cluster-specific marker genes (highly expressed in cluster cells, significantly upregulated vs. others) from intercluster differential gene expression analysis. A suitable reference dataset was chosen, and InSituType (likelihood model-based, extracts cell expression features for Bayesian classification) was used to perform cell typing via supervised learning for cell annotation.

#### 2.5.2. Pseudotime Analysis of the Luminal Subsets

Monocle v2.22.0 was used to construct pseudotime trajectories for target subclusters. Seurat v5.0.3 was applied to extract raw counts and filter the top 1000 highly variable genes (HVGs), which were normalized in Monocle with default functions. Genes with an average expression >0.5 detected in ≥50 cells were retained, after which differentialGeneTest was used to define the HVGs (adjusted qval < 0.0001) for cell ordering via orderCells. Trajectories were constructed by reduceDimension (default).

#### 2.5.3. Cell Colocalization and Cell–Cell Interaction Analysis

Cell-cell interaction networks were constructed on the basis of spatial location and cell type, and proximity interaction trends were inferred via observed and expected frequency calculations. Spatial cell distribution and shape (clustering and cell type-based filling) were plotted in Giotto v4.0.5 from the fluorescence images. CellChat v2.1.2 (CellChatDB.human) was used to analyze communication from the normalized data, while netVisual_circle and heatmaps were used to visualize differences in interactions.

#### 2.5.4. Functional Analysis of Luminal and Fibroblast Subsets

Marker genes were submitted to the Metascape web server (v3.5) [13], and the GO biological processes and KEGG pathways associated with the genes were selected for functional pathway enrichment analysis. Disease association analysis was performed with the DisGeNET database; tissue-specific expression analysis of genes was conducted using the PaGenBase database; and analysis of the regulatory relationships between upstream transcription factors and genes was carried out through the TRRUST database.

#### 2.5.5. Analysis of the DEGs in the Luminal Subsets

On the basis of the pathological distribution characteristics of different Luminal subsets, a stratified analysis of the DEGs was conducted.

For the Luminal 0–1/3–7 subsets, differential gene expression was compared for “PIN→T” pathological progression. For Luminal 0–1/3–5, the gene expression levels in the PIN pathological microregion were calculated as the average of their expression levels in the LGPIN and HGPIN samples. Owing to the absence of the Luminal 6 subset in the LGPIN samples, its gene expression levels in the PIN pathological microregion were directly adopted from those in the HGPIN samples; conversely, owing to the absence of the Luminal 7 subset in the HGPIN samples, its gene expression levels in the PIN pathological microregion were directly adopted from those in the LGPIN samples.

Since the Luminal 2 subset was not distributed in T, differential gene expression was compared for “BPH→HGPIN” pathological progression.

The differences in gene expression changes in each Luminal subset during the corresponding pathological progression were calculated. All DEGs were submitted to the WebGestalt platform [14], with Hallmark50 and biological process noRedundant selected for gene set enrichment analysis (GSEA). An emphasis was placed on analyzing the associations of the DEGs with “androgen therapy resistance” (referring to the “ANDROGEN_RESPONSE” pathway gene set from the MSigDB database) and “EMT” (referring to the “EPITHELIAL_MESENCHYMAL_TRANSITION” pathway gene set from the MSigDB database). Common genes related to androgen therapy resistance or EMT within each Luminal subset were screened using Venn diagrams (http://www.deepvenn.com).

### 2.6. Flow Cytometry (FCM) Analysis of Changes in T-Cell Subsets in Tumor Tissues

FCM detection and data analysis were performed by Guidon Pharmaceutics Ltd., Beijing, China. Briefly, freshly isolated PCa tumor tissues were minced and digested in RPMI-1640 medium (supplemented with 0.5% Type IV collagenase and 0.05% DNase I) at 37 °C to generate single-cell suspensions. After collection and washing, red blood cells were lysed with ACK buffer. The cells were counted and adjusted to 1 × 10^7^ cells/mL with 1× PBS and then aliquoted into tubes (100 μL, 1 × 10^6^ cells/tube).

The cells were stained with a Red Fixable LIVE/DEAD probe on ice in the dark, followed by 0.5 h of staining with the following surface antibodies: PerCP-Cy5.5-anti-CD45, PerCP-eFluor 710-anti-CD3, FITC-anti-CD4, Alexa Fluor 532-anti-CD8a, PE-anti-CD223, PE-Cy5-anti-CD366, PE-Cy7-anti-CD279. After being washed with 1× PBS and centrifuged at 600× *g* for 5 min, the cells were resuspended in 200 μL of 1× PBS. Data were acquired with a Cytek Aurora flow cytometer (Cytek Biosciences, Fremont, CA, USA) and analyzed with NovoExpress (v1.6.0).

## 3. Results

### 3.1. Construction of a Single-Cell Spatial Transcriptomic Atlas

A total of 10 patients with PCa were included in this study, and their clinical baseline data were systematically collected (Table S1). The aforementioned clinical data were in one-to-one correspondence with the sample information, providing comprehensive clinical background support for subsequent analyses of PCa tumor heterogeneity, gene expression characteristics, and other related aspects.

Systematic scanning was performed on the PCa tissue samples included in this study, yielding a total of 385 valid fields of view (FOVs). Following rigorous data quality control, 154,168 high-quality single-cell spatial transcriptomic datasets were ultimately retained, ensuring the reliability of the subsequent analyses (Table S2).

**Figure 2:**
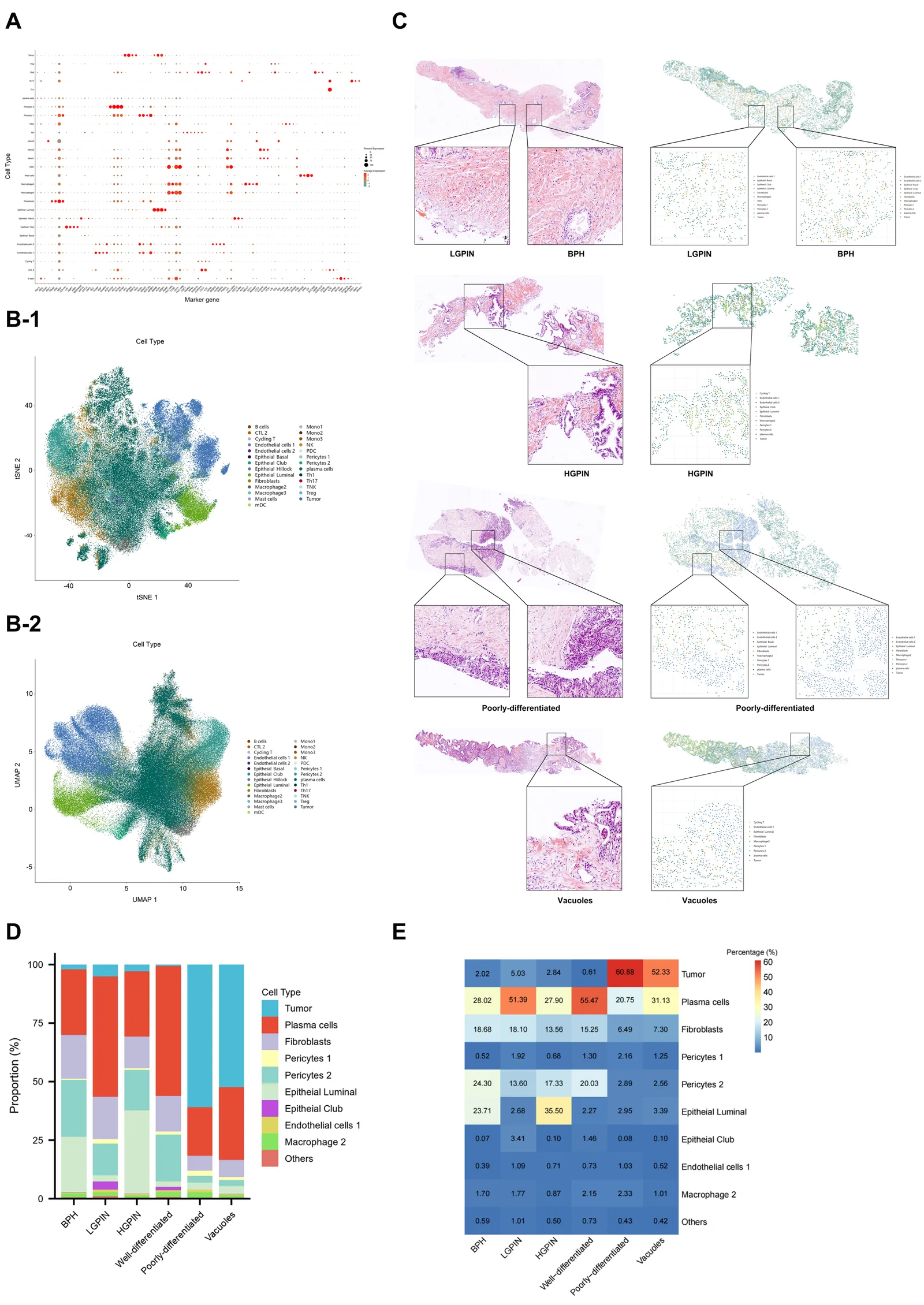
Construction of single-cell spatial transcriptomic atlas. (**A**) Dot plot illustrating the average expression level of marker genes across distinct cell types. tSNE (**B-1**) and UMAP (**B-2**) visualization plots of 154,168 single cells from six PCa patients, color-coded based on the cell types. (**C**) Single-cell subset spatial distribution images corresponding to the pathological microregions within adjacent H&E-stained sections. Stacked bar graph (**D**) and heat map (**E**) illustrating the proportional distribution of various cell types in six pathological microregions.

Following preprocessing, PCA dimensionality reduction, and unsupervised clustering, 28 cell clusters were identified, which were visualized via t-SNE and UMAP (Figure S1A,B). A spatial distribution map of cell clusters was constructed by combining the identified clusters with their original spatial coordinates (Figure S1C). The marker genes of these 28 major cell clusters (Figure 2A) allowed the subsequent annotation of these clusters into 27 distinct cell types, including epithelial Luminal cells, epithelial club cells, Pericytes 1, Pericytes 2, tumor cells, B cells, and CTL 2 cells (Figure 2B-1,B-2) [15]. Furthermore, the annotation results were matched with the spatial distribution maps (Figure S2), indicating the correspondence between the spatial localization and functional identities of cell types. This provides a spatial reference for subsequent analyses of TME cellular crosstalk and functional zoning.

A corresponding diagram of the different pathological microregions and cell types is shown in Figure 2C. Further analysis of the cell type composition across different pathological microregion revealed marked variations in the proportions across pathological states (Figure 2D,E): the proportions of tumor cells in T (60.88%) and Vacuoles (52.33%) were significantly greater than those in BPH and precancerous lesions (LGPIN and HGPIN both <6%), while the proportions of Luminal cell types were significantly greater in BPH (23.71%) and HGPIN (35.5%). The proportions of Fibroblasts gradually decreased with pathological progression (from 18.68% in BPH to 6.49% in T), and the Pericyte 2 cell type displayed a similar distribution pattern (with the highest proportion of 24.30% in BPH and significant reductions to 2.89% and 2.56% in T and Vacuoles, respectively). Furthermore, heterogeneity in cell type proportions was observed among different patients within the same pathological state, while the overall distribution trend remained consistent (Figure S3), verifying the reliability of the associations between cell type composition differences and pathological states, as well as the presence of interpatient heterogeneity.

### 3.2. Reclustering and Functional Enrichment Analysis of the Luminal and Fibroblast Subsets

#### 3.2.1. Reclustering of the Luminal and Fibroblast Subsets

**Figure 3:**
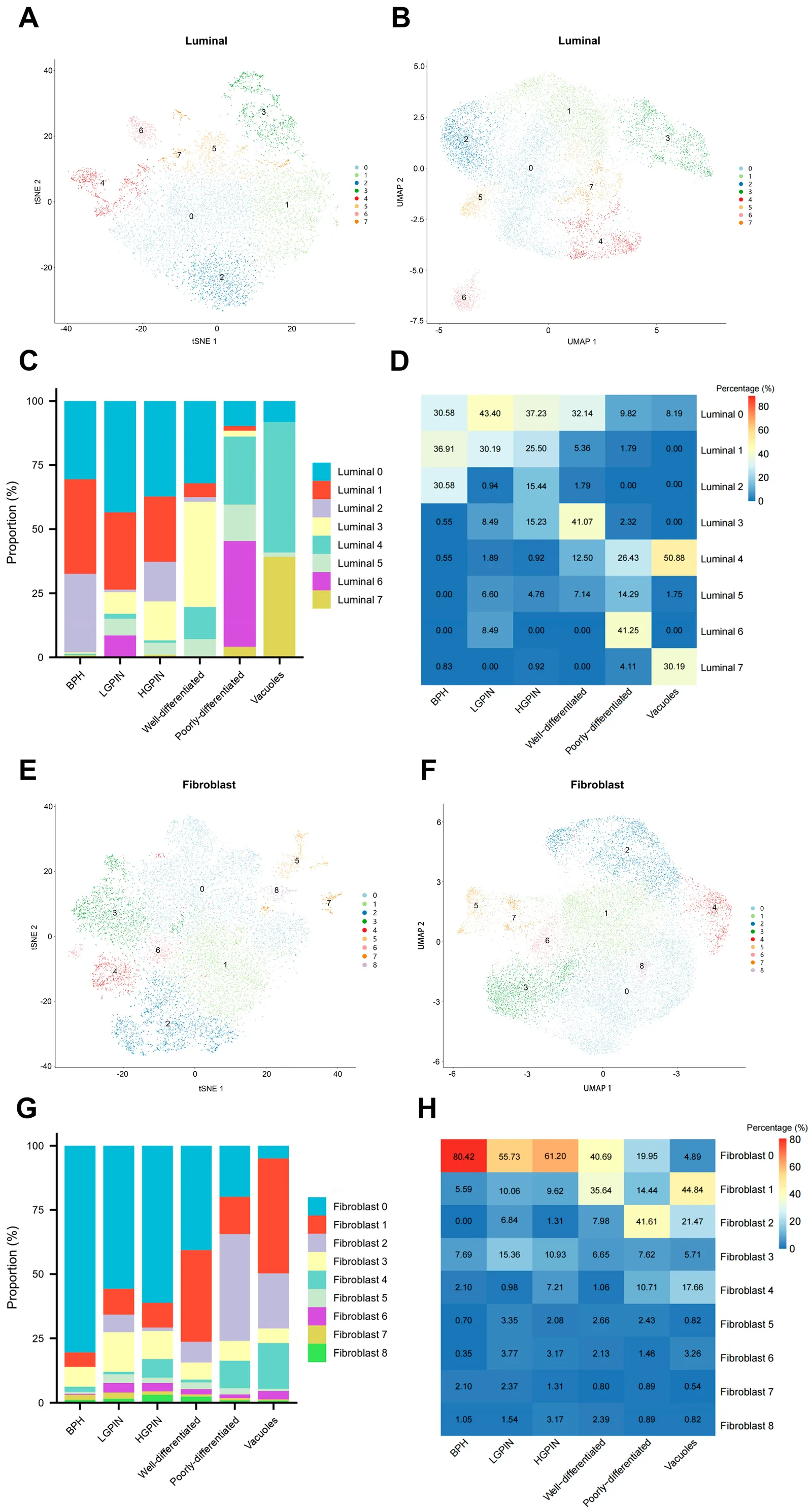
Re-clustering of Luminal and Fibroblast subsets. tSNE (**A**) and UMAP (**B**) dimensionality reduction plots depicting the two-dimensional distribution of eight PCa Luminal cell subsets. Stacked bar graph (**C**) and heat map (**D**) illustrating the proportional distribution of various Luminal cell subsets in six pathological microregions. tSNE (**E**) and UMAP (**F**) dimensionality reduction plots depicting the two-dimensional distribution of eight PCa Fibroblast cell subsets. Stacked bar graph (**G**) and heat map (**H**) illustrating the proportional distribution of various Fibroblast cell subsets in six pathological microregions.

Unsupervised clustering analysis of the Luminal subset revealed that it could be further divided into 8 subsets designated Luminal 0–7 (Figure 3A,B), whose spatial distributions are presented in Figure S4. Quantification of their proportions across different pathological microregions revealed the following (Figure 3C,D): the BPH and PIN samples were composed predominantly of Luminal 0–2; the T microregion had the highest proportions of Luminal subsets 4 and 6; and the Vacuoles microregion was dominated by Luminal 4, followed by Luminal 7. Heterogeneity in the proportions of Luminal subsets was also observed among different patients within the same pathological state. In addition to the abnormally elevated proportion of Luminal 6 cells in the T microregion of Patient 5, the overall distribution trend was consistent, suggesting that the high proportion of Luminal 6 cells in T might be attributed to this individual case (Figure S5).

Further unsupervised clustering of the Fibroblast subset yielded 9 subsets, Fibroblasts 0–8 (Figure 3E,F), whose spatial localizations are shown in Figure S6. Quantification demonstrated that each subset exhibited a pathological microregion-specific distribution pattern (Figure 3G,H). Fluctuations in the proportions of Fibroblast subsets were noted among different patients within the same pathological state, although the overall trend remained consistent (Figure S7).

#### 3.2.2. Marker Gene Enrichment Analysis of the Luminal and Fibroblast Subsets

**Figure 4:**
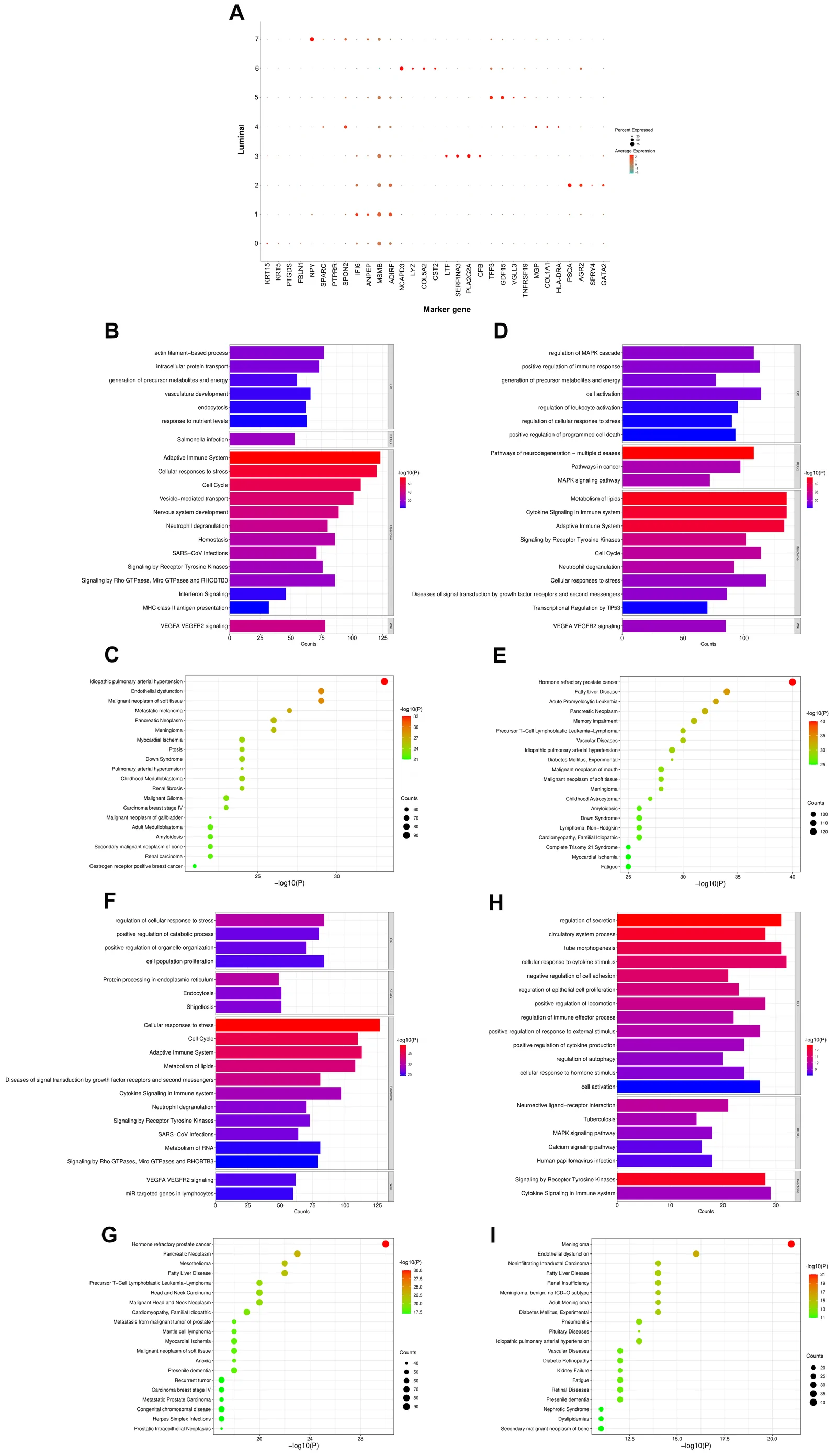
Marker gene enrichment analysis of Luminal subsets. (**A**) Dot plot illustrating the average expression level of marker genes in each Luminal subset. Bar plot illustrating functional enrichment analysis results of the Luminal 4 (**B**), Luminal 5 (**D**), Luminal 6 (**F**), and Luminal 7 (**H**) subsets. Dot plot depicting disease-related enrichment profiles of the Luminal 4 (**C**), Luminal 5 (**E**), Luminal 6 (**G**), and Luminal 7 (**I**) subsets.

To elucidate the functional heterogeneity of the Luminal subsets, the differential expression of distinct genes across different subsets was analyzed via single-cell spatial transcriptome sequencing, leading to the identification of a unique marker profile for each subset (Figure 4A, Figures S8 and S9). Functional enrichment analysis was performed to clarify the potential role of each subset in the initiation and progression of PCa. Luminal 4 was enriched in pathways such as the adaptive immune system, cell cycle, and vesicle-mediated transport and was associated with secondary malignant neoplasm of bone (Figure 4B,C). The marker genes of this subset were specifically expressed in LNCaP cells and regulated by transcription factors such as *SP1*, *TP53*, and *AR* (Figure S10A-1,A-2). There was enrichment of the Luminal 5 subset in multiple functional processes, including lipid metabolism, cytokine signaling in the immune system, and the adaptive immune system, and this subset was linked to hormone-refractory prostate cancer (Figure 4D,E). The marker genes of this subset were specifically expressed in prostate tissue and regulated by multiple genes, including *SP1*, *AR*, and *RELA* (Figure S10B-1,B-2). Pathways such as cellular responses to stress and the adaptive immune system were enriched in Luminal 6, and this subset was closely associated with hormone-refractory prostate cancer and tumor metastasis and recurrence (Figure 4F,G). The marker genes of this subset were regulated by factors such as *SP1*, *TP53*, and *AR* (Figure S10C-1,C-2). Luminal 7 exhibited enrichment in processes such as the regulation of secretion, negative regulation of cell adhesion, regulation of epithelial cell proliferation, and neuroactive ligand–receptor interactions and was associated with the secondary malignant neoplasm of bone (Figure 4H,I). The marker genes of this subset were specifically expressed in prostate tissue and regulated by genes such as *SP1* and *AR* (Figure S10D-1,D-2). Luminal 0 cells exhibited enrichment in development-related pathways and developmental cell lineages and were associated with nonmalignant lesions such as benign prostatic hyperplasia (Figure S11A,B). Luminal 1 exhibited enrichment in pathways such as protein processing in the endoplasmic reticulum, neutrophil degranulation, and cellular homeostasis and was linked to prostatic intraepithelial neoplasia (Figure S12A,B). Pathways such as cellular responses to stress and the adaptive immune system were enriched in Luminal 2, and this subset was associated with conditions such as hormone-refractory prostate cancer (Figure S13A,B). Luminal 3 exhibited enrichment in pathways such as cytokine signaling in the immune system and antigen processing and presentation and was linked to benign prostatic hyperplasia (Figure S14A,B). The cell/tissue-specific enrichment characteristics of the marker genes and the enrichment results of the regulatory genes in Luminal 0–3 are shown in Figure S15.

To further validate the cellular identity of Fibroblasts identified in this study, we comprehensively compared their transcriptional profiles against well-documented gene signatures of CAFs and normal Fibroblasts (NFs) from published literature. Multiple landmark works have defined a core panel of canonical CAF markers, including *FAP*, *α-SMA/ACTA2*, *MFAP5*, *COL11A1*, *TNC*, *PDPN*, *ITGA11* and *NG2* [16]. Additional genes governing angiogenesis and immune modulation—such as *PDGFRA*, *PDGFRB*, *FAP*, *NOTCH3*, *HES4* and *THY1*—are also reported to be markedly upregulated in CAFs [17]. Consistently, all Fibroblast subsets recovered from our tissue sections displayed variable upregulation of these core CAF markers, including *FAP*, *COL11A1*, *TNC*, *PDPN*, *PDGFRA*, *PDGFRB* and *THY1*.

We then performed overlap analysis between our Fibroblast marker genes and a curated pan-cancer signature resource comprising 592 CAF-specific and 321 NF-specific transcripts [18]. This comparison identified 83 shared genes between our Fibroblast marker and pan-cancer CAF signatures, while only 46 overlapped with the pan-cancer NF gene sets (Figure S16A). Considering substantial transcriptional heterogeneity of Fibroblasts across different solid tumor types, we further extracted NF signature genes from two independent public PCa datasets (GSE85606 and GSE68164) using cutoff criteria (|LogFC| > 1 and *p* < 0.05). Distinct NF transcriptional profiles were observed between the two datasets; thus, we merged their differential gene lists and obtained a set of 43 NF signature genes. Notably, zero overlapping genes were found when cross-referencing these 43 NF markers with our Fibroblast marker genes filtered by identical statistical thresholds (Figure S16B). Collectively, these preliminary results suggested that Fibroblasts captured in our spatial transcriptomic atlas predominantly exhibited molecular signatures characteristic of CAFs.

On this basis, to dissect the functional roles of distinct Fibroblast subsets during PCa initiation and progression, we referenced previous studies on CAF classification in solid tumors and PCa (6 subsets, including CAFmyo and CAFinfla) [17]. Functional annotation of the 5 major Fibroblast subsets was performed on the basis of marker genes (Figures S17–S19): Fibroblasts 0 and 4 exhibited characteristics of CAFmyo and CAFadi; Fibroblast 2 displayed features of CAFinfla and CAFendMT; and Fibroblast 3 showed traits of CAFap. Consistent with established CAF molecular profiles, our Fibroblast subsets shared hallmark marker genes of classic CAF subtypes, reinforcing that they transcriptionally resemble CAF rather than NF. Subsequent functional enrichment analysis, disease association analysis, and upstream transcription factor prediction were conducted on the basis of specific marker genes, and the results are presented in Figures S20–S28.

### 3.3. Analysis of the DEGs During Luminal Subset Progression

#### 3.3.1. DEG Enrichment in Each Luminal Subset in Different Pathological Microregions

During the benign-to-malignant progression of prostate tissue, the enrichment of DEGs in each Luminal subset exhibited distinct characteristics. Given that the Luminal 2 subset was barely detectable in the T microregion among the clinical samples collected in this study, the changes in Luminal 2 in this section focus on the transition from BPH to HGPIN. In contrast, the alterations in the remaining Luminal subsets were investigated primarily for their transformation from PIN to T. Combined with pathway enrichment and functional analyses, the potential role of each Luminal subset in malignant tumor progression was inferred. The results are as follows:

**Luminal 0 Subset:**

**Figure 5:**
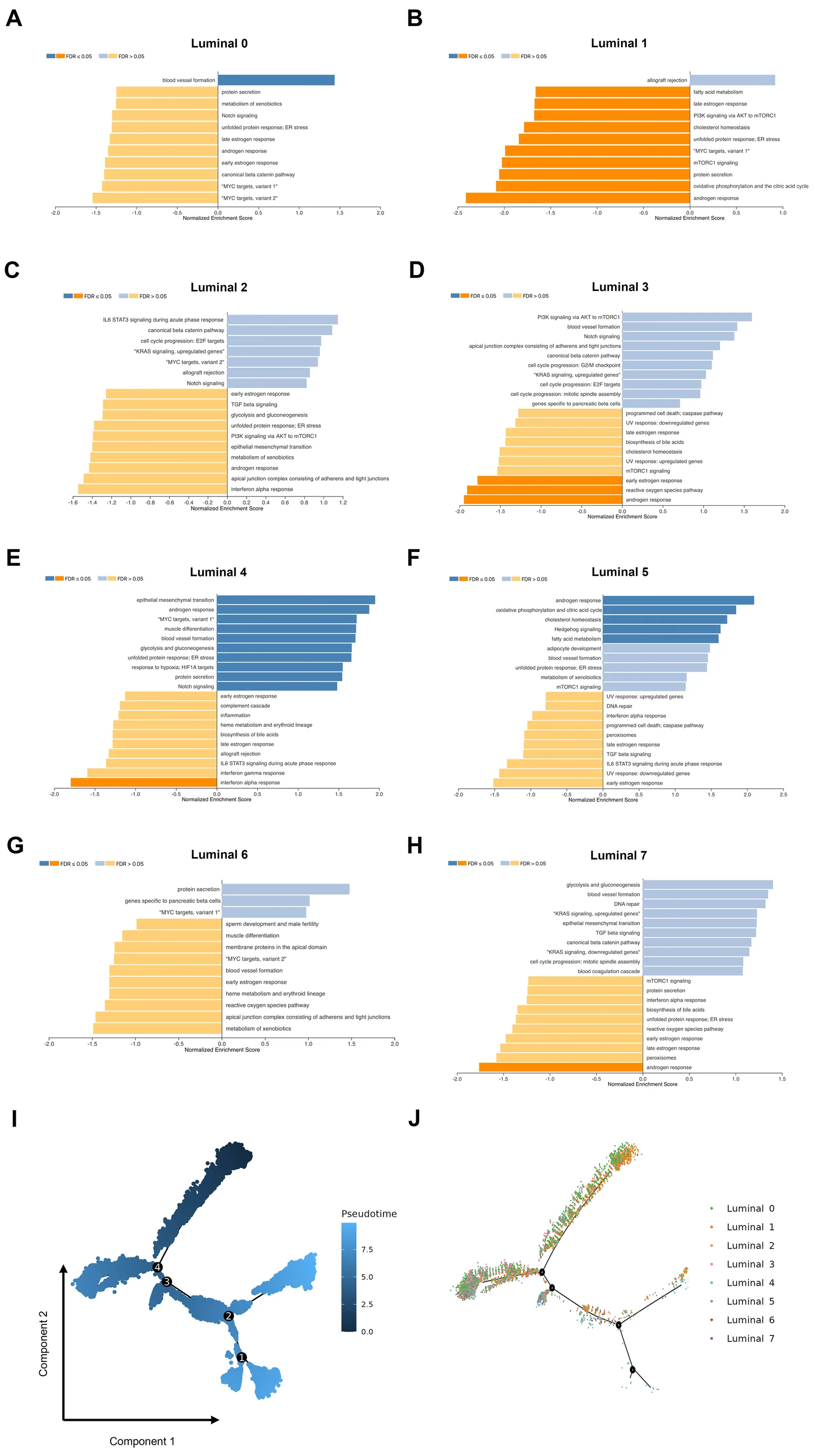
DEGs enrichment of each Luminal subset in different pathological microregions and pseudotime analysis of each Luminal subset. (**A**–**H**) GSEA showing significantly (FDR-corrected) enriched hallmark gene sets within Luminal 0–7 subsets. Graphs depicting the dynamic trajectories of Luminal cells, with color coding according to pseudotime (**I**) and cell subset classifications (**J**).

The tumor-related pathway enrichment results of this subset indicated that the blood vessel formation pathway was significantly activated (FDR ≤ 0.05). In contrast, multiple oncogenic pathways, including the canonical beta-catenin pathway and the Notch signaling pathway, were suppressed to varying degrees. Additionally, pathways associated with the hormone response (androgen/estrogen response pathway), as well as those related to protein secretion, endoplasmic reticulum stress, and protein homeostasis, exhibited different levels of inhibition (Figure 5A). Cell biological function enrichment analysis revealed the activation of multiple pathways closely linked to tumor initiation and progression, including postreplication repair. Conversely, pathways involved in mRNA and protein processing and transport, such as mRNA processing and protein-RNA complex organization, were suppressed (Figure S29A).

**Luminal 1 Subset:**

Tumor-related pathway enrichment analysis of this subset demonstrated that multiple tumor-related pathways (FDR ≤ 0.05), including oxidative phosphorylation, the citric acid cycle, and mTORC1 signaling, were significantly suppressed. In addition, the androgen/estrogen response pathway associated with the hormone response, as well as pathways related to protein secretion, endoplasmic reticulum stress, and protein homeostasis, were significantly suppressed (Figure 5B). In the cell biological function enrichment analysis, pathways such as lymph vessel development and integrin activation were activated. In contrast, pathways related to protein localization, transport, mRNA processing, and metabolism, including protein localization to the endoplasmic reticulum and Golgi vesicle transport, exhibited relatively significant suppression (Figure S29B).

**Luminal 2 Subset:**

Tumor-related pathway enrichment analysis of this subset revealed the activation of multiple oncogenic pathways, including IL6-STAT3 signaling, the canonical beta-catenin pathway, and KRAS signaling. In contrast, pathways that play critical roles in tumors, such as PI3K signaling via AKT to mTORC1, EMT, and TGF-beta signaling, were suppressed. Additionally, both the androgen/estrogen response pathway associated with the hormone response and the interferon alpha response pathway, which is linked to immune suppression, were inhibited (Figure 5C). According to the results of the cell biological function enrichment analysis, activation of the protein neddylation pathway can promote tumor progression through the regulation of protein modification. Conversely, pathways closely associated with tumor growth and metastasis, including polysaccharide metabolic processes and lymph vessel development, were suppressed to varying degrees (Figure S29C).

**Luminal 3 Subset:**

Tumor-related pathway enrichment analysis of this subset revealed that multiple pathways were activated to varying degrees, including PI3K signaling via the binding of AKT to mTORC1, blood vessel formation, Notch signaling, the canonical beta-catenin pathway, and KRAS signaling. In contrast, the androgen/estrogen response pathway associated with the hormone response was significantly suppressed. The inhibition of pathways such as the reactive oxygen species pathway, the UV response, and programmed cell death further promoted the development of malignant tumor phenotypes (Figure 5D). In the cell biological function enrichment analysis, protein neddylation was significantly activated (FDR ≤ 0.05). The activation of pathways such as the regulation of the response to extracellular stimuli and the inhibition of the cellular senescence pathway further facilitated malignant tumor progression. Additionally, pathways related to mRNA metabolism, protein secretion, and localization were suppressed (Figure S29D).

**Luminal 4 Subset:**

Tumor-related pathway enrichment analysis of this subset revealed that multiple tumor-related pathways, including EMT, the androgen response, MYC targets, variant 1, glycolysis and gluconeogenesis, the response to hypoxia, HIF1A targets, blood vessel formation, Notch signaling, and muscle differentiation, were significantly activated (FDR ≤ 0.05). In contrast, the interferon alpha/gamma response pathway, which is closely associated with antitumor immunity, was significantly suppressed (Figure 5E). In the cell functional enrichment analysis, pathways related to cellular metabolism and protein synthesis and transport, such as translational initiation and pyruvate metabolic processes, were activated. Conversely, pathways closely linked to antitumor immunity, including antigen processing and presentation, MHC protein complex assembly, and the adaptive immune response, were significantly inhibited (Figure S29E).

**Luminal 5 Subset:**

Tumor-related pathway enrichment analysis of this subset demonstrated that multiple hormone-responsive and metabolism-associated oncogenic pathways, including those involved in the androgen response, oxidative phosphorylation and the citric acid cycle, Hedgehog signaling, and fatty acid metabolism, were significantly activated (FDR ≤ 0.05). In addition, the inhibition of pathways such as the UV response and programmed cell death further promoted tumor progression (Figure 5F). In the cell functional enrichment analysis, pathways related to energy metabolism and ion transport, such as proton transmembrane transport, were activated, supporting rapid tumor proliferation and metabolic reprogramming. Conversely, the inhibition of pathways, including mitochondrial gene expression, also played important roles in tumor energy metabolism and proliferation (Figure S29F).

**Luminal 6 Subset:**

Tumor-related pathway enrichment analysis of this subset revealed the activation of two pathways closely associated with cell proliferation and the TME: MYC targets, variant 1, and protein secretion, which provide the impetus for tumor growth and invasion. Although two other oncogenic pathways, MYC targets, variant 2, and blood vessel formation, were suppressed, it is hypothesized that the activation of oncogenic pathways may serve as the core protumorigenic driver (Figure 5G). In the cell functional enrichment analysis, the activation of oncogenic pathways, such as DNA catabolic processes and translational initiation, facilitated tumor cell proliferation. In contrast, the inhibition of the response to the pH pathway may further affect the progression of malignant tumor phenotypes by disrupting pH homeostasis in the internal microenvironment (Figure S29G).

**Luminal 7 Subset:**

Tumor-related pathway enrichment analysis of this subset revealed that multiple classic oncogenic pathways were activated, driving tumor growth and metastasis through multiple mechanisms. These pathways include glycolysis and gluconeogenesis, blood vessel formation, KRAS signaling, EMT, TGF-beta signaling, and the canonical beta-catenin pathway. In contrast, the androgen/estrogen response pathway associated with the hormone response was relatively significantly suppressed, and pathways such as protein secretion were also inhibited (Figure 5H). According to the results of the cell functional enrichment analysis, the phagosome maturation pathway was significantly activated and may be involved in shaping the tumor immune microenvironment. Oncogenic pathways such as protein kinase C signaling can promote tumor proliferation and invasion by regulating cellular signaling and metabolism. Additionally, pathways related to protein secretion and localization were suppressed to varying degrees (Figure S29H).

#### 3.3.2. Correlation Analysis of the DEGs in Each Luminal Subset with Androgen Therapy Resistance and EMT

Enrichment analysis of the DEGs in each Luminal subset during the transformation of prostate tissue from PIN to T revealed that DEGs associated with androgen therapy resistance and EMT may play pivotal roles in this malignant transformation. Cross-analysis of the Luminal 0, 1, 3, and 7 subsets, identified by enrichment analysis to be associated with androgen therapy resistance, revealed that *TMPRSS2*, *NKX3-1*, *AZGP1*, and *SPDEF* were common significantly DEGs and may serve as core candidate genes regulating resistance to androgen therapy. Analysis of the Luminal 4 and 7 subsets, previously confirmed to be associated with EMT, revealed that *VEGFA*, *MGP*, *SPARC*, *BGN*, *COL4A1*, *COL4A2*, *COL5A3*, *TGM2*, *FN1*, *MYL9*, *WNT5A*, and *PLOD2* were DEGs shared by the EMT pathways in these two subsets. These genes are strongly associated with EMT and promote the malignant progression of tumors.

To systematically evaluate epithelial–mesenchymal plasticity across all eight Luminal subsets identified in our spatial transcriptomic dataset, we quantified the expression intensities using a well-validated 16-gene canonical EMT signature. This signature comprises 13 mesenchymal markers (*VIM*, *CDH2*, *FOXC2*, *SNAI1*, *SNAI2*, *TWIST1*, *FN1*, *ITGB6*, *MMP2*, *MMP3*, *MMP9*, *SOX10*, *GCS*) and 3 epithelial markers (*CDH1*, *DSP*, *OCLN*) [19]. Among all Luminal subsets, Luminal 7 displayed the most evident EMT activation phenotype, featured by concurrent downregulation of core epithelial genes *CDH1* and *DSP*, accompanied by elevated expression of multiple mesenchymal signature genes including *VIM*, *SNAI2*, *FN1* and *SOX10*. Luminal 4 exhibited a distinct hybrid partial EMT phenotype, which harbored the largest number of upregulated mesenchymal markers while retaining high expression of epithelial markers *CDH1* and *DSP*, implying the strongest epithelial–mesenchymal plasticity among all Luminal subsets. In contrast, Luminal 0,1,3,5,6 and Luminal 2 generally presented overall low mesenchymal gene expression and mild reduction in epithelial markers, lacking clear transcriptional signatures of EMT activation. Collectively, these graded patterns of EMT activation and inhibition were consistent with the results of DEGs enrichment analysis (Figure S30).

#### 3.3.3. Pseudotime Analysis of Each Luminal Subset

To elucidate the developmental lineage relationships and state transition trajectories of each Luminal subset within the TME of PCa patients, pseudotime analysis was performed on the Luminal subsets to reconstruct their dynamic differentiation trajectories (Figure 5I,J and Figure S31). The results revealed that each subset exhibited a pathological microregion-specific distribution pattern. Among them, Luminal 0, 2, and 3 shared similar developmental stages and differentiation directions and all were concentrated in the early-to-middle developmental stages; Luminal 1 was enriched mainly in the early-to-middle stages, with a small distribution also present in the terminal stage; and Luminal 4, 5, 6, and 7 were distributed predominantly in the middle developmental stage, yet subtle differences existed among these subsets: Luminal 4, 5, and 7 were also sporadically distributed in the early and terminal stages, while Luminal 6 was more restricted to the middle stage.

### 3.4. Colocalization and Crosstalk of Luminal and Fibroblast Subsets with Other Cell Types

#### 3.4.1. Colocalization of Luminal and Fibroblast Subsets with Other Cell Types

**Figure 6:**
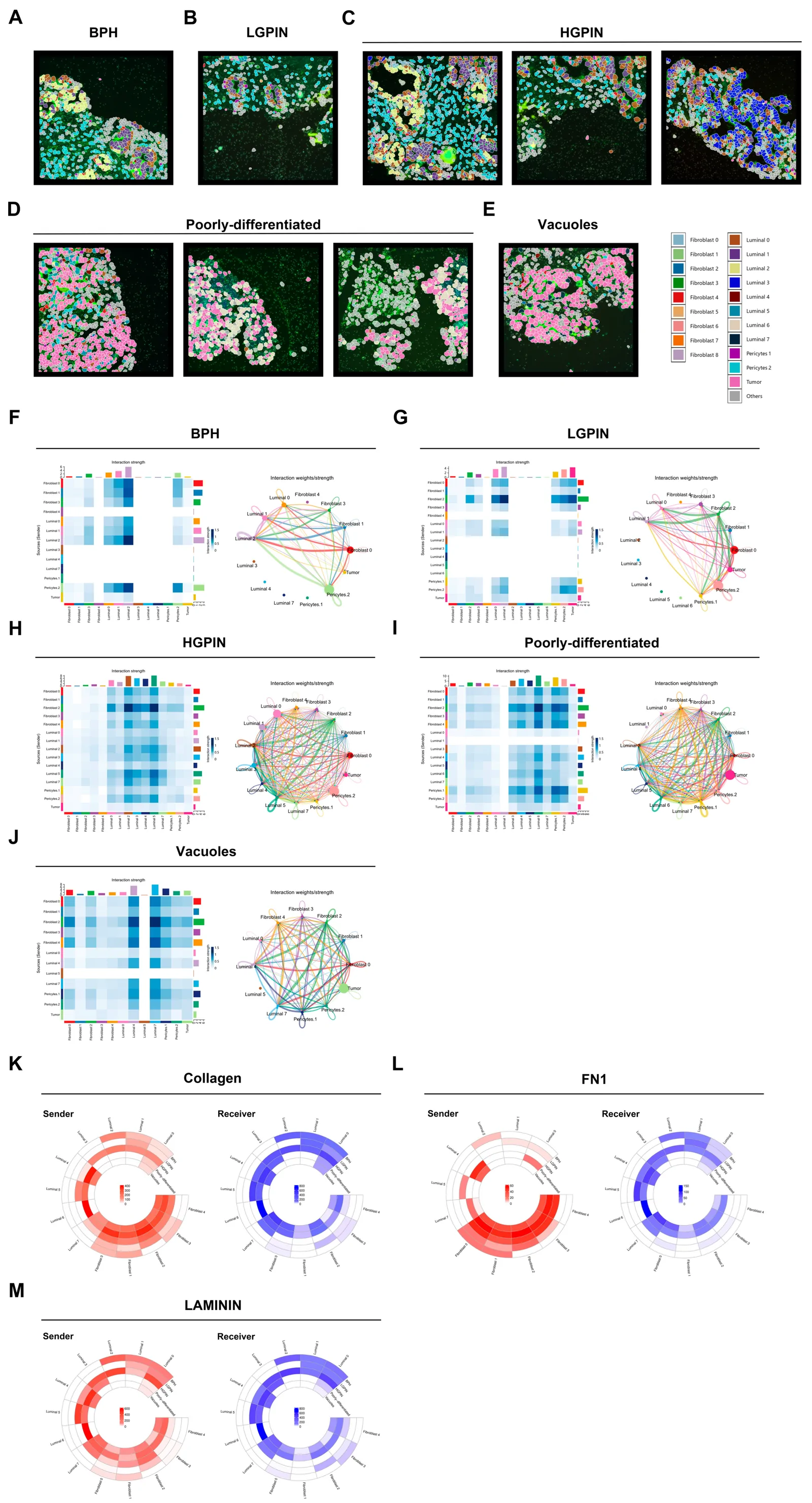
Colocalization and cellular crosstalk of Luminal and Fibroblast subsets with other cell types. (**A**–**E**) The distribution and shape of cells were plotted spatially in situ combined with fluorescent images across five pathological microregions, and the cells were filled according to cell types. (**F**–**J**) Heatmap and circle plots showing interaction strengths among Luminal, Fibroblast, Pericyte, and tumor subsets in five pathological microregions. (**K**–**M**) Circular heatmap showing the number of intercellular interaction patterns, where Luminal and Fibroblast subsets act as senders and receivers in three signaling pathways across five pathological microregions.

In the BPH pathological microregions, Luminal 0–2 cells constituted the predominant cells of the glandular Luminal epithelium, while Fibroblasts 0, 1, and 3 were distributed around it. Pericytes 2 were abundantly distributed among glandular tissues and adjacent to multiple cell types, with a small number of Tumor cells scattered among Luminal subsets (Figure 6A). In the LGPIN pathological microregions, Luminal 0 and 1 constituted the main component of the glandular Luminal epithelium, and Fibroblast 0–3 were sporadically distributed around it or among glandular tissues. The distribution of Pericytes 1 was greater than that in the BPH pathological microregions, and this subset was concentrated around the basal layer (Figure 6B). In the HGPIN pathological microregions, the Luminal subset formed the main body of the glandular Luminal epithelium but exhibited high intersample heterogeneity (some were dominated by Luminal subsets 0, 1, and 2, while others were predominantly composed of subsets 0, 1, 5 or 0, 1, and 3). Fibroblasts 0–4 were sporadically distributed around it or among glandular tissues, and Pericytes 1 and 2 were located among glandular tissues (Figure 6C). In the T pathological microregions, the normal glandular lumen structure disappeared, and Tumor cells became the dominant cell type. In the majority of the T pathological microregions, Fibroblast 0–4, Luminal 4, 5, 7, and Pericytes 1 and 2 were sporadically distributed around Tumor cells. Interestingly, many sporadically distributed Luminal 6 cells were detected in the T pathological microregions from the same patient, and these microregions exhibited aggressive pathological features, including cribriform or intraductal carcinoma (Figure 6D). In the Vacuoles pathological microregions, Tumor cells still predominated, with Fibroblast 0–4, Luminal 4 and 7, and Pericytes 1 and 2 scattered around them. Compared with those of the T pathological microregions, the proportions of Luminal 4 and 7 increased in the Vacuoles pathological microregions, while the proportions of Pericytes 1 and 2 decreased (Figure 6E).

#### 3.4.2. Crosstalk of Luminal and Fibroblast Subsets with Other Cell Types

To investigate the interaction patterns of cell subsets in prostate tissue, cell communication heatmaps and network node diagrams were generated based on the ligand-receptor interaction analysis in this study. The characteristics of each pathological microregion were as follows:

In the BPH pathological microregions, Fibroblast 0 exhibited moderate to high intensity interactions with Luminal 0–2 and Pericytes 2. Fibroblasts 1 and 3 showed distinct interactions with Luminal 0–2 cells. Luminal 2 displayed high interaction intensities with Fibroblast 3 and Luminal 0–2, exerting both intraepithelial and epithelial–stromal communication functions. Pericytes 2 strongly interacted with Luminal 0–2 and autologous cells, whereas Tumor cells showed relatively weak interactions with other cell types (Figure 6F).

In the LGPIN pathological microregions, Fibroblast 0–3, Luminal 0–1, Pericytes 1–2, and Tumor cells were the core participants in cell communication, with significant differences in interaction intensity among the subsets. Fibroblasts 0 and 2 (especially Fibroblast 2) exhibited moderate to high intensity interactions with multiple subsets. Luminal 0–1 had moderate intensity interactions with Fibroblasts 2–3 and with each other. Moderate-intensity interactions were observed between Pericytes 1–2, Luminal 0–1, and Tumor cells, with Tumor cells acting as signal receivers to drive tumor progression (Figure 6G).

In the HGPIN pathological microregions, all the Fibroblast, Luminal, Pericyte, and Tumor cell types were involved in cell communication. Fibroblast 2 had the greatest interaction intensities, whereas Fibroblasts 0 and 4 and the other subsets exhibited moderate to high intensity interactions with multiple Luminal subsets. Luminal 2, 5, and 7 displayed active interactions among themselves. Overall, Pericytes and Tumor cell types had relatively weak interactions with other cell types or subsets and played auxiliary roles in cell communication (Figure 6H).

In the T pathological microregions, Fibroblast 0–4, Luminal 3–7, Pericytes 1–2, and Tumor cell types served as the core communication participants, whose interaction intensity was significantly greater than that in precancerous lesions. Fibroblast subsets (especially 0, 2, and 4) exhibited active interactions within their subset and with other cell types. Tumor cell types had weak but extensive interactions with multiple subsets, and the heterogeneity and intensity of cellular crosstalk were further exacerbated (Figure 6I).

In the Vacuoles pathological microregions, all the Fibroblast subsets, Luminal subsets 0, 4, and 7, Pericytes 1 and 2, and Tumor cell types were the core communication participants. Fibroblasts 0, 2, and 4 significantly interacted with Luminal 4, Luminal 7, Pericytes, and Tumor cells, suggesting that Luminal 4 and 7 may act as the core of the “epithelial–stromal–pericyte” interaction network (Figure 6J).

Analysis of the top signaling pathways revealed that the COLLAGEN, FN1, and LAMININ pathways were the pathways most strongly associated with tumor invasion and metastasis. In the COLLAGEN pathway, Luminal subsets mostly acted as signal receivers, whereas Fibroblast subsets primarily served as signal senders (Figure 6K and Figure S32A). The interaction patterns of Luminal 0–2 gradually diversified during the transition from BPH to HGPIN, and those of Luminal 4–7 increased significantly during the progression from HGPIN to T. Luminal 0 participated in COLLAGEN pathway interactions across all pathological microregions. Luminal 4 and 7 also exhibited abundant, high-intensity interactions in the Vacuoles pathological microregions. The interaction patterns of the Fibroblast subsets gradually increased with malignant progression; the interaction patterns of Fibroblasts 0 and 2 were the most diverse and exhibited the greatest interaction intensity across all the microregions, indicating that these cells play critical roles in pathological progression.

The trends of cellular crosstalk in the LAMININ pathway were similar to those in the COLLAGEN pathway. However, the Luminal subsets exhibited more diverse interaction patterns with higher intensities, whereas the interaction patterns and intensities of the Fibroblast subsets were generally reduced. The Luminal subsets acted as both senders and receivers and could also participate in interactions as influencers and mediators. Fibroblast subsets were mainly senders, except in HGPIN, where they were almost exclusively senders; Fibroblasts exhibited all four identities in other microregions (Figure 6L and Figure S32B). In terms of progression characteristics, the Fibroblast subsets showed weak interactions in BPH, LGPIN, and HGPIN, but their interaction patterns and intensities increased significantly in T/Vacuoles pathological microregions, with these changes being more pronounced in the LAMININ pathway.

In the FN1 pathway, the interaction intensity of the Luminal and Fibroblast subsets was significantly lower than that in the other two pathways, and their interaction characteristics were more similar to those in the COLLAGEN pathway. Cell subsets strongly participated in this pathway only in the LGPIN, T, and Vacuoles pathological microregions. Luminal subsets were mainly receivers (only Luminal 4 exhibited the identities of receiver, influencer, and mediator, whereas most others were receivers and mediators). The Fibroblast subsets were predominantly senders, influencers, and mediators, with Fibroblasts 0 and 2 potentially being the core subsets (Figure 6M and Figure S32C).

### 3.5. Changes in T Lymphocytes in Tumor Tissues

The FCM results revealed that both normal prostate tissues and PCa tissues presented an immunosuppressive state, as detailed below.

**Figure 7:**
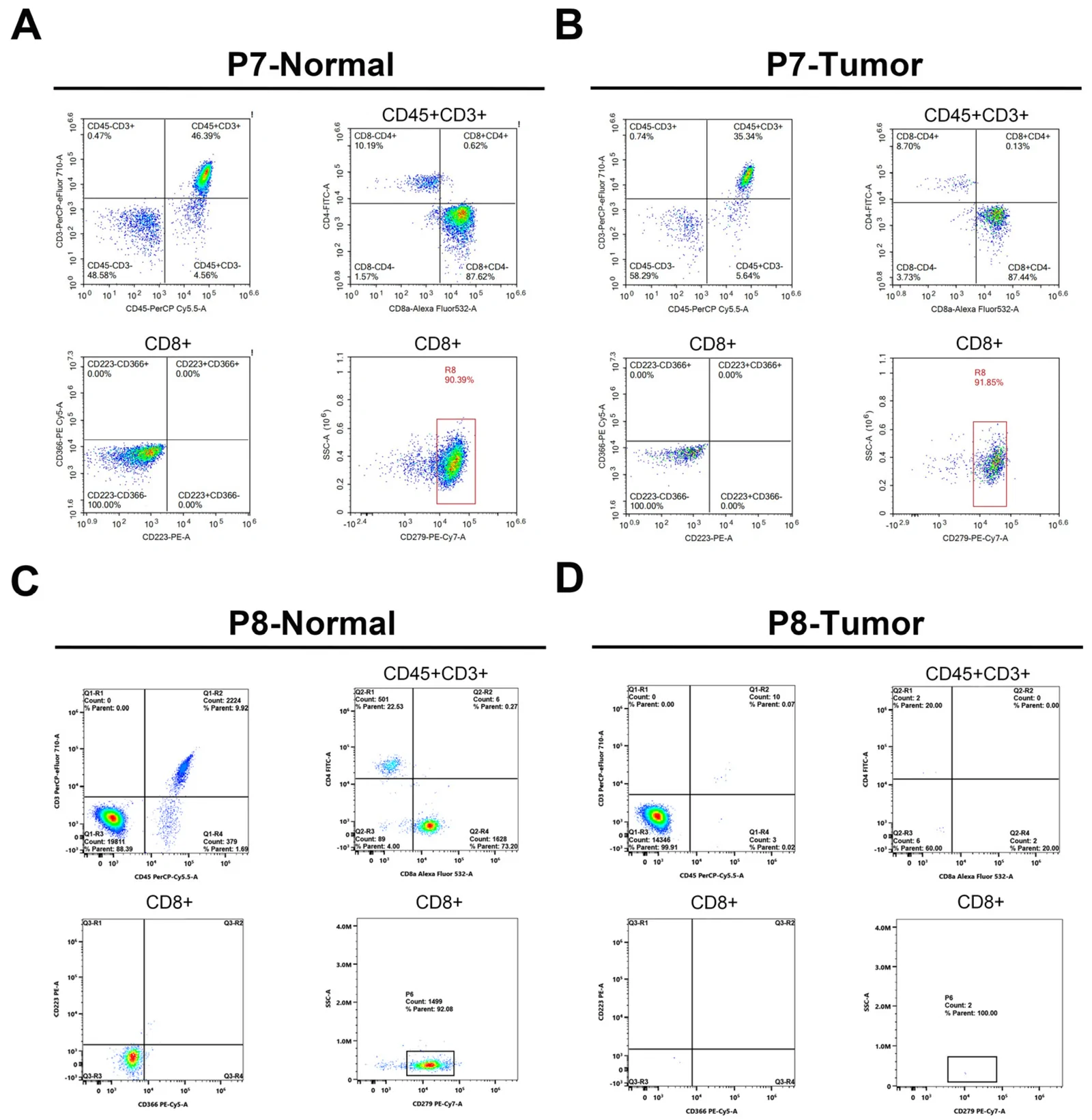
Changes in T lymphocytes in tumor tissues. Proportions and exhaustion status of T cells in normal tissue (**A**) and PCa tissue (**B**) of Patient 7. Proportions and exhaustion status of T cells in normal tissue (**C**) and PCa tissue (**D**) of Patient 8.

With respect to the T lymphocytes from the normal tissue samples from Patient 7, CD8^+^ T cells accounted for 87.62%, with high PD-1 (CD279) positivity (positive rate: 90.39%) (Figure 7A); in the PCa tissue samples from Patient 7, T lymphocytes accounted for only 35.34% of the total cells, among which CD8^+^ T cells constituted 87.44%, with high PD-1 positivity (positive rate: 91.85%), while both LAG3 (CD223) and TIM-3 (CD366) expression was negative (Figure 7B). T cells in both tissues were predominantly exhausted, suggesting that the tumor was immunosuppressive.

In Patient 8, a small number of T lymphocytes were present in normal tissues, among which CD8^+^ T cells accounted for 73.20%, and CD4^+^ T cells accounted for 22.53%. These T cells were also highly PD-1-positive (positive rate of CD8^+^ T cells: 92.08%) and CD223- and CD366-negative (Figure 7C); furthermore, almost no T-cell infiltration was observed in the PCa tissues (Figure 7D). Therefore, the majority of the T cells in this patient’s normal tissues were exhausted, and nearly no T lymphocyte infiltration was detected in PCa tissues.

P9 and P10 exhibited similar results. The detailed results are shown in Figure S33.

In summary, normal prostate tissues contain a small number of CD4^+^/CD8^+^ T lymphocytes, but most of them are in an exhausted state. PCa tissues can be divided into two categories: those with T-cell infiltration, wherein most cells are exhausted, and those with almost no T-cell infiltration. Both conditions indicated that the PCa tissue and its surrounding microenvironment were in an immunosuppressive state.

## 4. Discussion

In this study, CosMx SMI was utilized to construct a comprehensive single-cell spatial transcriptomic atlas of PCa. Pathological microregions were classified according to histomorphological features under light microscopy; we systematically characterized the features and spatiotemporal evolutionary patterns of diverse Luminal subsets, as well as their interactions with stromal cells. All results of the present study were derived from observational data including CosMx SMI sequencing, pathway enrichment analysis, pseudotime trajectory inference, and cell–cell communication prediction. No in vitro or in vivo functional experiments were performed for validation. Accordingly, all interpretations presented below are merely exploratory hypotheses based on single-cell spatial transcriptomic data, rather than validated biological mechanisms.

### 4.1. Distribution and Functional Heterogeneity of Luminal Subsets in Distinct Pathological Microregions

Prostate gland functional integrity relies on Luminal epithelial cells, whose subtype dynamics might reflect pathological evolution. Our study revealed 8 Luminal subsets (Luminal 0–7) with specific pathological distribution patterns: Luminal 0–2 dominated in BPH and PIN, forming glandular lumen structures, whereas Luminal 3–7 were enriched in T and Vacuoles microregions; however, these regions were sporadically distributed. Such compositional shifts in Luminal subsets across distinct pathological microregions merely represented statistically correlated molecular signatures observed in the present study. They may serve as potential biomarkers to distinguish precancerous lesions from malignant tumors, yet further validation via independent patient cohorts and functional experiments is still required.

Pseudotime trajectory analysis further clarified the developmental lineage of the Luminal subsets: Luminal 0, 2, and 3 clustered in early-to-middle differentiation stages, whereas Luminal 4–7 occupied the middle-to-terminal stages. This suggested that the aforementioned compositional shifts in Luminal subsets may occur during the progression of PCa.

We observed prominent pathway alterations across distinct Luminal subsets based on spatial single-cell transcriptomic analyses in this study. Functional enrichment analysis of marker genes derived from each Luminal subset showed that Luminal 0 was enriched in pathways related to developmental regulation (e.g., developmental cell lineage and mesenchyme development) and tissue homeostasis (e.g., extracellular matrix organization). Restricted to nonmalignant pathological microregions, Luminal 0 participated in sustaining normal prostate tissue architecture. Notably, this subset lacked enrichment of core immune pathways, a molecular signature that distinguished it from other Luminal subsets. By contrast, Luminal 1–3 and 5–7 all shared the core pathway “cytokine signaling in the immune system”, indicating their potential association with microenvironmental inflammatory responses and tumor progression, such as inflammatory stimulation-triggered IL-6 secretion that facilitates tumor proliferation and bone metastasis [20].

### 4.2. Differential Transcriptional Signatures of Luminal Subsets Along Pathological Progression

By comparing the enrichment profiles of DEGs in each Luminal subset during the progression from PIN to T, we revealed distinct activation or suppression patterns of signaling pathways across subsets. For instance, Luminal 4 exhibited significant enrichment of EMT, glycolysis and gluconeogenesis, the response to hypoxia, and MYC target pathways, all of which were markedly upregulated, whereas the interferon alpha/gamma response pathway was significantly suppressed. In Luminal 7, glycolysis and gluconeogenesis, KRAS signaling, EMT, and TGF-beta signaling pathways were upregulated, accompanied by reduced transcriptional activity of the androgen/estrogen response pathway. *TMPRSS2*, *NKX3-1*, *AZGP1*, and *SPDEF* were shared DEGs among multiple Luminal subsets with altered androgen response pathway. *VEGFA*, *SPARC*, *FN1*, and *COL*s represented common EMT-related DEGs specific to Luminal 4 and 7. Although pronounced downregulation of the androgen response pathway was observed in multiple Luminal subsets within this study, all enrolled specimens were localized primary PCa tissues obtained from treatment-naive patients without pathologically confirmed CRPC/mCRPC samples. The pathway enrichment results associated with CRPC pathogenesis in this manuscript were interpreted indirectly based on the MSigDB androgen response gene set and previously published literature only. Accordingly, these findings only reflected pretreatment transcriptional characteristics at the spatial single-cell levels in primary lesions and can merely serve as preliminary clues for exploring the mechanisms underlying castration resistance.

### 4.3. Dynamic Remodeling of Cell–Cell Communication Networks During Pathological Progression

Consistent with recent mechanistic research on CAFs, our core findings suggested that cell–cell communication networks within PCa underwent dynamic remodeling, which was governed by pathological-specific cell subsets and reflected functional rewiring of the TME. As key stromal components of the TME, CAFs exert multifaceted pro-tumor effects, including ECM remodeling, angiogenesis promotion, metabolic support for Tumor cells, and induction of immunosuppressive niches that facilitate immune evasion and limit drug penetration. Additionally, Therapeutic stress reshapes CAF functional identities and activates drug resistance-related signaling cascades [21]. Recent single-cell and spatial multi-omics studies have verified that distinct CAF subsets occupy spatially segregated microregions, and their functional traits shift drastically during tumor progression [22,23]. These published findings align closely with our data showing pathological microregion-specific crosstalk between Fibroblast-Luminal subsets.

Two well-established mechanistic frameworks further contextualize our spatial communication results. First, NGF secreted by CAFs acts as a pivotal mediator of stromal-epithelial crosstalk in PCa. NGF-initiated intracellular signaling endows tumor cells with enhanced epithelial plasticity and invasive capacity, drives tumor-induced neurogenesis, and mediates perineural invasion to sustain malignant stroma–tumor interplay [24]. Second, non-genomic AR signaling in CAFs forms functional complexes with Filamin A, which remodels ECM architecture via integrin-dependent pathways, elevates Fibroblast migratory potential, and constructs a permissive microenvironment to distant metastatic spread [25]. Together, these two mechanistic models reinforce the central functional role of CAFs within the PCa TME and provide a plausible molecular basis to interpret the spatially restricted cellular phenotypes uncovered in our spatial transcriptomic dataset.

We systematically described pathological microregion-specific communication architectures at the single-cell spatial transcriptome level.

(1) BPH: Fibroblast 0–3, Luminal 0–2, and Pericytes 2 form a stable communication core. (2) LGPIN: Network reconstruction begins, with Fibroblast 2 replacing Fibroblast 0 as the hub and tumor cells transitioning to signal receivers. (3) HGPIN: Core reinforcement and heterogeneity emerge, with Fibroblast 2 cooperating with Fibroblast 0 and 4 and Luminal subsets exhibiting functional differentiation, reflecting stromal–epithelial regulation of malignant potential. (4) T: A dense communication network forms, with Fibroblast 0, 2, and 4, Luminal 3–7, and Pericytes enabling extensive interactions that support invasion. (5) Vacuoles: A specialized signaling pattern dominates, with Fibroblast 0, 2, and 4 showing robust interactions with Luminal 4 and 7, suggesting that these subsets are core nodes of the “epithelial–stromal–pericyte” network in this rare pathological subtype.

Three core ECM-related ligand–receptor axes dominate intercellular communication across pathological microregions, with distinct activity patterns: COLLAGEN signaling pathway maintains a consistent Fibroblast-sender/Luminal-receiver axis, with Luminal 0 as the core subset in all states. Fibroblast 0 and 2 maintain high activity to regulate ECM remodeling. LAMININ complements COLLAGEN, with more diverse Luminal interactions and increased Fibroblast signaling in advanced tumors. FN1 signaling is only active in LGPIN, T, and Vacuoles, synergizing with the other two pathways to mediate pathological-microregion-specific phenotypes.

### 4.4. Integration with Existing Research: Advancing PCa Subtype Biology

Our findings build on and extend previous landmark studies in PCa subtype research.

**Bulk RNA-seq-based classification:** Previous research established the macroscopic classification system of PCa, including Luminal A, Luminal B, and Basal subtypes using the PAM50 classifier. Luminal B subtype was correlated with poor clinical prognosis and distinct therapeutic response profiles [26]. At single-cell spatial transcriptomic resolution, our study reveals that the highly malignant Luminal B subtype defined by bulk RNA-seq corresponds to Luminal subsets 4–7 identified herein. These subsets are enriched for molecular signatures related to EMT, metabolic reprogramming, and immune suppression, which provide transcriptional clues to the unfavorable clinical outcomes of Luminal B tumors. Moreover, we detected prominent spatial heterogeneity within the Luminal subsets 4–7 (e.g., Luminal 4 accounts for 26% of T-region cells vs. 51% of Vacuoles-region cells). Such heterogeneity may partially account for the wide prognostic variability observed among patients classified identically via bulk transcriptomics, though independent clinical cohorts are required to further verify this hypothesis.

**Single-cell RNA-seq (scRNA-seq) studies:** Prior scRNA-seq work identified three malignant Luminal subsets (Type 1–3) and proposed that Type 2 cells possess plastic properties and serve as tumor-initiating cells [27]. Our study suggests that Luminal 2 corresponds to the Type 2 cell population reported by Ma et al. This subset is localized to the PIN, a precancerous microregion, and acts as a central hub mediating intercellular communication. In addition, Type 3 and neuroendocrine-transformed cells match Luminal 4 and Luminal 7 in our dataset, respectively. Conventional scRNA-seq dissociates intact tissues and loses native spatial positional information. By integrating functional features of Luminal subsets with their in situ pathological localization, our analysis further reveals that Luminal 6, which forms nested aggregates within T regions, correlates with pathological hallmarks of highly aggressive cribriform PCa.

**TME- and CRPC-focused studies:** Previous research integrating single-cell and bulk transcriptomic datasets has constructed a refined Luminal classification system, with a primary focus on CRPC-enriched subsets and their crosstalk with specific Fibroblast populations [28]. Our study systematically extends the landscape of Luminal–Fibroblast intercellular communication. Our analyses reveal that COLLAGEN, FN1, and LAMININ ligand–receptor axes mediate pathological microregion-specific cellular communication: Fibroblast 0 predominantly interacted with Luminal 0–2 that dominate in BPH regions, while signaling between Fibroblast 2/4 and Luminal 4/7 constituted the core communication circuit in the T/Vacuoles regions. Such dynamic remodeling of the communication network highlighted the coevolution of the TME and Luminal subsets during progression of PCa. Additionally, Luminal 7 in our dataset harbored transcriptional signatures linked to CRPC, consistent with previously published reports by Ding et al. Nevertheless, our cohort lacked clinically confirmed CRPC specimens, so we cannot directly validate that Luminal 7 functionally contributes to the emergence of castration resistance. We additionally observed suppressed AR signaling accompanied by compensatory activation of the TGF-β and KRAS pathway across Luminal subsets 0, 1, 3, and 7, implying that multiple Luminal subsets might collectively participate in driving castration-resistant phenotypes in PCa.

## 5. Limitations

This study has four main limitations. First, all conclusions of the present work rely solely on observational bioinformatic analyses based on CosMx SMI, including spatial single-cell transcriptome sequencing, pathway enrichment, pseudotime trajectory inference, and computational prediction of cell–cell communication. No in vitro cellular functional assays, organoid models, or in vivo animal experiments were performed. Transcriptomics profiling can only reflect molecular correlations rather than causal regulatory relationships. Second, all specimens enrolled in this cohort were treatment-naive primary PCa tissues with no clinically confirmed CRPC samples included, so enrichment of pathways associated with castration resistance was merely identified via reference gene set databases and could not be verified within our own sample set. Third, the sample size of this study is relatively small. PCa exhibits extremely high inter-patient heterogeneity, and the limited number of patients prevents us from conducting patient-level stratified analysis, which would otherwise introduce substantial statistical bias and unreliable results. Such inter-individual variation may interfere with the accurate identification and stable typing of Luminal and Fibroblast subsets. The distribution patterns, transcriptional signatures, and functional features of each subset require external validation using larger independent clinical cohorts. Accordingly, the findings cannot be directly translated into clinical applications at this stage. Fourth, a major limitation of this study lies in the inherent sampling bias associated with prostate needle biopsy. Needle biopsies only capture small focal regions of lesions. Restricted by the spatial heterogeneity of prostate lesions, such specimens have limited representativeness and may lead to biased evaluation of molecular signatures. This systematic error cannot be eliminated merely by expanding the sample size.

To address these limitations, we outline several avenues for future investigation. First, we will enroll an expanded clinical cohort consisting of radical prostatectomy specimens to validate the distribution patterns of each Luminal subset and their correlations with clinicopathological parameters. Second, we aim to establish patient-derived organoid models and in vivo animal models to perform functional experiments, so as to dissect the biological functions of core DEGs such as *VEGFA* and *TMPRSS2*, as well as the oncogenic properties of Luminal 4 and Luminal 7. Third, we plan to integrate multi-omics datasets with single-cell spatial transcriptomic profiles for multi-layered analysis, to comprehensively decipher the underlying molecular mechanisms driving Luminal cell plasticity and evaluate their potential as diagnostic or therapeutic targets.

## 6. Conclusions

In conclusion, this study applies high-resolution CosMx SMI to profile Luminal subset heterogeneity and dynamic tumor–stromal crosstalk across the pathological spectrum of prostate lesions. Unlike conventional dissociation-based single-cell sequencing, which lacks native tissue spatial information, our work yields key novel spatially resolved findings: we identify eight Luminal subsets with unique pathological microregion and sequential evolutionary trajectories and analyze epithelial–Fibroblast communication mediated by COLLAGEN, FN1, and LAMININ signaling axes across diverse pathological microregions, and further reveal the potential association between Luminal subsets and local immune remodeling during malignant progression.

This study also provides suggestive translational insights for precision PCa management. First, the proportion ratio of Luminal 0–2 to Luminal 3–7 may serve as a candidate spatial signature to distinguish premalignant lesions from invasive ones and facilitate pathological risk stratification. Second, core genes such as *TMPRSS2*, *VEGFA* and *SPARC*, together with EMT and ECM pathways, represent potential therapeutic targets. For patients enriched with treatment-resistant Luminal 3 and 7 subsets, combinatorial regimens incorporating AR antagonists, PI3K/AKT inhibitors and anti-EMT agents warrant further experimental and clinical validation. Third, our data reveal an association between Luminal 4 and suppressed MHC expression as well as T cell exhaustion, which may provide preliminary molecular rationale for combining anti-PD-1 immunotherapy with stromal-targeted agents to reinstate anti-tumor immunity.

Collectively, our spatially resolved single-cell transcriptomic atlas refines the molecular subtyping framework of PCa and characterizes lesion-specific tumor–stromal communication patterns. These observations deliver preliminary molecular clues to facilitate the development of subset-oriented diagnostic biomarkers and combinatorial therapeutic regimens.

## Data Availability

The data supporting the conclusions of this article are available from the corresponding author on request.

## Supplementary Materials

The following supporting information can be downloaded at: https://www.mdpi.com/article/10.3390/biomedicines14081710/s1, Figure S1: Dimensionality reduction and in situ spatial distribution of 28 PCa cell clusters; Figure S2: Annotated cells are visualized at their native in situ positions, depicting the comprehensive spatial distribution of heterogeneous cell types across prostate lesion; Figure S3: Stacked bar graph showing the proportional distribution of each cell type across six pathological microregions in individual patients; Figure S4: Each Luminal cell is arranged in its spatial in situ position, showing the overall spatial distribution of each Luminal cell subset; Figure S5: The stacked bar graph illustrates the proportional distribution of each Luminal cell subset across six pathological microregions in individual patients; Figure S6: Each Fibroblast cell is arranged in its spatial in situ position, showing the overall spatial distribution of each Fibroblast cell subset; Figure S7: Stacked bar graph showing the proportional distribution of each Fibroblast cell subset across six pathological microregions in individual patients; Figure S8: Violin plots depicting the expression profiles of the top 4 marker genes for each Luminal subset; Figure S9: UMAP plots displaying the expression patterns of the top 4 marker genes for each Luminal subset; Figure S10: Cell/tissue enrichment and transcriptional regulator profiling of Luminal 4–7 subsets; Figure S11: Functional pathway and disease-term enrichment analyses for the Luminal 0 subset; Figure S12: Functional pathway and disease-term enrichment analyses for the Luminal 1 subset; Figure S13: Functional pathway and disease-term enrichment analyses for the Luminal 2 subset; Figure S14: Functional pathway and disease-term enrichment analyses for the Luminal 3 subset; Figure S15: Cell/tissue specificity and upstream transcriptional regulator enrichment analyses of Luminal 0–3 subsets; Figure S16: Venn diagrams showing the overlap analyses of Fibroblast marker gene sets to characterize Fibroblast identities; Figure S17: Dot plot illustrating the average expression level of marker genes in each Fibroblast subset; Figure S18: Violin and UMAP plots depicting expression distributions of the top 4 marker genes across five Fibroblast subsets (Fibroblast 0-4); Figure S19: Violin and UMAP plots depicting expression distributions of the top 4 marker genes across five Fibroblast subsets (Fibroblast 5-8); Figure S20: Functional, disease-associated and regulator enrichment analyses of the Fibroblast 0 subset; Figure S21: Functional, disease-associated and regulator enrichment analyses of the Fibroblast 1 subset; Figure S22: Functional, disease-associated and regulator enrichment analyses of the Fibroblast 2 subset; Figure S23: Functional, disease-associated and regulator enrichment analyses of the Fibroblast 3 subset; Figure S24: Functional, disease-associated and regulator enrichment analyses of the Fibroblast 4 subset; Figure S25: Functional, disease-associated and regulator enrichment analyses of the Fibroblast 5 subset; Figure S26: Functional, disease-associated and regulator enrichment analyses of the Fibroblast 6 subset; Figure S27: Functional, disease-associated and regulator enrichment analyses of the Fibroblast 7 subset; Figure S28: Functional, disease-associated and regulator enrichment analyses of the Fibroblast 8 subset; Figure S29: GSEA displaying FDR-adjusted significant enrichment of GO gene sets in eight luminal subsets; Figure S30: Heatmap illustrating the expression gradients of EMT-associated markers across eight Luminal subsets; Figure S31: Pseudotime trajectory analysis of eight Luminal subsets; Figure S32: Heatmap based on four network centrality measures of three signaling pathways, showing the relative importance of Luminal, Fibroblast, pericyte and tumor subsets as sender, receiver, mediator and influencer in five pathological microregions; Figure S33: Flow cytometry comparison of T-cell abundance and exhaustion status between paired normal and PCa tissues from Patient; Table S1: Summarizes basic clinical and pathological data for all 10 enrolled PCa patients. GS stands for Gleason Score. All patients received no prior androgen deprivation or other anti-tumor endocrine therapy before tissue collection; Table S2: Detection status of single-cell spatial transcriptomic data.
