# Supplementary material for: High-Resolution Spatial Transcriptomics Reveals Pathological Microregion-Specific Luminal Subset Plasticity and Core Signaling Networks Underlying Prostate Cancer Malignancy
Source: Biomedicines. 2026 Jul 30;14(8):1710. doi: 10.3390/biomedicines14081710 (PMC13509276; doi:10.3390/biomedicines14081710)
Supplement: Supplementary file 1 [file biomedicines-14-01710-s001.zip › biomedicines-4359414-supplementary.pdf]

High-Resolution Spatial Transcriptomics Reveals Pathological Microregion-Specific Luminal Subset Plasticity and Core Signaling Networks Underlying Prostate Cancer Malignancy

Supporting Information

Methods

1. Single-Cell Spatial Transcriptome Profiling (CosMx SMI Platform)

1.1 Platform Principle and Reagents

Spatial Molecular Imaging (SMI) is a high-resolution imaging-based spatial transcriptomics platform that enables in situ detection of RNA molecules within preserved tissue sections. The method employs sets of barcoded oligonucleotide probes that hybridize to target transcripts, followed by iterative cycles of fluorescence imaging and signal decoding. Each transcript is identified through a unique combinatorial barcode, allowing multiplexed detection of a large number of genes at subcellular spatial resolution while retaining tissue morphology. The resulting images are computationally decoded to generate spatially resolved gene expression profiles mapped to individual cells. The CosMx RNA Panel used in this project is Human 6K Discovery Panel, 6K-plex, RNA. And we used Human Universal Cell Segmentation Kit (RNA) for cell marker labeling.

1.2 Tissue Preprocessing and Fluorescent Staining

Spatial molecular profiling was performed according to the CosMx SMI Manual Slide Preparation for RNA Assays (MAN-10184) using FFPE sections. PCa tissues were formalin fixed (3.7% formalin) and paraffin embedded (FFPE) and sectioned at a 5μm thickness. The FFPE sections were retrieved at 100°C for 15 minutes in 1X Target Retrieval Solution, digested with 3 μg/ml Proteinase K in a hybridization oven at 40°C for 30 min, and incubated in 0.001% fiducials in 2×SSC-T, for 5 min at room temperature in the dark. The specific antibodies (CD298/B2M, PanCK, CD45) and DAPI were stained with the sample to visualize the cell morphology and select the field of view (FOV).

1.3 SMI Data Acquisition and Quality Control

The samples were loaded onto the NanoString CosMx SMI instrument for imaging. A total of 386 FOVs were placed on the tissue to select the regions of interest that matched the HE. Following reported probe hybridization, unbound probes

were washed away, and an imaging buffer was added to the slides prior to imaging. The photocleavable linkers on the fluorophores of the reporter probes were then released by UV illumination, followed by washing with strip wash buffer. The fluorescent barcodes were subsequently read by CosMx imager and decoded by probe identity to generate in-situ CosMx probe detection data.

Raw image processing and feature extraction were performed using an in-house SMI data processing pipeline which includes registration, feature detection, and localization. Z-stack images of nuclear (DAPI+) and surface staining were used for drawing cell boundaries on the samples. A cell segmentation pipeline using the machine learning algorithm Cellpose was performed to accurately assign transcripts to cell locations and subcellular compartments. The transcript profile of individual cells was generated by combining the target transcript location.

Following the generation of data on CosMx, Seurat (v5.0.3), Scanpy (v1.9.8) and Giotto (v4.0.5) were used for downstream analysis of the data. In brief, we first conducted quality control across multiple dimensions, following Nanostring's official documentation to filter out low-quality cells. Next, Scanpy was used to perform dimensionality reduction and clustering. Here, the Leiden algorithm was chosen for graph-based clustering. The processed data were then imported into the R for further analysis.

Supporting Figures

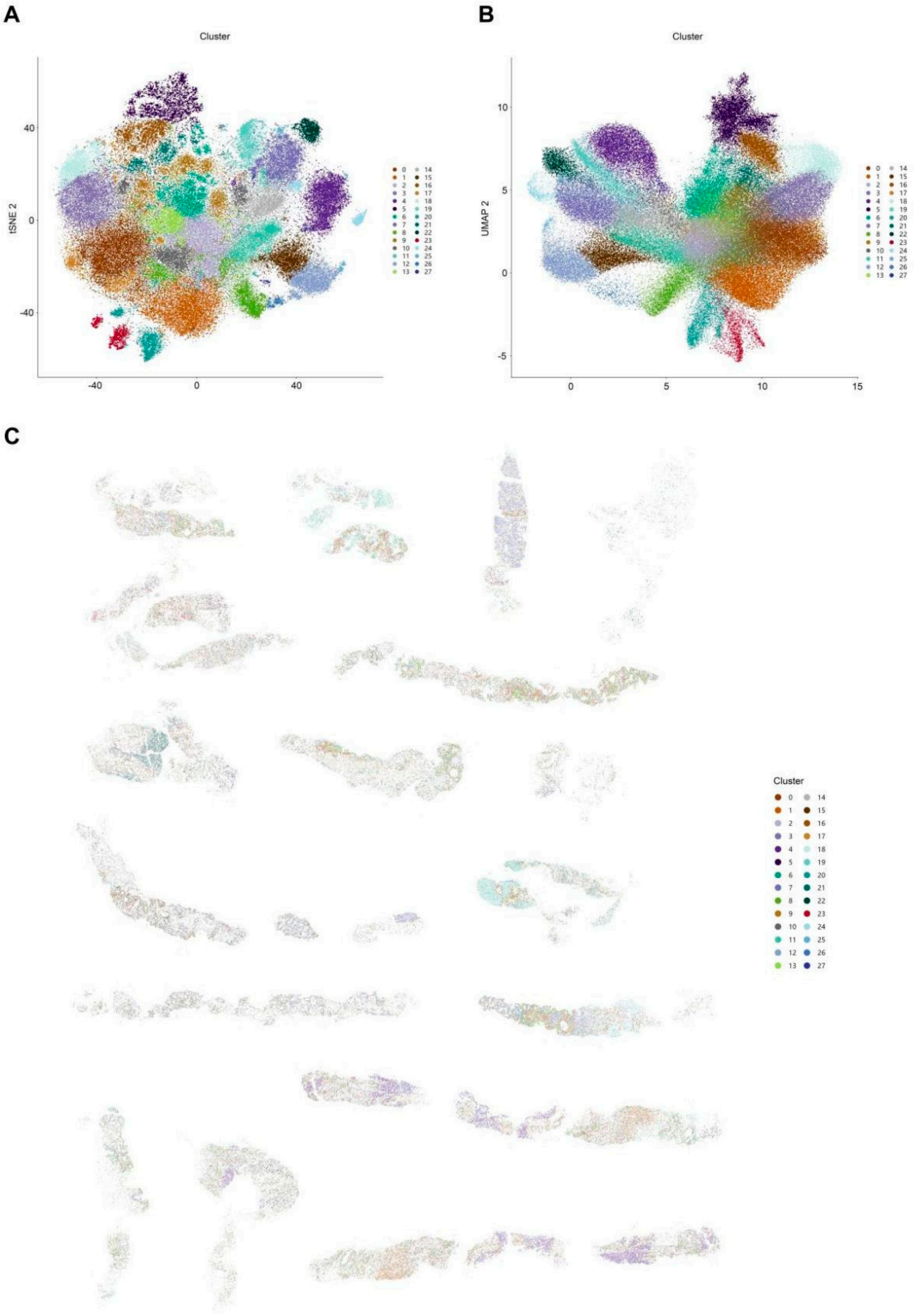

Figure S1 Dimensionality reduction and in situ spatial distribution of 28 PCa cell clusters. tSNE (A) and UMAP (B) dimensionality reduction plots depicting the two-dimensional distribution of 28 PCa cell clusters. (C) Cells are arranged in their spatial in situ positions, showing the overall spatial distribution of cell clusters across the entire tissue space.

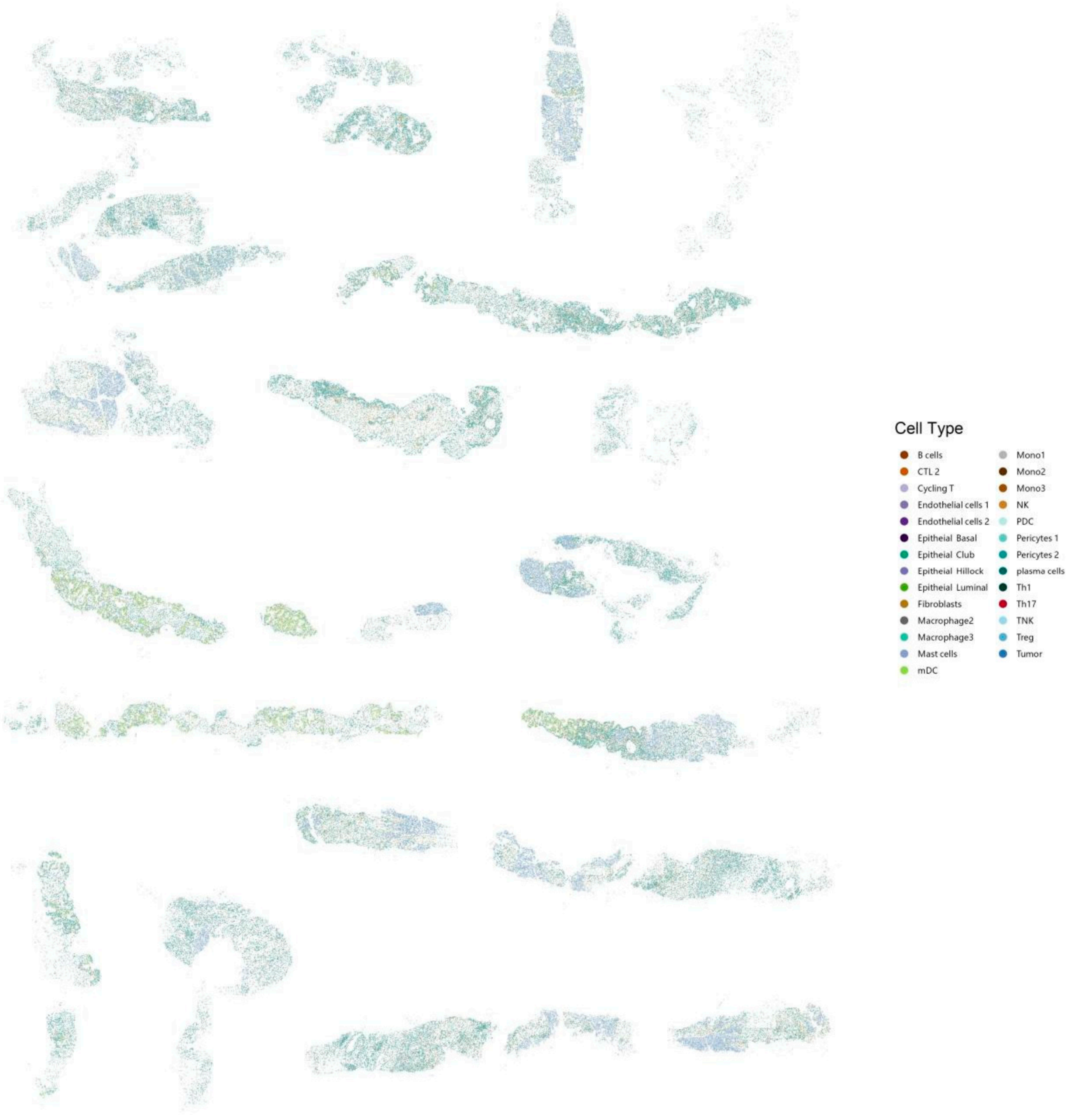

Figure S2 Annotated cells are visualized at their native in situ positions, depicting the comprehensive spatial distribution of heterogeneous cell types across prostate lesion.

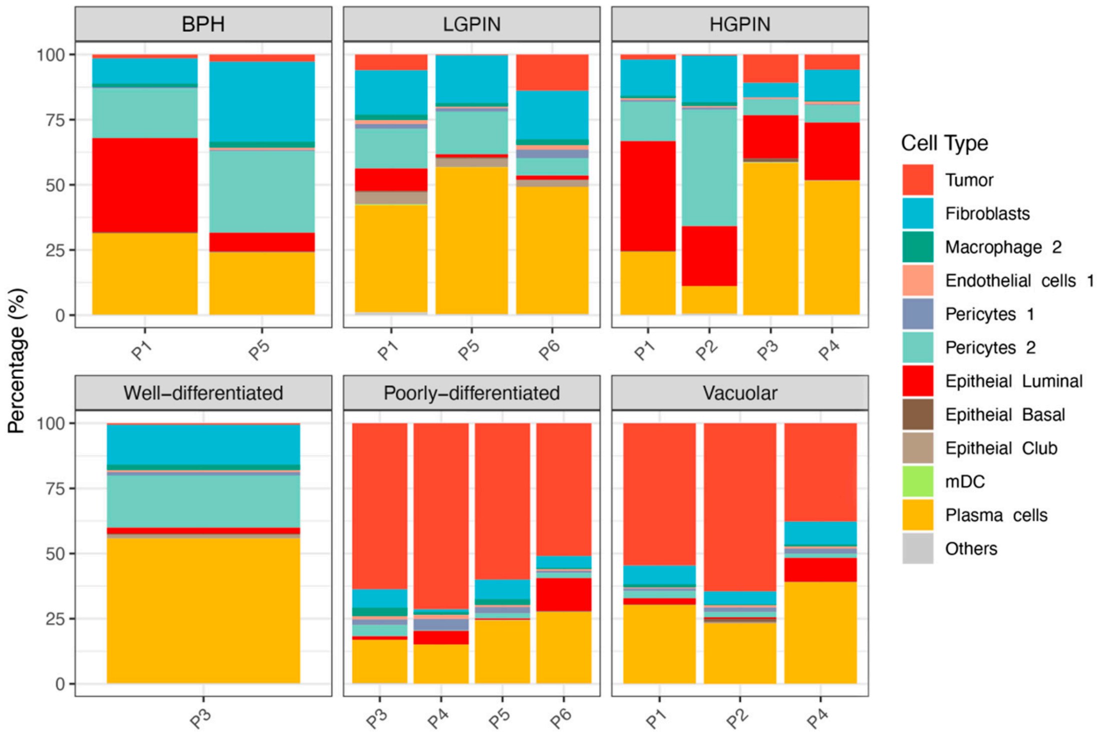

Figure S3 Stacked bar graph showing the proportional distribution of each cell type across six pathological microregions in individual patients.

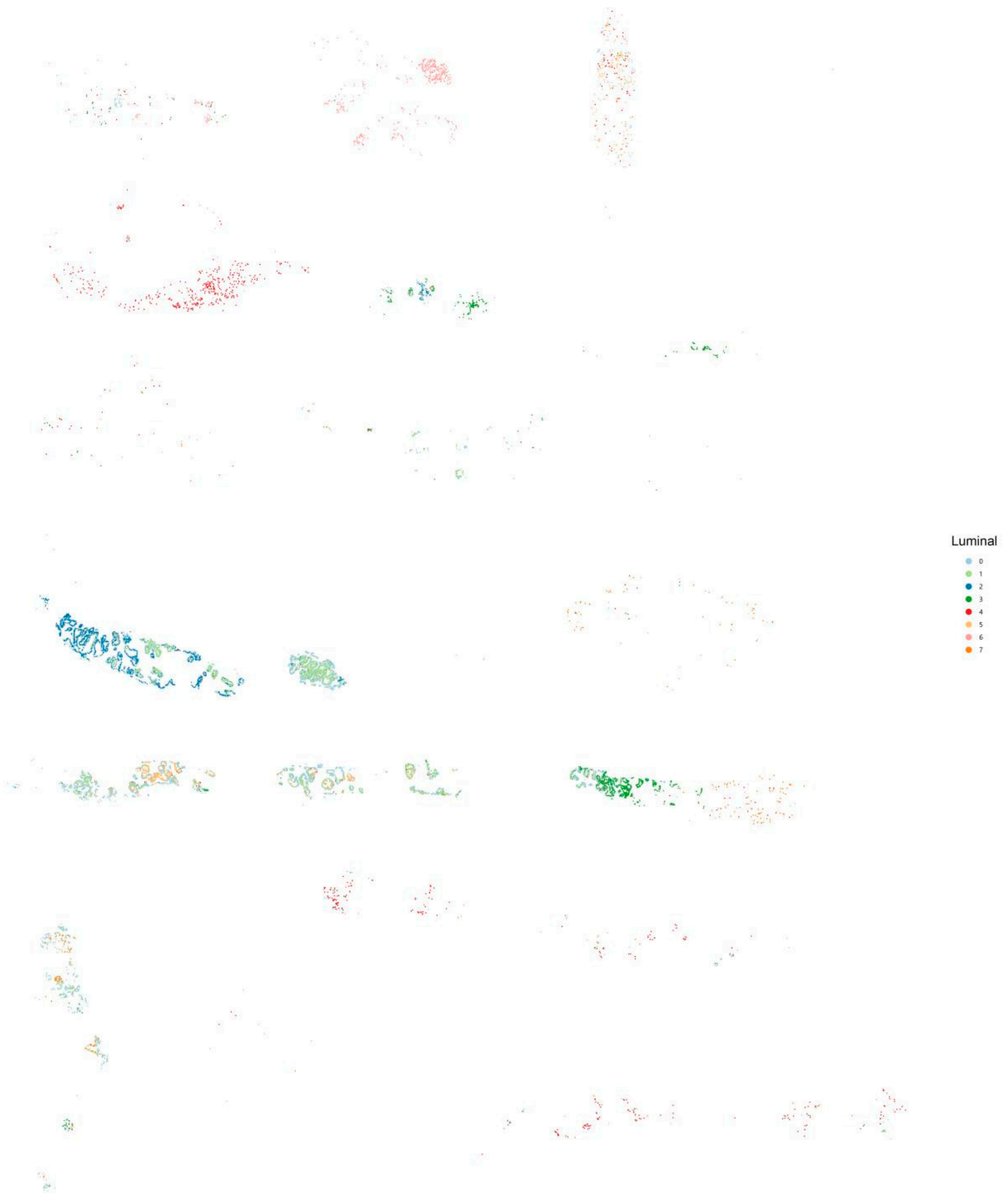

Figure S4 Each Luminal cell is arranged in its spatial in situ position, showing the overall spatial distribution of each Luminal cell subset.

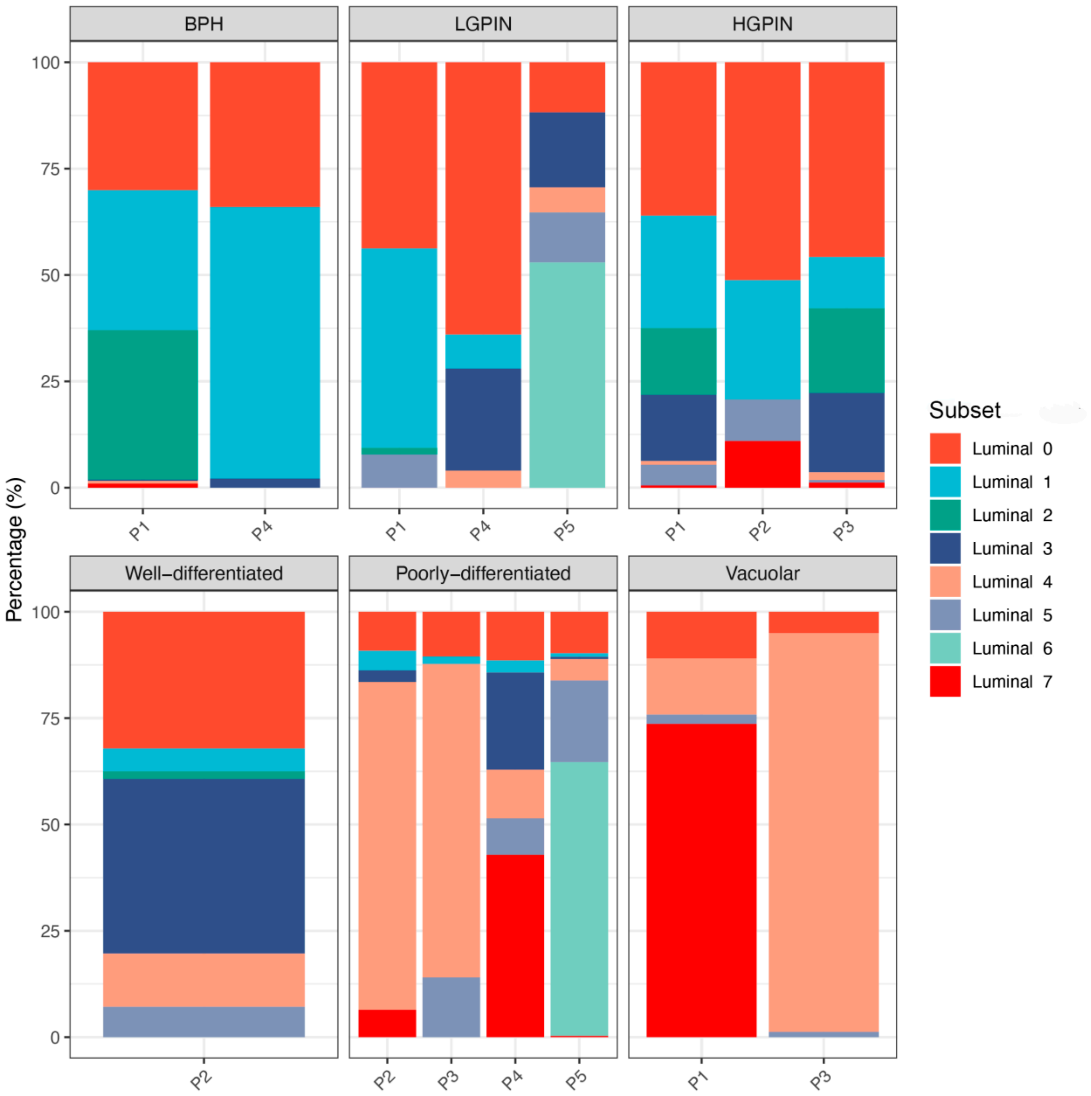

Figure S5 The stacked bar graph illustrates the proportional distribution of each Luminal cell subset across six pathological microregions in individual patients.

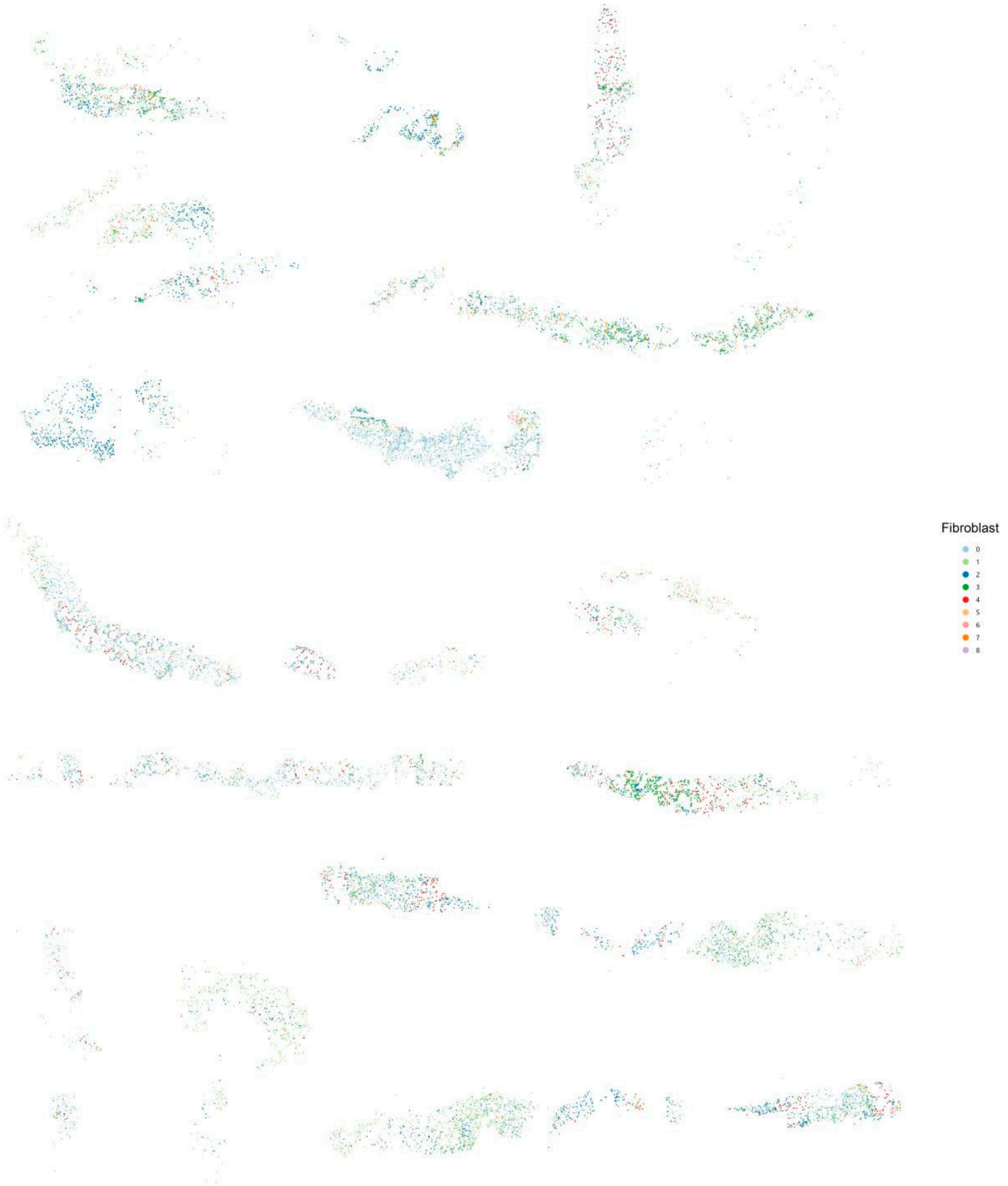

Figure S6 Each Fibroblast cell is arranged in its spatial in situ position, showing the overall spatial distribution of each Fibroblast cell subset.

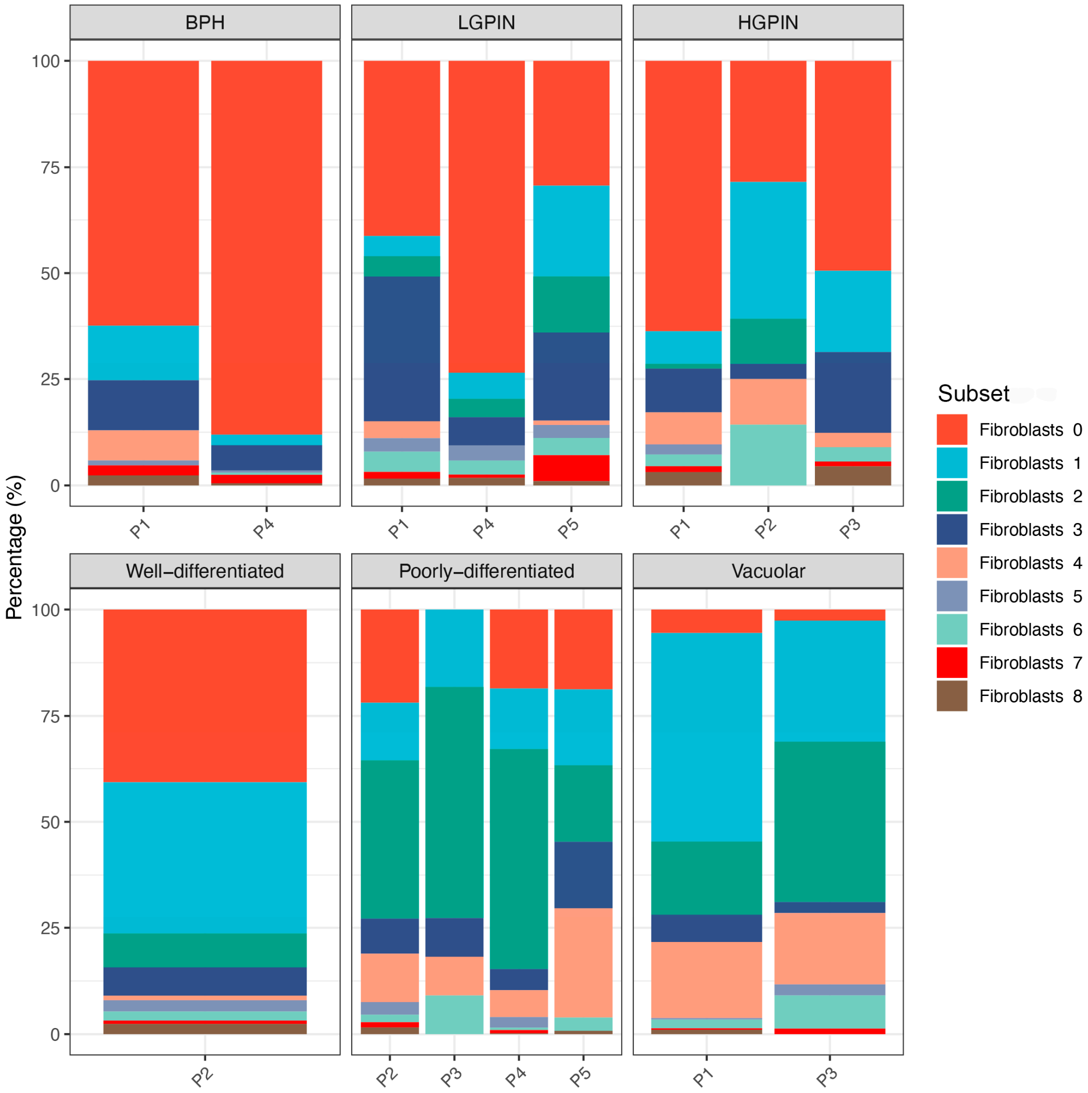

Figure S7 Stacked bar graph showing the proportional distribution of each Fibroblast cell subset across six pathological microregions in individual patients.

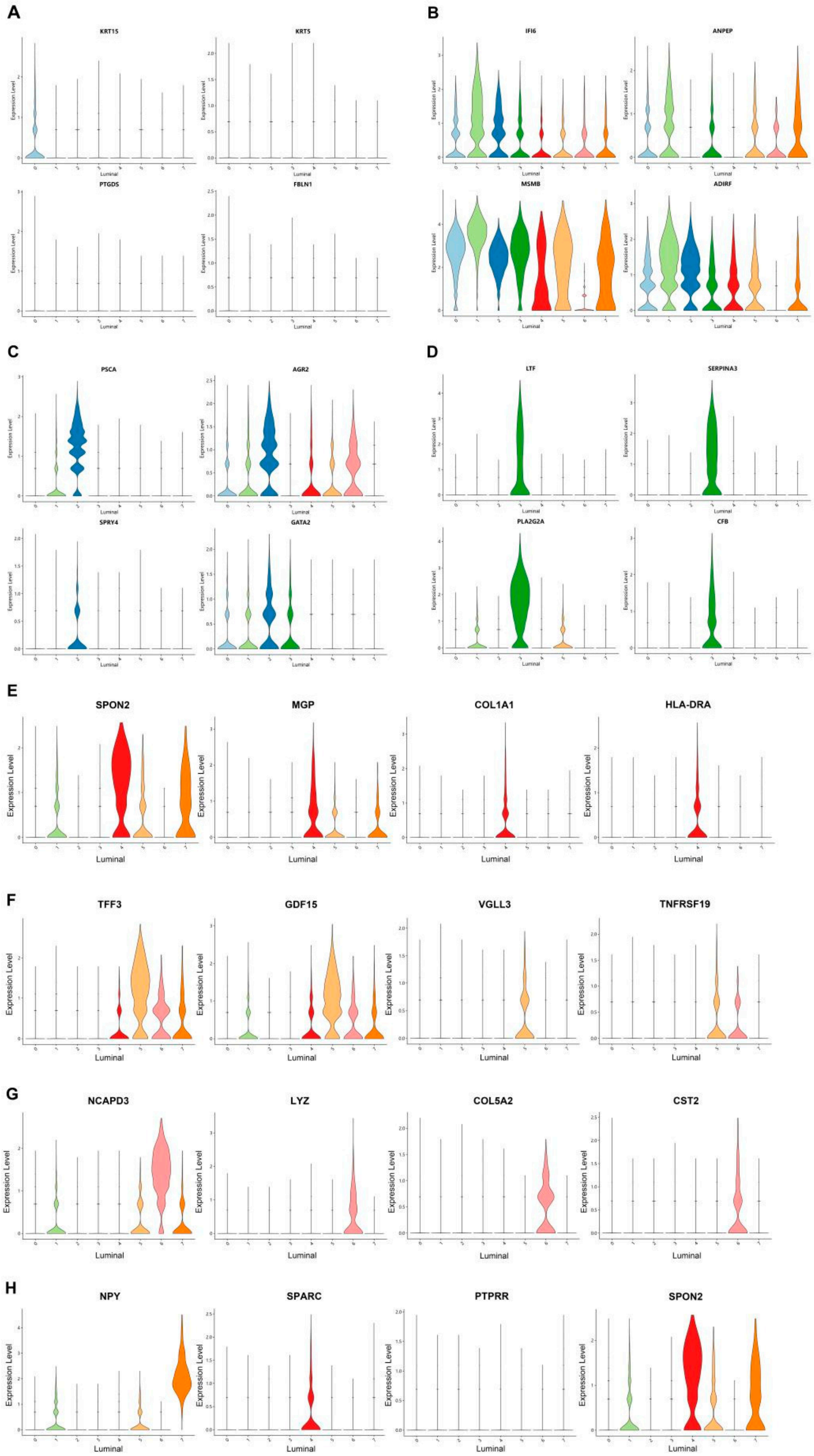

Figure S8 Violin plots depicting the expression profiles of the top 4 marker genes for each Luminal subset: Luminal 0 (A), Luminal 1 (B), Luminal 2 (C), Luminal 3 (D), Luminal 4 (E), Luminal 5 (F), Luminal 6 (G), Luminal 7 (H).

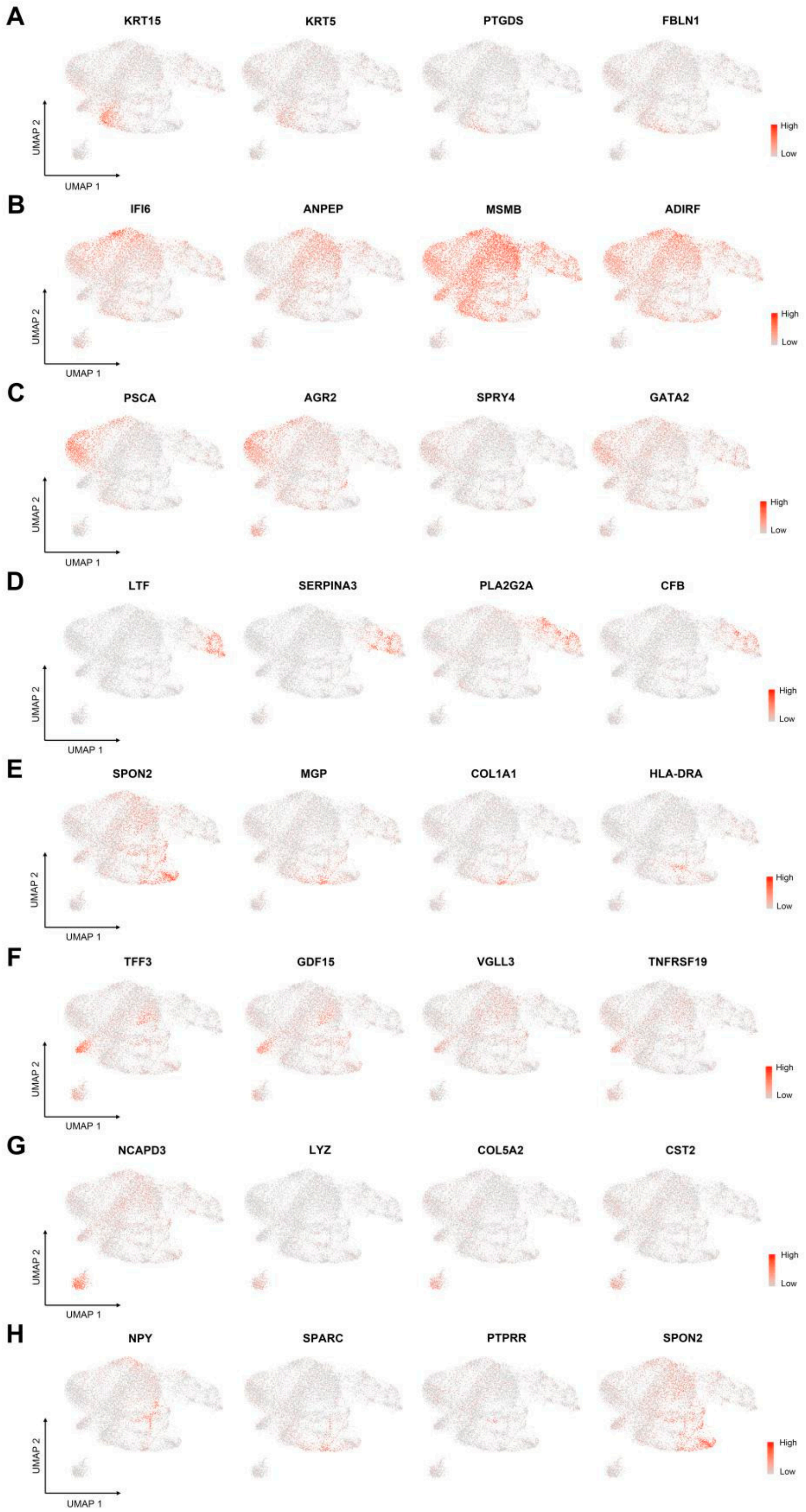

Figure S9 UMAP plots displaying the expression patterns of the top 4 marker genes for each Luminal subset: Luminal 0 (A), Luminal 1 (B), Luminal 2 (C), Luminal 3 (D), Luminal 4 (E), Luminal 5 (F), Luminal 6 (G), Luminal 7 (H).

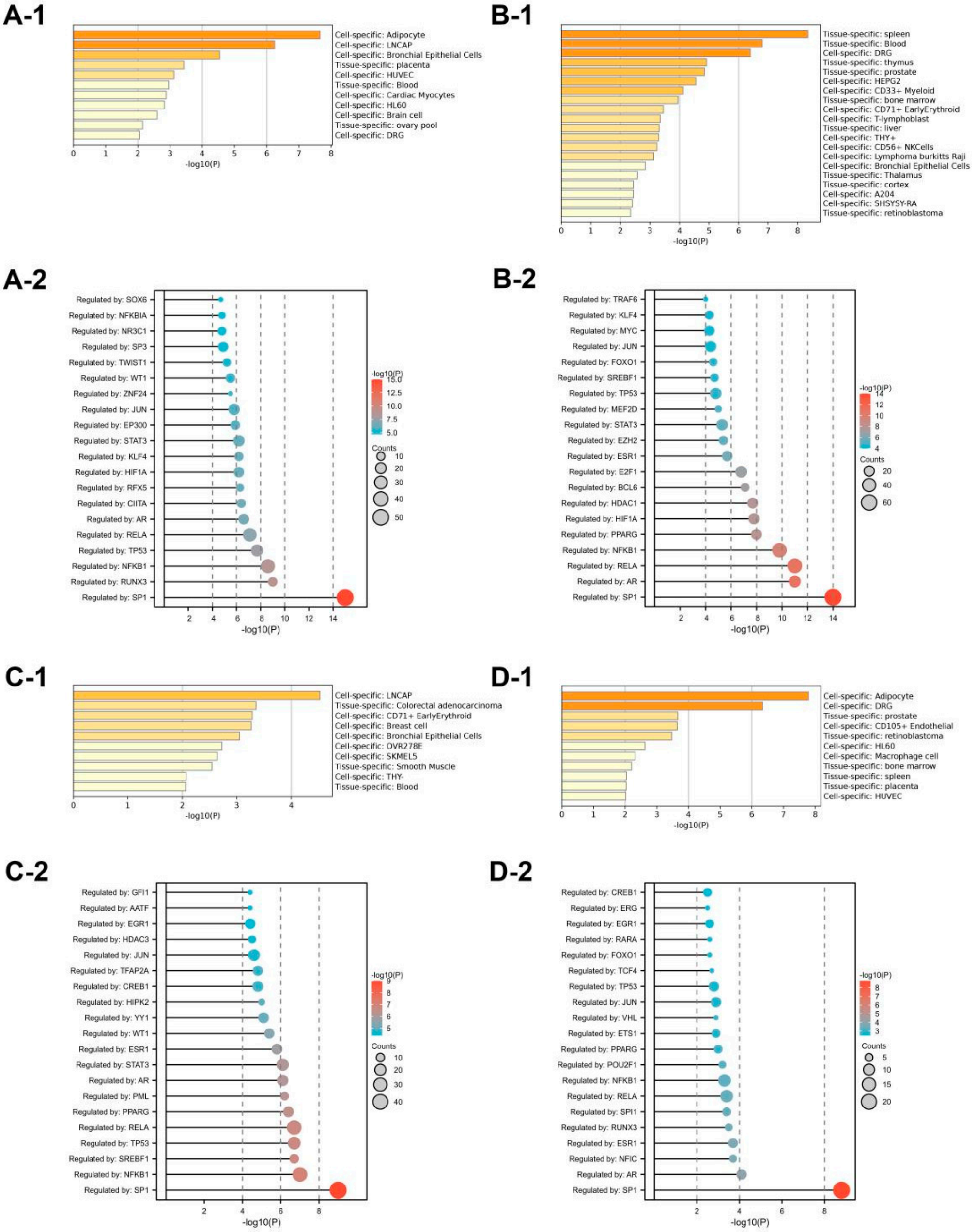

Figure S10 Cell/tissue enrichment and transcriptional regulator profiling of Luminal 4–7 subsets. (A-1 to D-1). Bar plots showing cell/tissue-specific signature enrichment for Luminal 4 (A1), Luminal 5 (B1), Luminal 6 (C1), Luminal 7 (D1) respectively. (A-2 to D-2) Lollipop chart displaying enrichment status of regulatory corresponding to the four Luminal subsets above (A-2 for Luminal 4, B-2 for Luminal 5 (B2), C2 for Luminal 6, D-2 for Luminal 7.

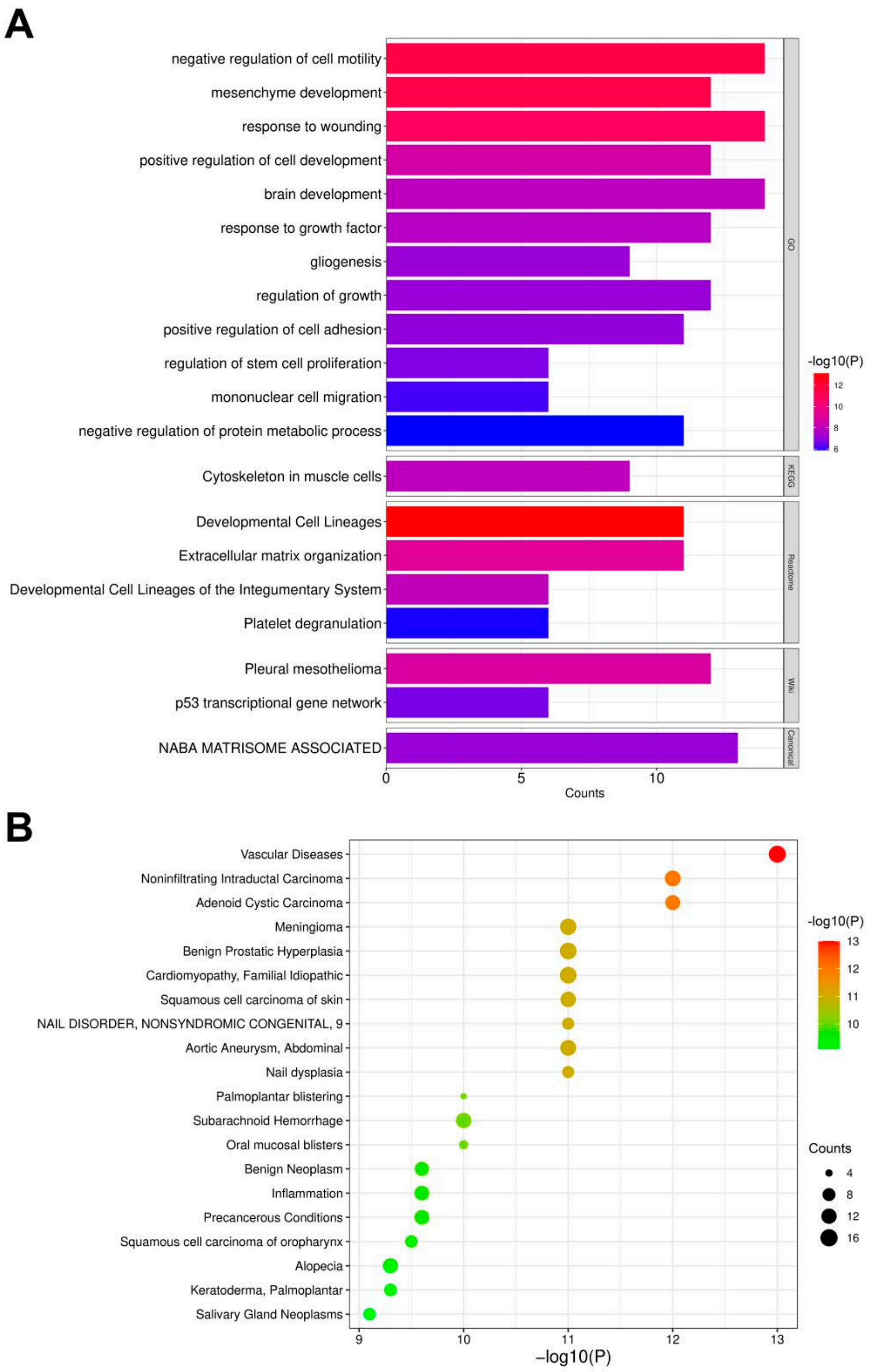

Figure S11 Functional pathway and disease-term enrichment analyses for the Luminal 0 subset. (A) Bar plot illustrating the biological functional enrichment analysis results of the Luminal 0 subset. (B) Dot plot depicting disease-related enrichment profiles of the Luminal 0 subset.

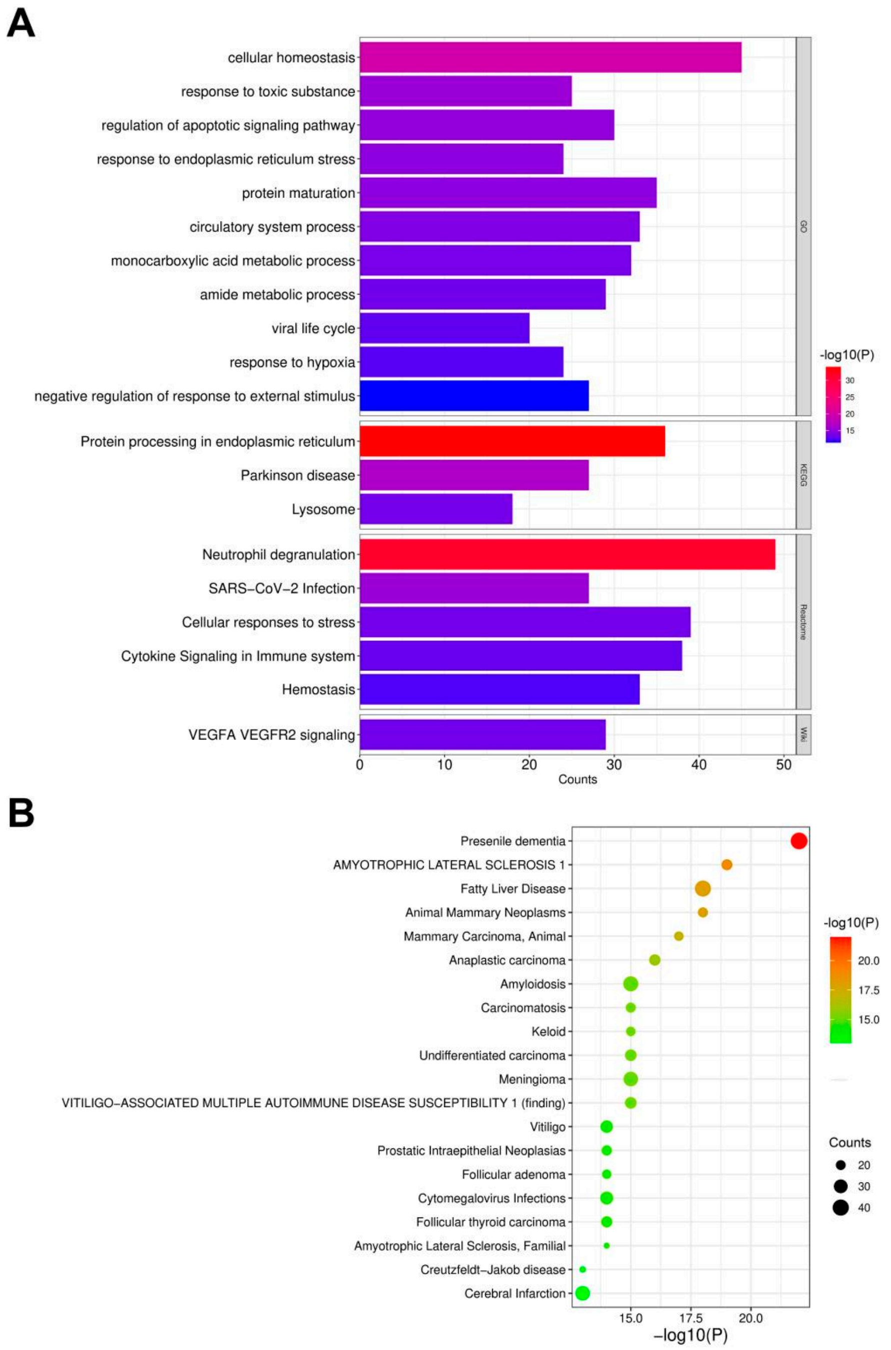

Figure S12 Functional pathway and disease-term enrichment analyses for the Luminal 1 subset. (A) Bar plot illustrating the biological functional enrichment analysis results of the Luminal 1 subset. (B) Dot plot depicting disease-related enrichment profiles of the Luminal 1 subset.

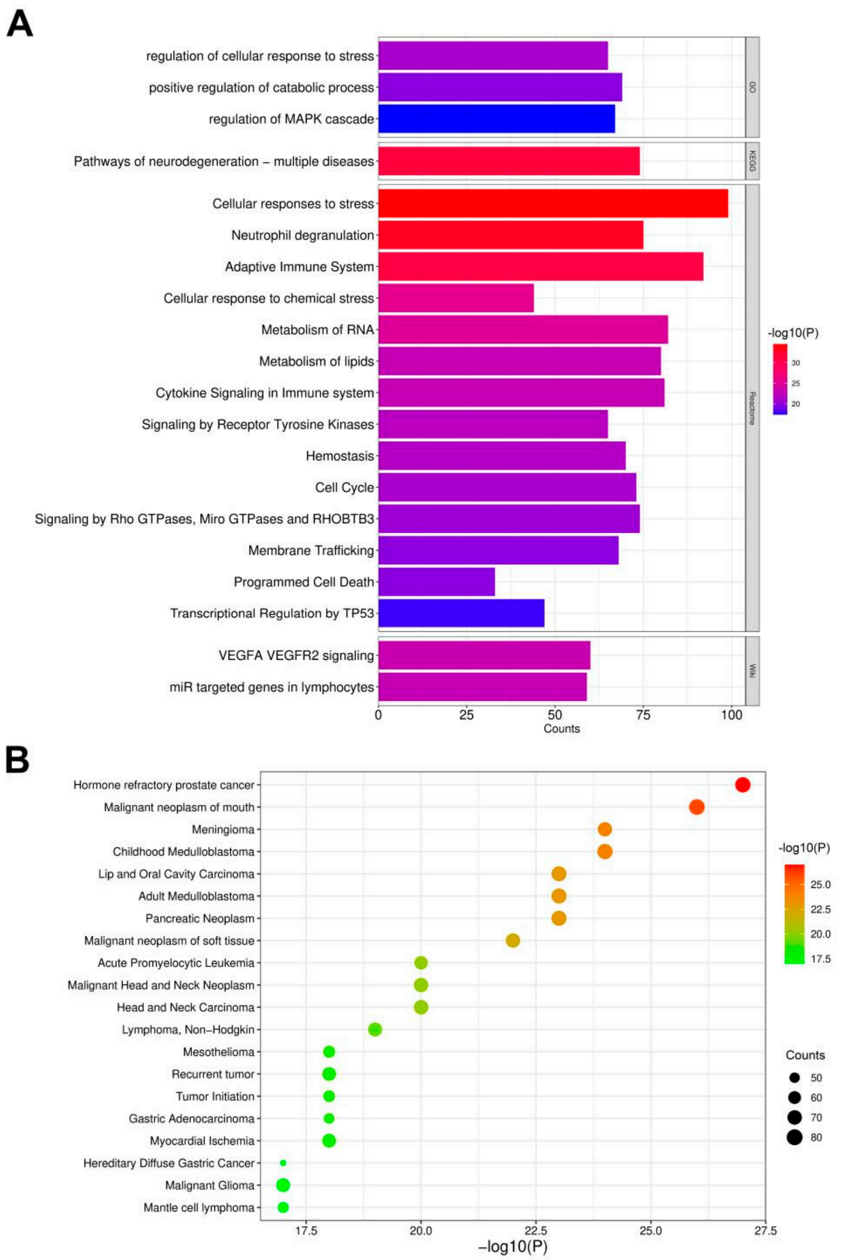

Figure S13 Functional pathway and disease-term enrichment analyses for the Luminal 2 subset. (A) Bar plot illustrating the biological functional enrichment analysis results of the Luminal 2 subset. (B) Dot plot depicting disease-related enrichment profiles of the Luminal 2 subset.

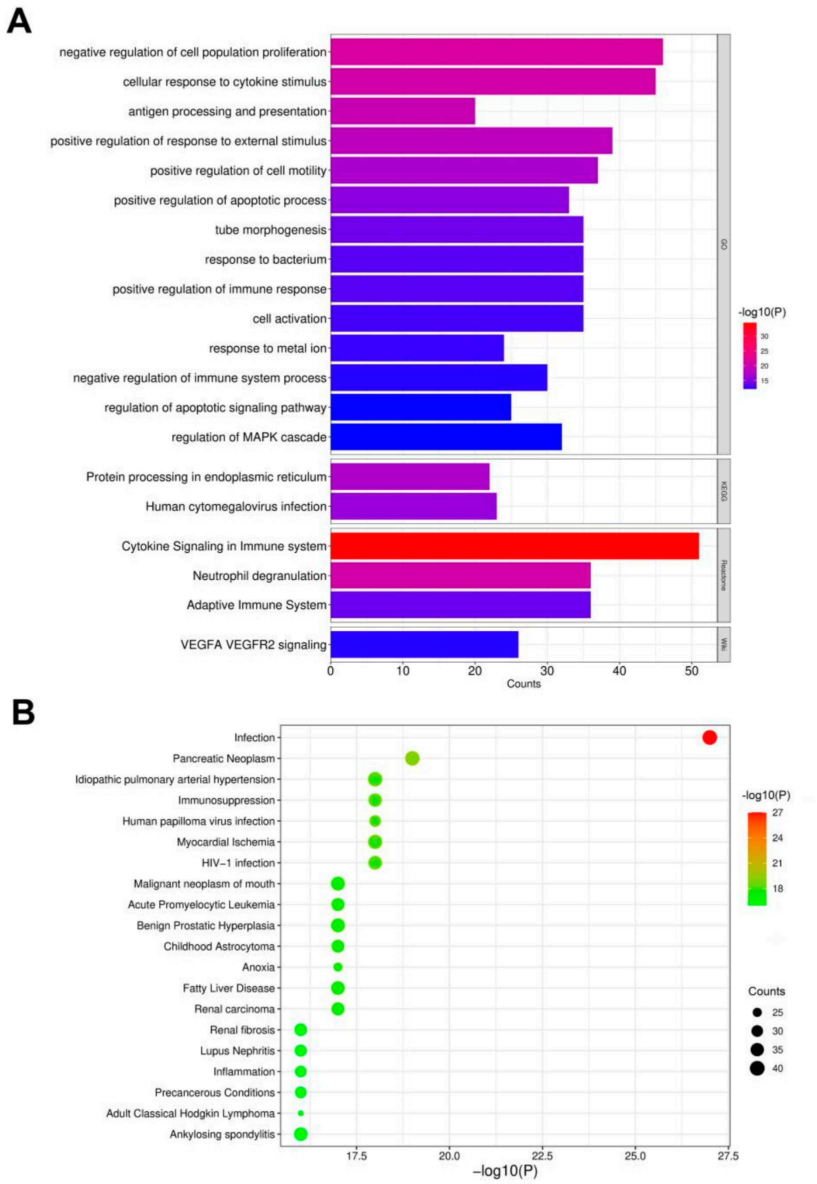

Figure S14 Functional pathway and disease-term enrichment analyses for the Luminal 3 subset. (A) Bar plot illustrating the biological functional enrichment analysis results of the Luminal 3 subset. (B) Dot plot depicting disease-related enrichment profiles of the Luminal 3 subset.

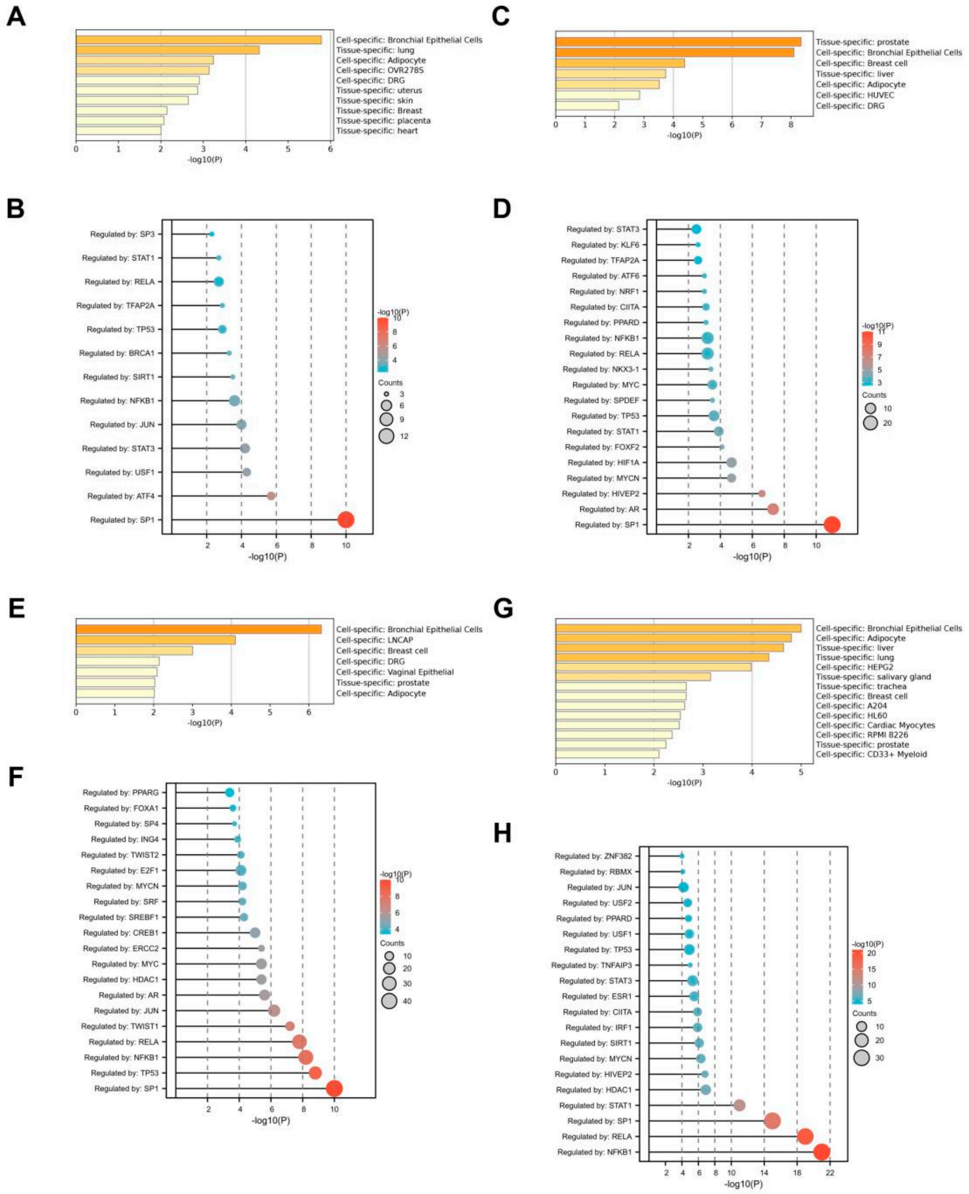

Figure S15 Cell/tissue specificity and upstream transcriptional regulator enrichment analyses of Luminal 0–3 subsets. Bar plots illustrate cell- and tissue-specific enrichment features for Luminal 0 (A), Luminal 1 (C), Luminal 2 (E), and Luminal 3 (G), respectively. Lollipop plots present the enrichment of upstream transcriptional regulators corresponding to Luminal 0 (B), Luminal 1 (D), Luminal 2 (F), and Luminal 3 (H).

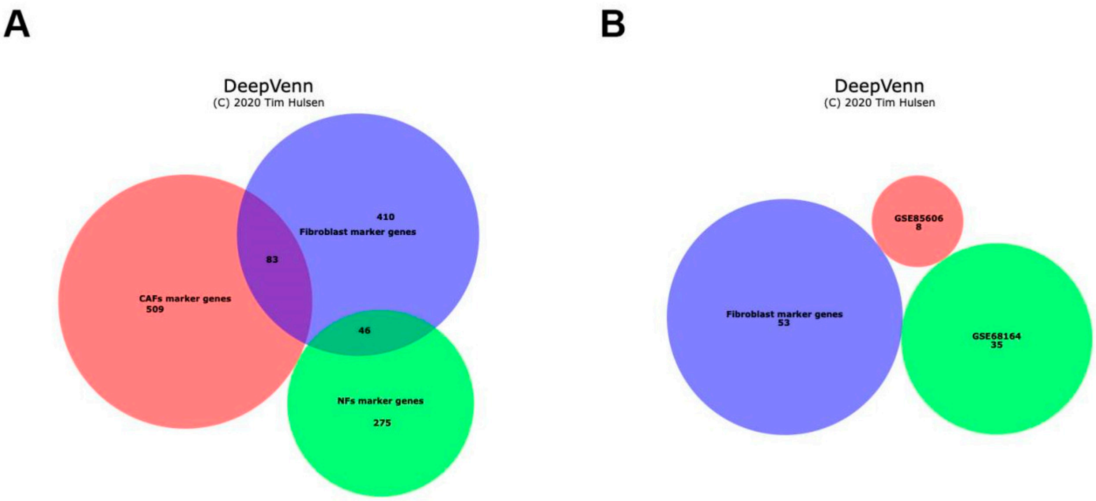

Figure S16 Venn diagrams showing the overlap analyses of Fibroblast marker gene sets to characterize Fibroblast identities. (A) Intersection analysis of publicly available pan-cancer CAF marker genes (n = 509), NF marker genes (n = 275), and Fibroblast marker genes identified in our dataset (n = 410). In total, 83 of our Fibroblast markers overlapped with canonical CAF signatures, while 46 genes overlapped with NF-specific signatures. (B) Overlap comparison between our Fibroblast marker genes from this study (n = 53) and NF signature genes extracted from two independent PCa datasets (GSE85606 and GSE68164, containing 8 and 35 NF markers respectively), with zero shared genes detected in this comparison.

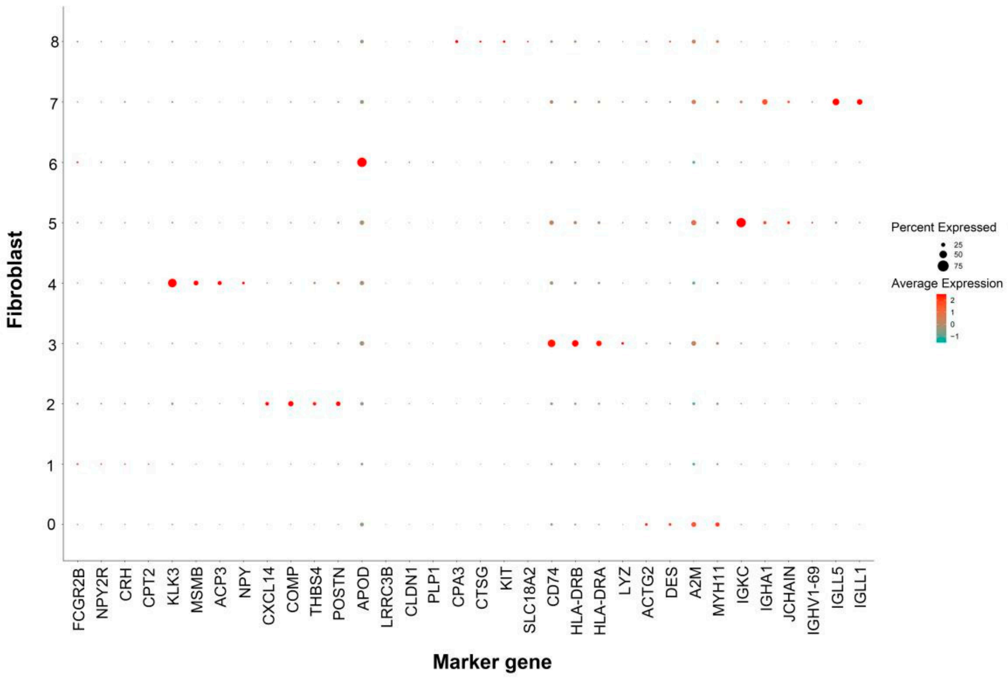

Figure S17 Dot plot illustrating the average expression level of marker genes in each Fibroblast subset.

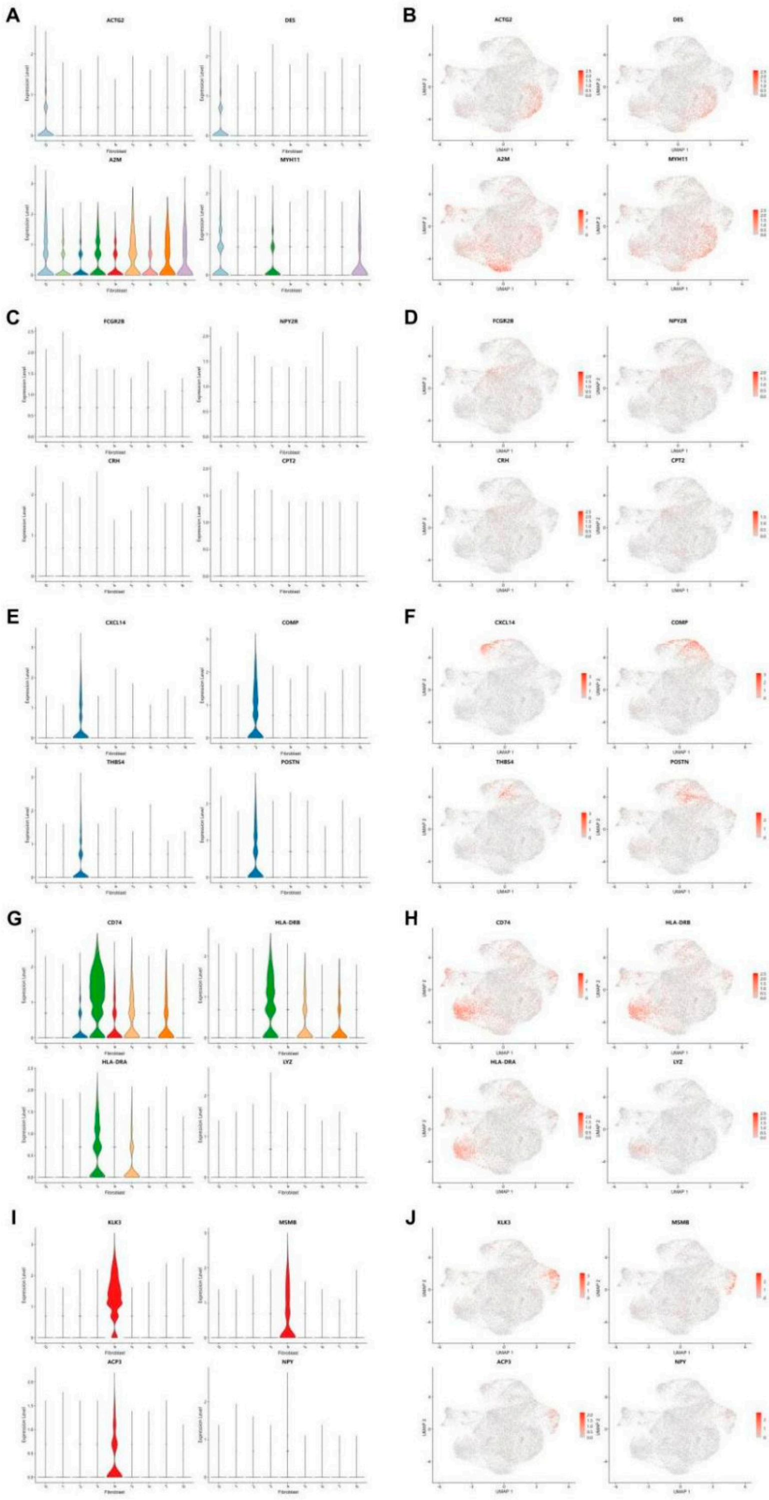

Figure S18 Violin and UMAP plots depicting expression distributions of the top 4 marker genes across five Fibroblast subsets (Fibroblast 0-4). Violin plots (A, C, E, G, I) depicted expression distributions of the top 4 marker genes expressed by Fibroblast 0, Fibroblast 1, Fibroblast 2, Fibroblast 3 and Fibroblast 4 respectively. Matching UMAP plots (B, D, F, H, J) visualized the spatial expression patterns of these marker genes across the five Fibroblast subsets.

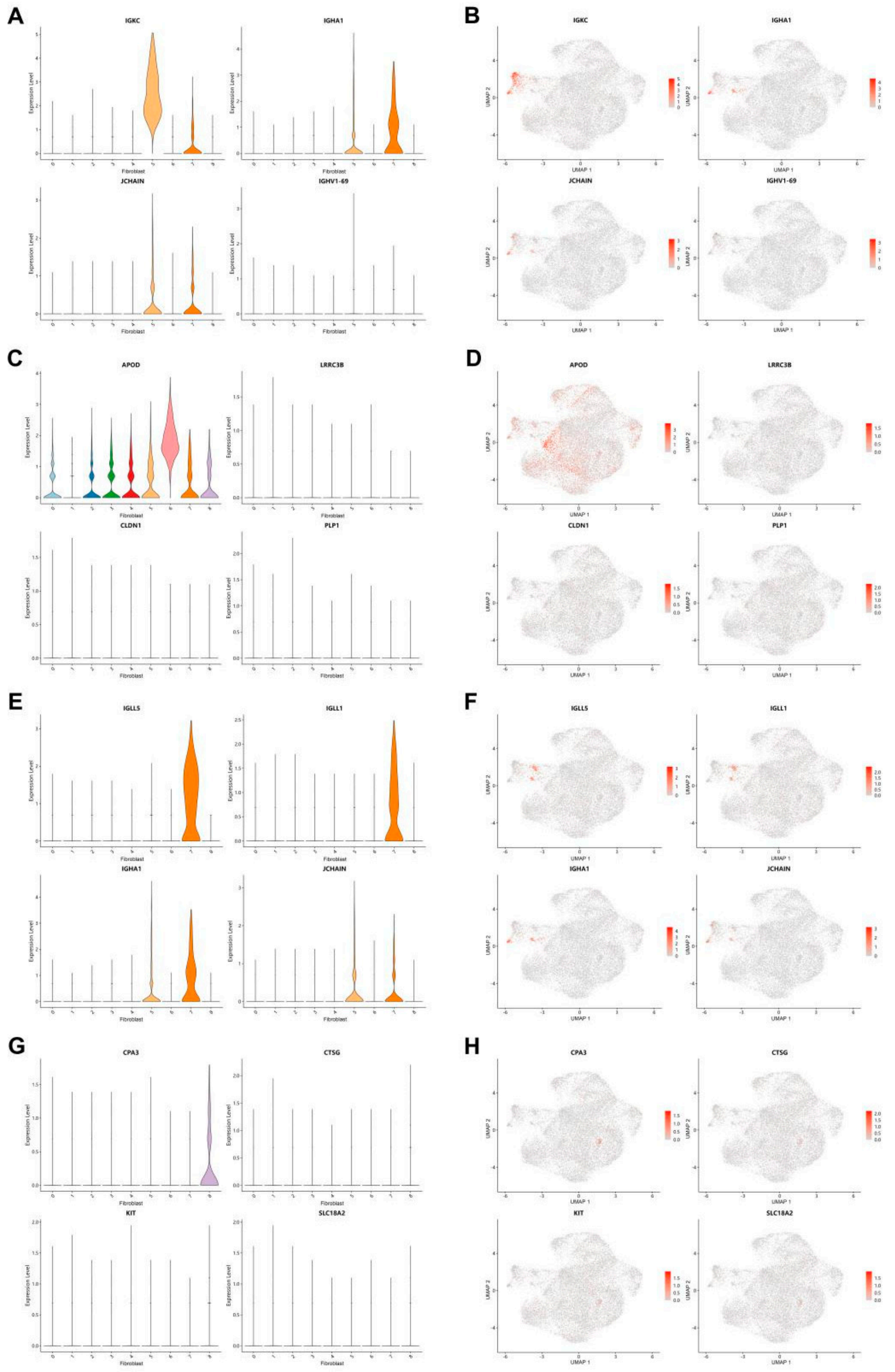

Figure S19 Violin and UMAP plots depicting expression distributions of the top 4 marker genes across five Fibroblast subsets (Fibroblast 5-8). Violin plots (A, C, E, G) depicted expression distributions of the top 4 marker genes expressed by Fibroblast 5, Fibroblast 6, Fibroblast 7 and Fibroblast 8 respectively. Matching UMAP plots (B, D, F, H) visualized the spatial expression patterns of these marker genes across the five Fibroblast subsets.

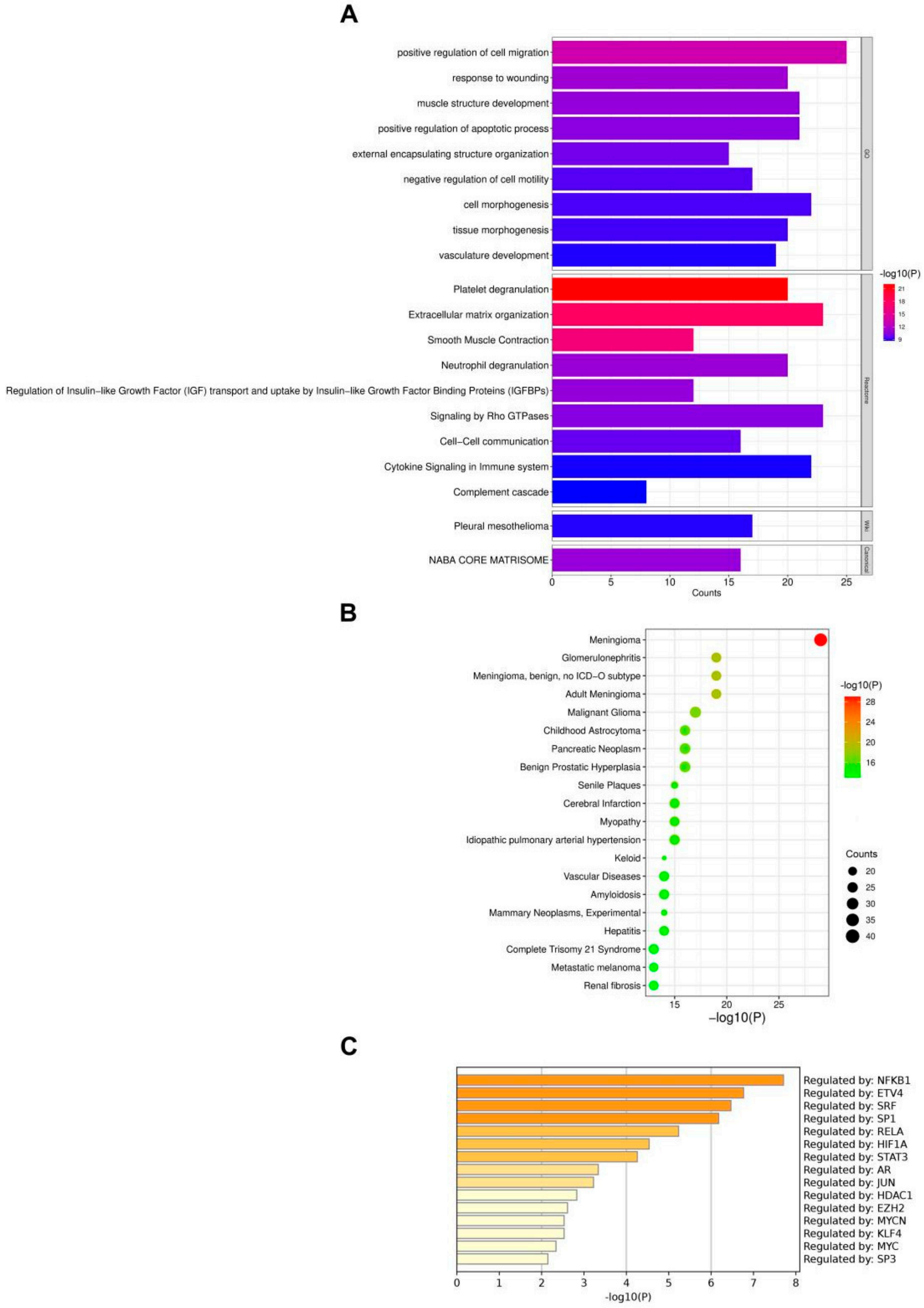

Figure S20 Functional, disease-associated and regulator enrichment analyses of the Fibroblast 0 subset. (A) Bar plot illustrating biological functional enrichment results for the Fibroblast 0 subset. (B) Dot plot depicting disease-related enrichment profiles of the Fibroblast 0 subset. (C) Bar plot displaying enrichment of transcriptional regulators associated with the Fibroblast 0 subset.

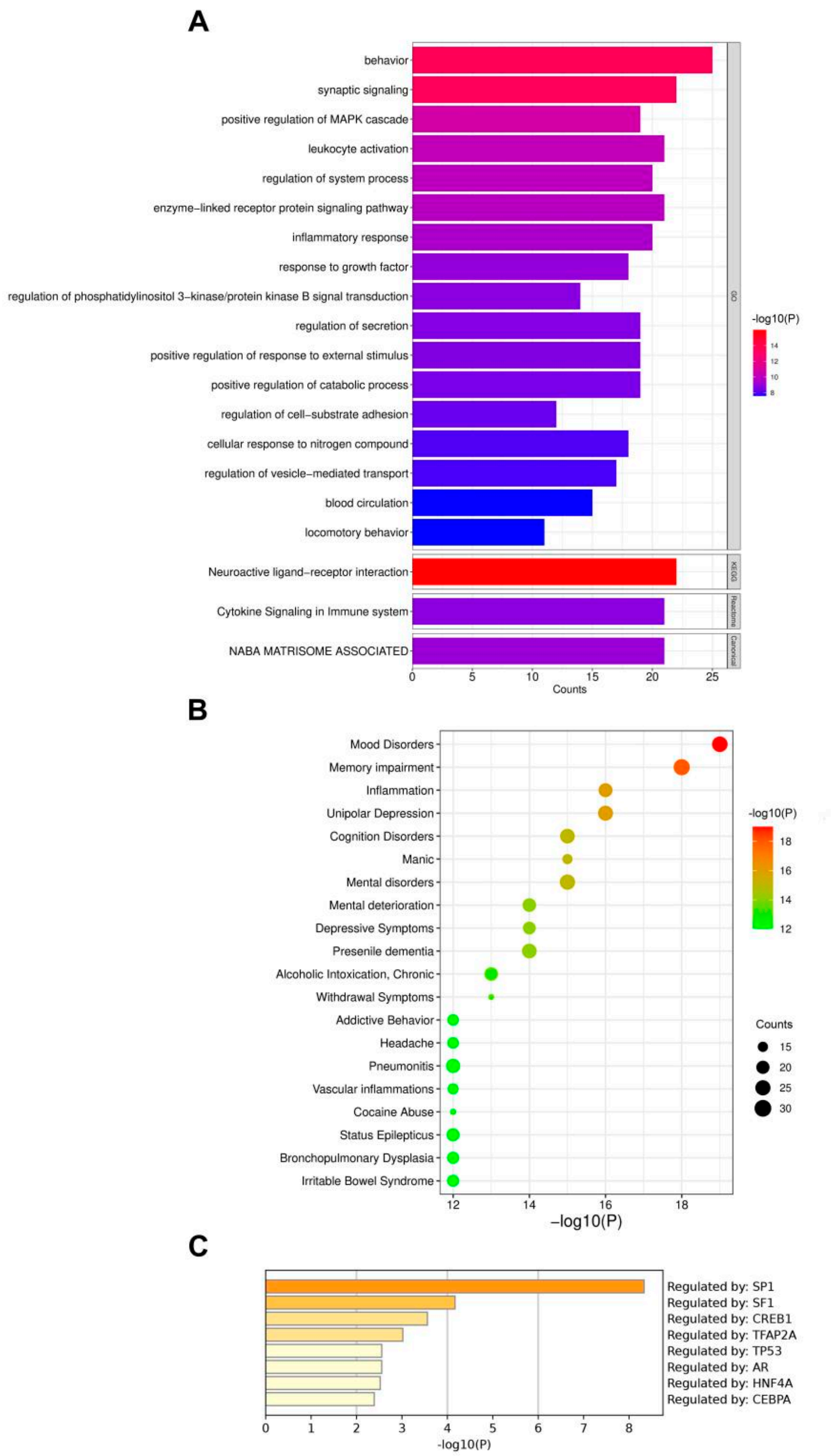

Figure S21 Functional, disease-associated and regulator enrichment analyses of the Fibroblast 1 subset. (A) Bar plot illustrating biological functional enrichment results for the Fibroblast 1 subset. (B) Dot plot depicting disease-related enrichment profiles of the Fibroblast 1 subset. (C) Bar plot displaying enrichment of transcriptional regulators associated with the Fibroblast 1 subset.

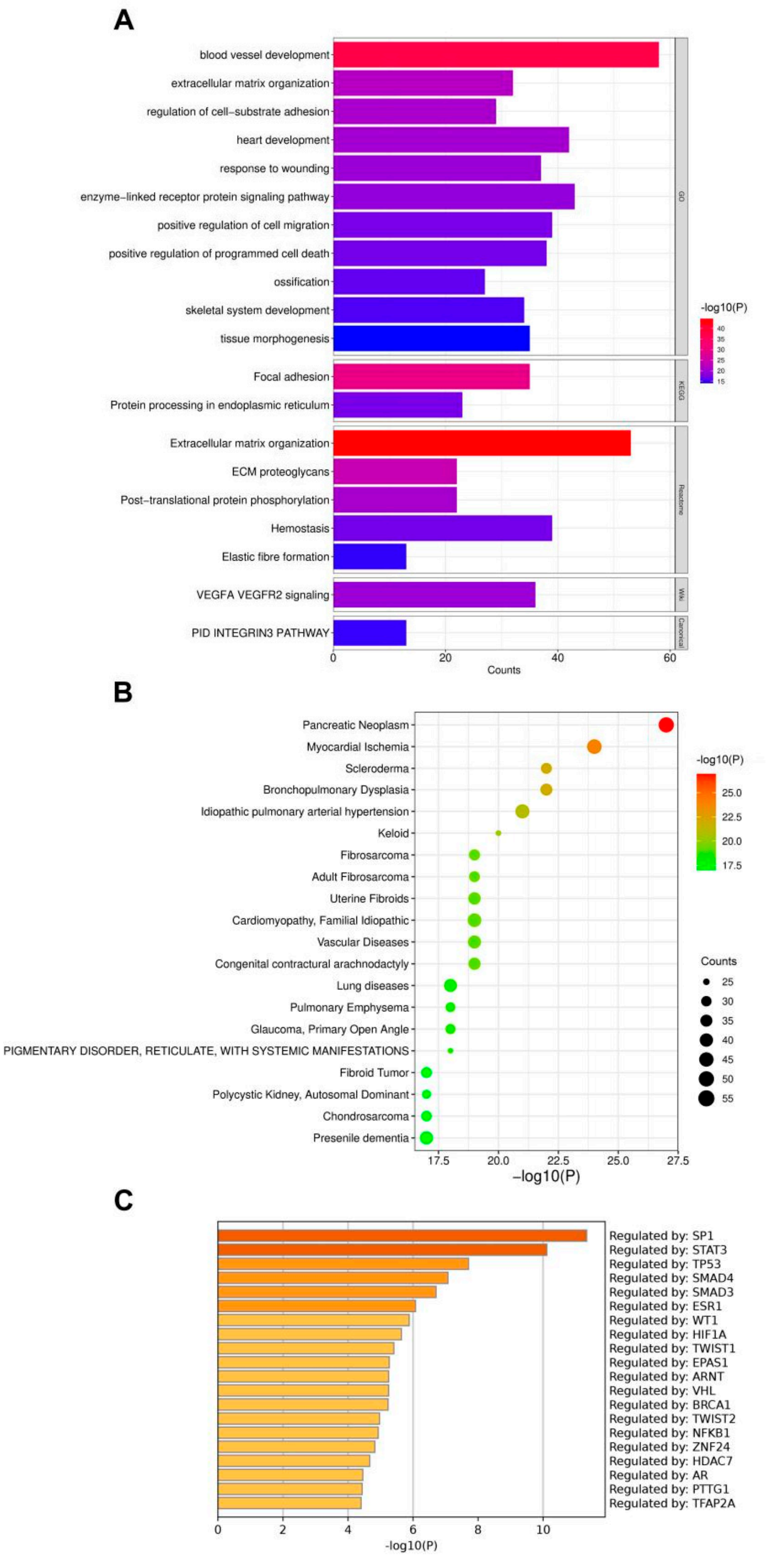

Figure S22 Functional, disease-associated and regulator enrichment analyses of the Fibroblast 2 subset. (A) Bar plot illustrating biological functional enrichment results for the Fibroblast 2 subset. (B) Dot plot depicting disease-related enrichment profiles of the Fibroblast 2 subset. (C) Bar plot displaying enrichment of transcriptional regulators associated with the Fibroblast 2 subset.

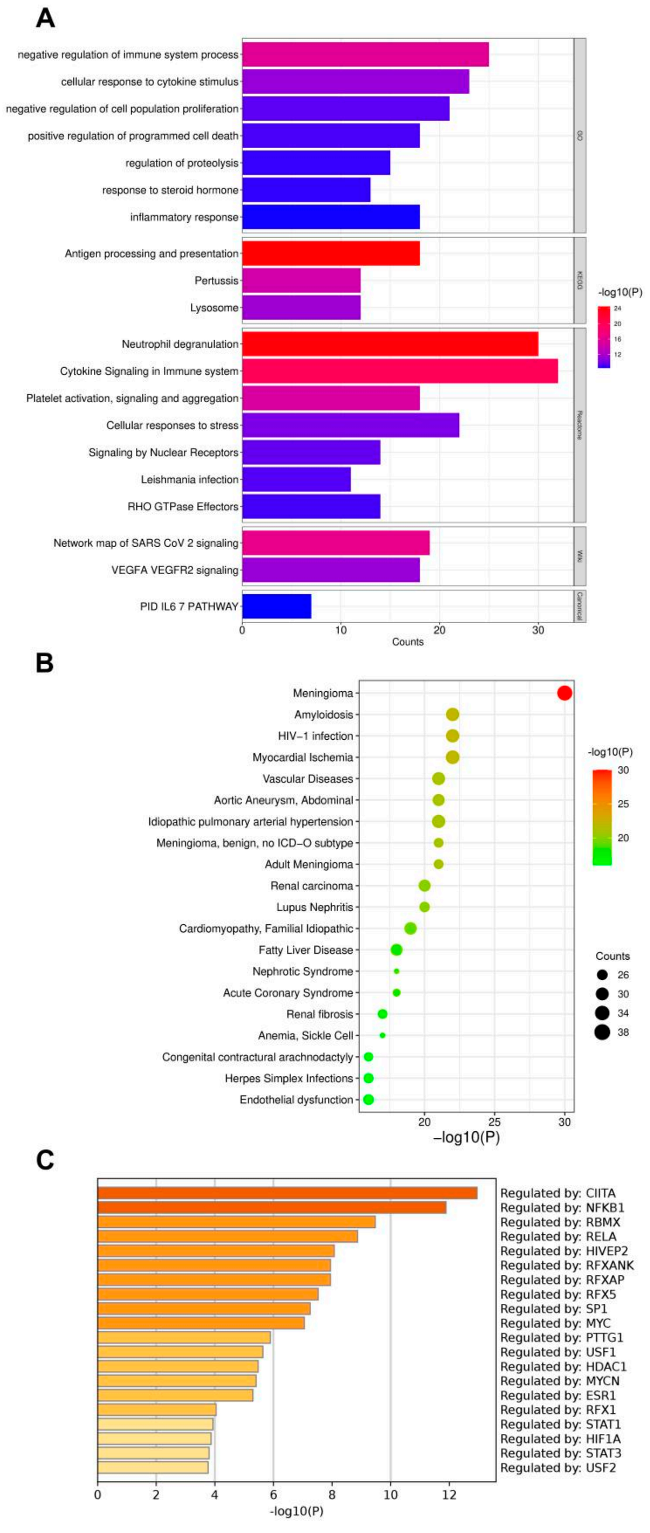

Figure S23 Functional, disease-associated and regulator enrichment analyses of the Fibroblast 3 subset. (A) Bar plot illustrating biological functional enrichment results for the Fibroblast 3 subset. (B) Dot plot depicting disease-related enrichment profiles of the Fibroblast 3 subset. (C) Bar plot displaying enrichment of transcriptional regulators associated with the Fibroblast 3 subset.

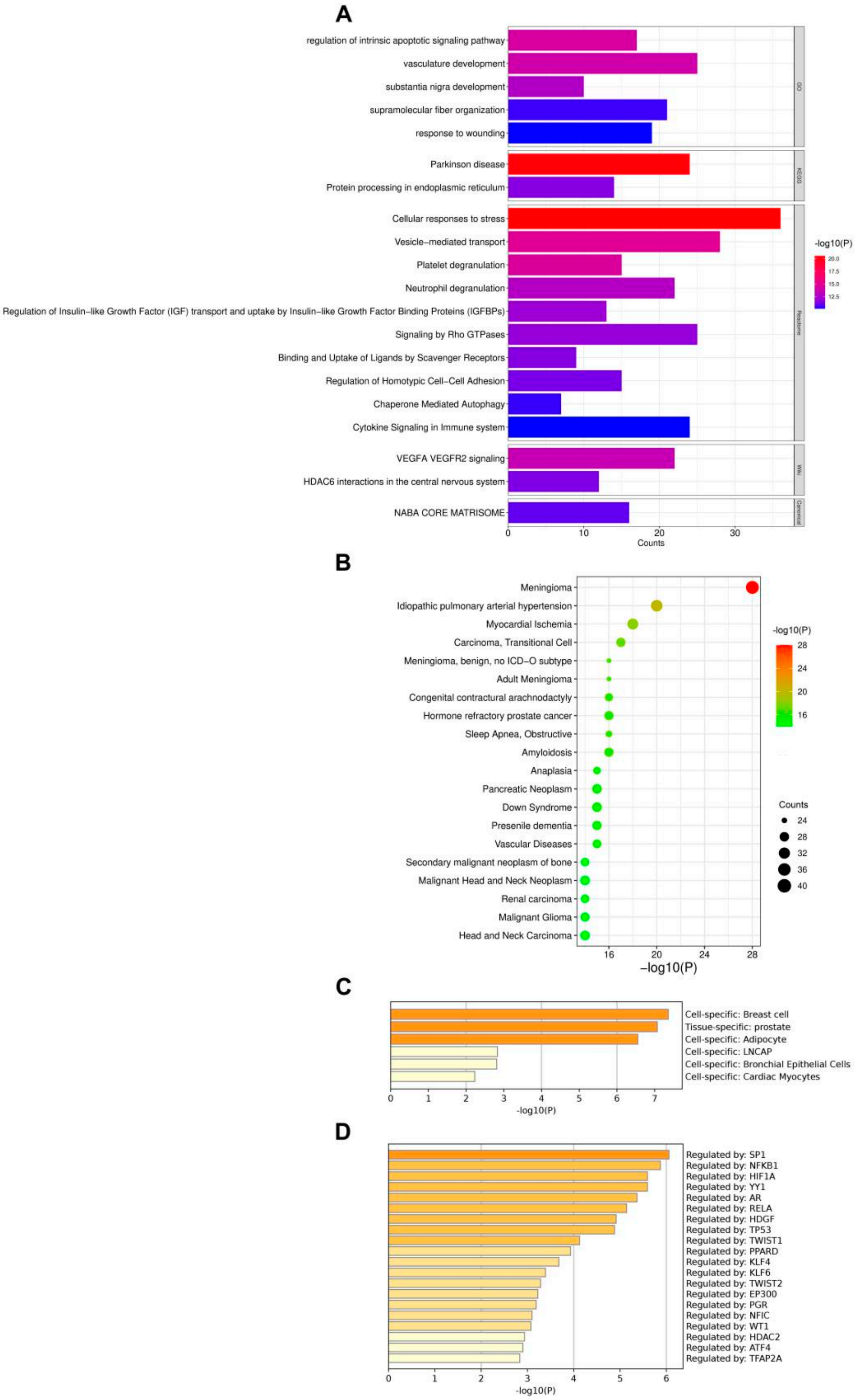

Figure S24 Functional, disease-associated and regulator enrichment analyses of the Fibroblast 4 subset. (A) Bar plot illustrating biological functional enrichment results for the Fibroblast 4 subset. (B) Dot plot depicting disease-related enrichment profiles of the Fibroblast 4 subset. (C) Bar plot showing cell/tissue-specific signature enrichment for Fibroblast 4 subset. (D) Bar plot displaying enrichment of transcriptional regulators associated with the Fibroblast 4 subset.

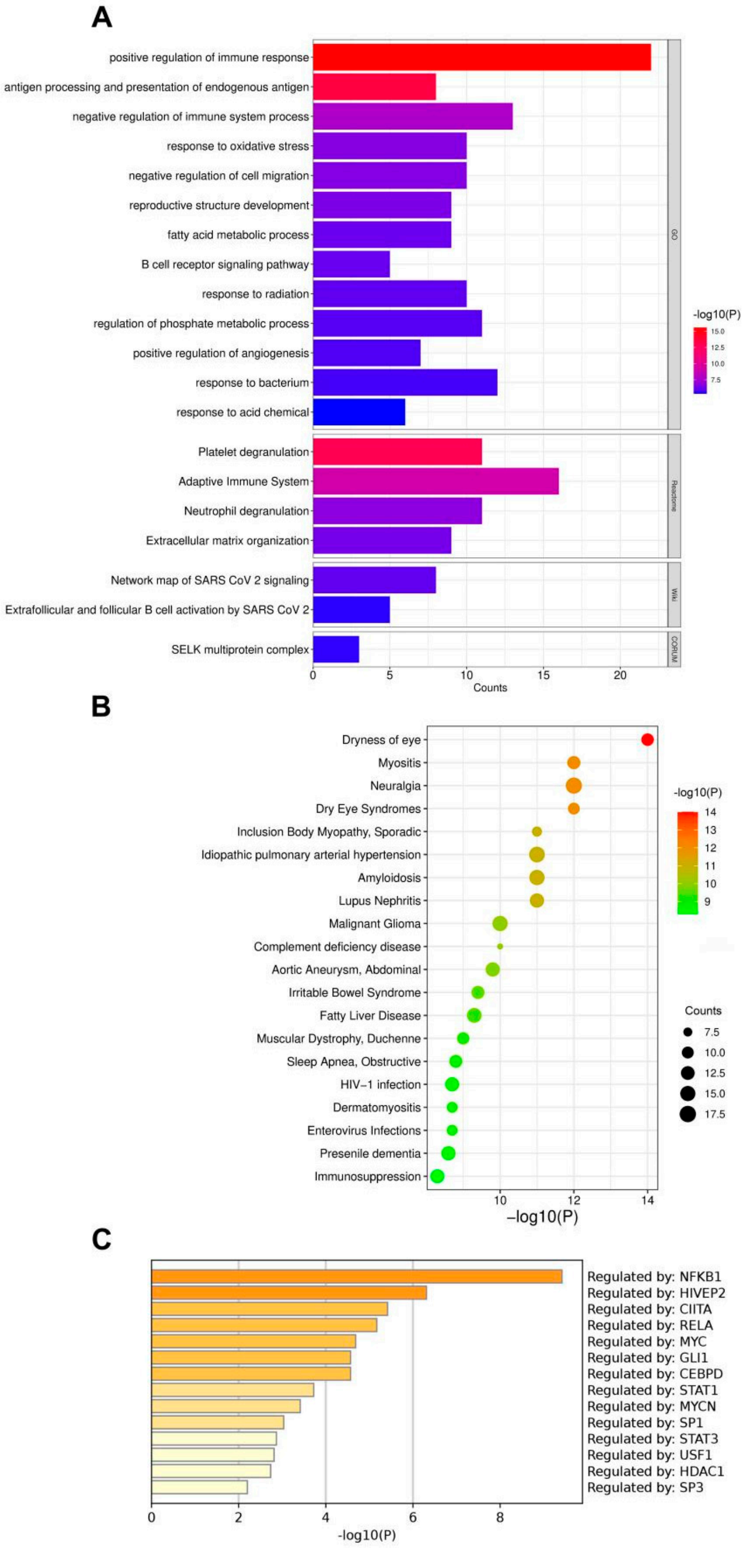

Figure S25 Functional, disease-associated and regulator enrichment analyses of the Fibroblast 5 subset. (A) Bar plot illustrating biological functional enrichment results for the Fibroblast 5 subset. (B) Dot plot depicting disease-related enrichment profiles of the Fibroblast 5 subset. (C) Bar plot displaying enrichment of transcriptional regulators associated with the Fibroblast 5 subset.

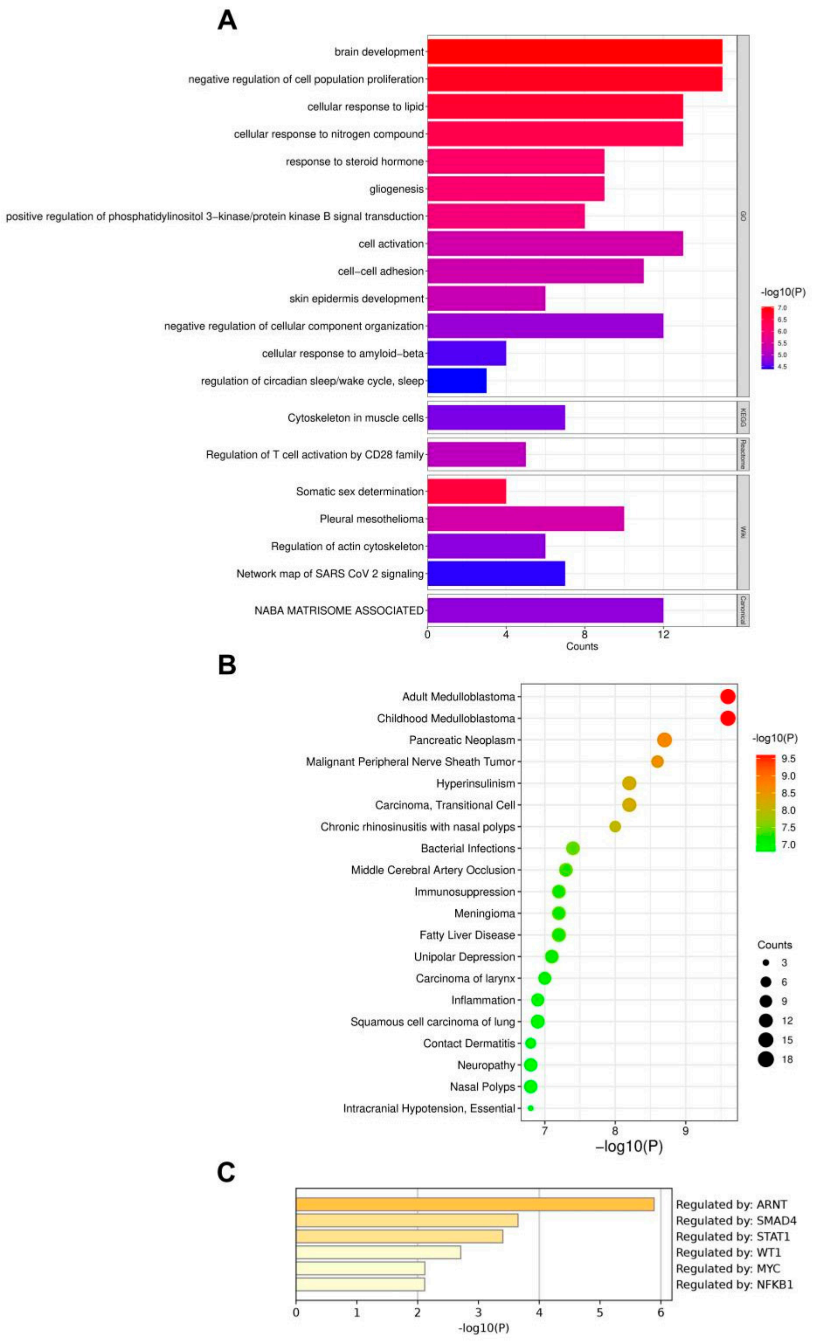

Figure S26 Functional, disease-associated and regulator enrichment analyses of the Fibroblast 6 subset. (A) Bar plot illustrating biological functional enrichment results for the Fibroblast 6 subset. (B) Dot plot depicting disease-related enrichment profiles of the Fibroblast 6 subset. (C) Bar plot displaying enrichment of transcriptional regulators associated with the Fibroblast 6 subset.

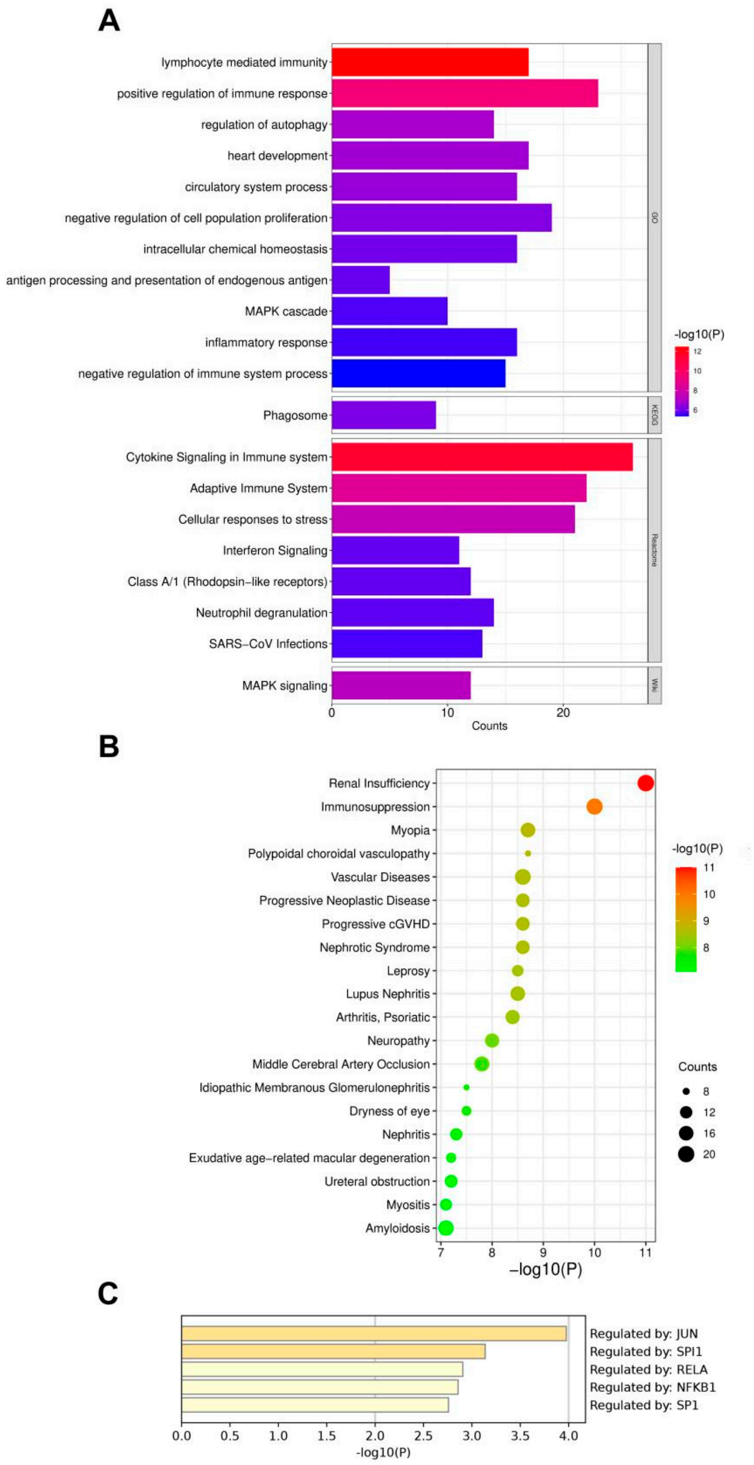

Figure S27 Functional, disease-associated and regulator enrichment analyses of the Fibroblast 7 subset. (A) Bar plot illustrating biological functional enrichment results for the Fibroblast 7 subset. (B) Dot plot depicting disease-related enrichment profiles of the Fibroblast 7 subset. (C) Bar plot displaying enrichment of transcriptional regulators associated with the Fibroblast 7 subset.

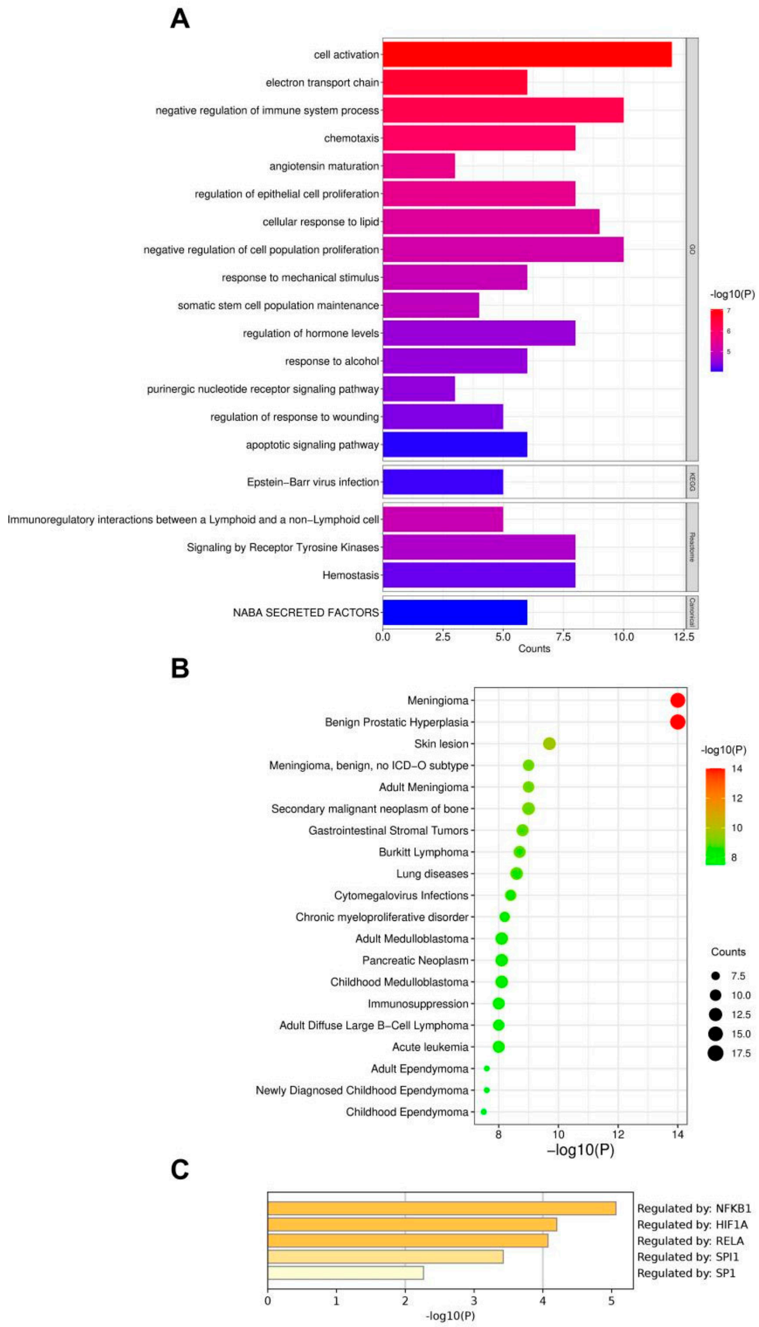

Figure S28 Functional, disease-associated and regulator enrichment analyses of the Fibroblast 8 subset. (A) Bar plot illustrating biological functional enrichment results for the Fibroblast 8 subset. (B) Dot plot depicting disease-related enrichment profiles of the Fibroblast 8 subset. (C) Bar plot displaying enrichment of transcriptional regulators associated with the Fibroblast 8 subset.

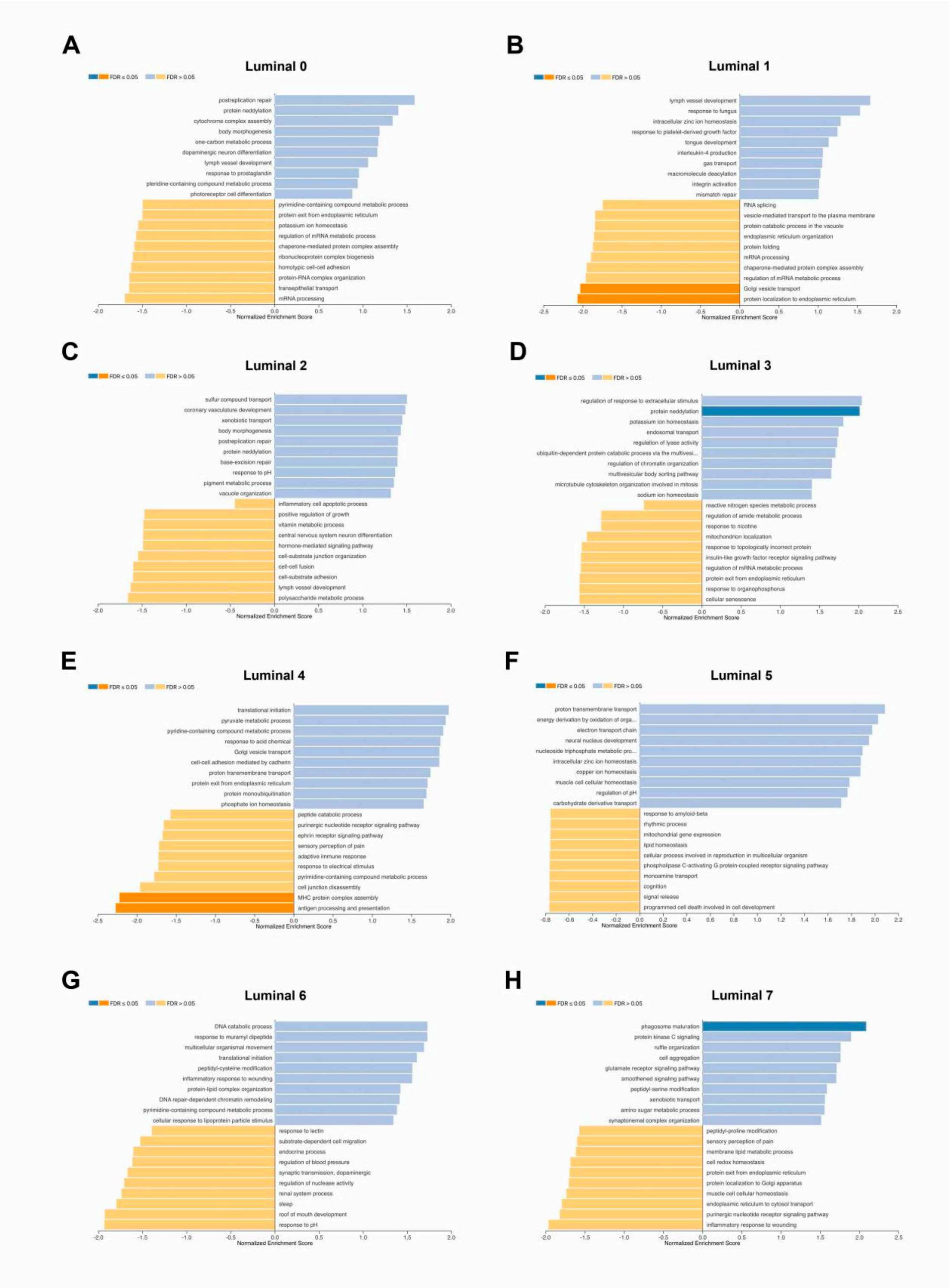

Figure S29 GSEA displaying FDR-adjusted significant enrichment of GO gene sets in eight luminal subsets: Luminal 0 (A), the Luminal 1 (B), the Luminal 2 (C), the Luminal 3 (D), the Luminal 4 (E), the Luminal 5 (F), the Luminal 6 (G), the Luminal 7 (H).

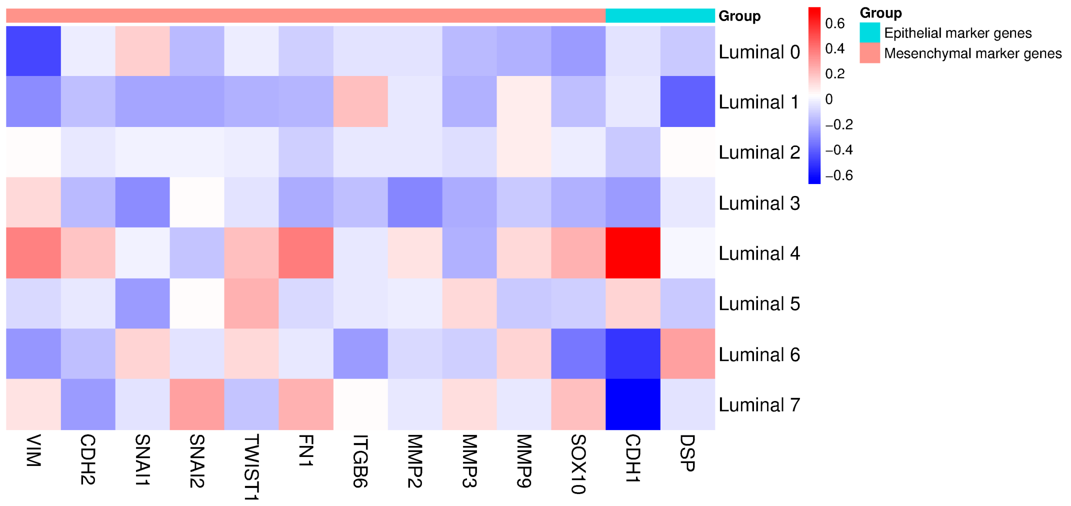

Figure S30 Heatmap illustrating the expression gradients of EMT-associated markers across eight Luminal subsets. FOXC2, GCS and OCLN were filtered out as they exhibited no detectable transcriptional signals across all tested subsets. The color gradient represented relative expression changes: red corresponds to upregulation and blue corresponds to downregulation. Top-side annotations categorized these marker genes into mesenchymal and epithelial clusters.

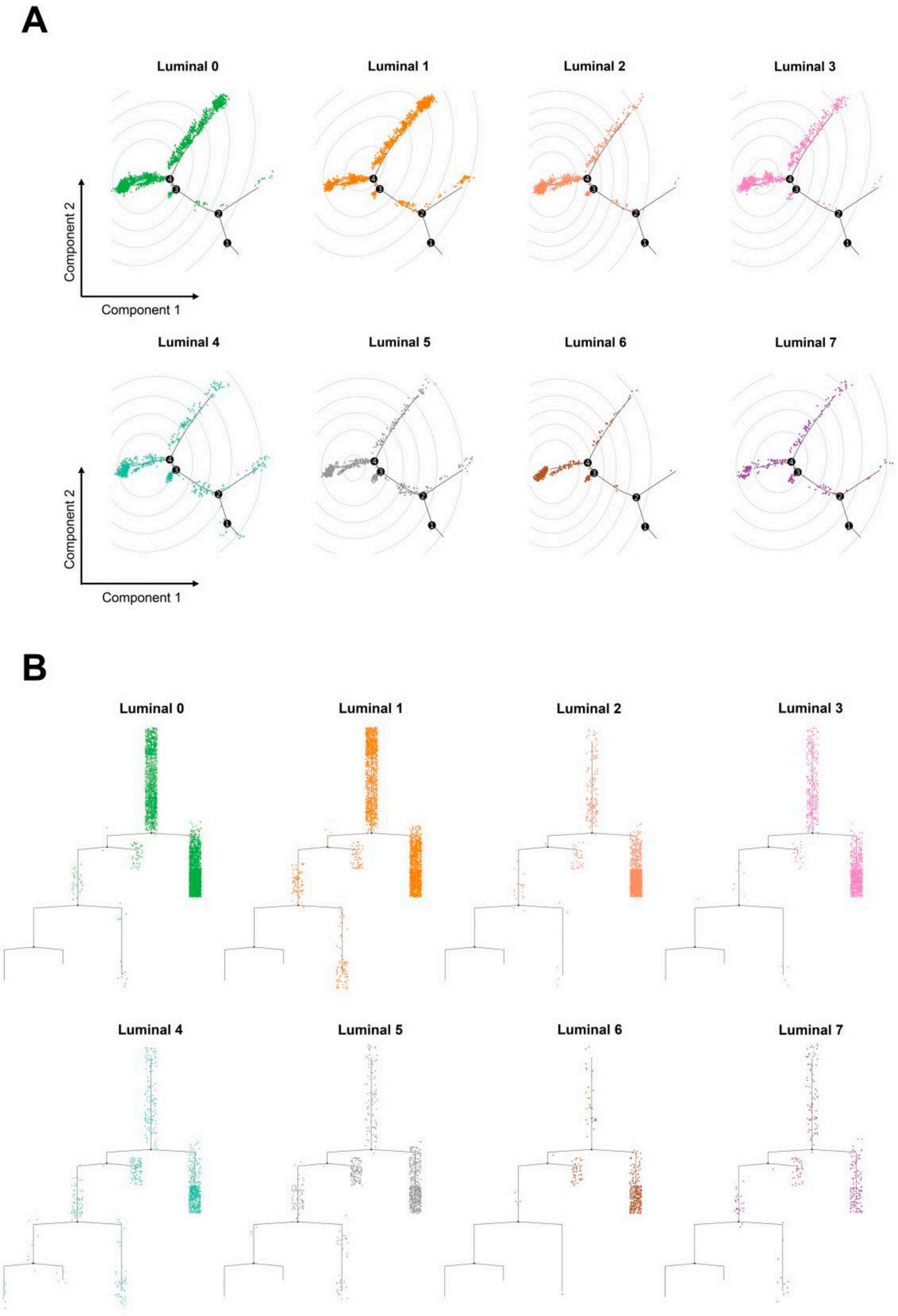

Figure S31 Pseudotime trajectory analysis of eight Luminal subsets. (A) Two-dimensional pseudotime plots showing differentiation trajectories for Luminal 0–7; colors distinguish individual Luminal subsets. (B) Tree-structured pseudotime layouts displaying the branched differentiation progression of the eight Luminal subsets with subset-specific coloring.

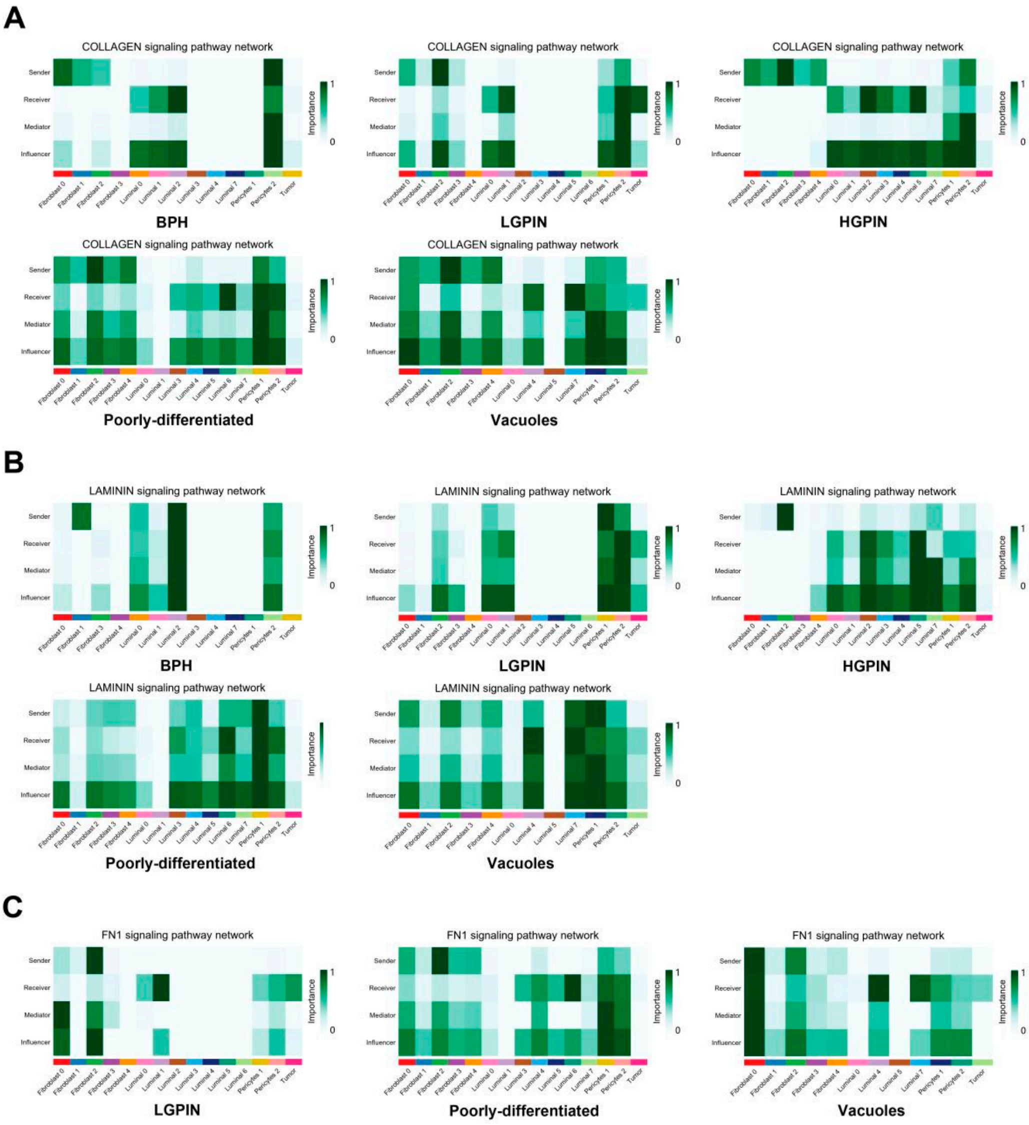

Figure S32 Heatmap based on four network centrality measures of three signaling pathways,showing the relative importance of Luminal, Fibroblast, pericyte and tumor subsets as sender, receiver, mediator and influencer in five pathological microregions. (A) COLLAGEN signaling pathway network, (B) LAMININ signaling pathway network, (C) FN1 signaling pathway network.

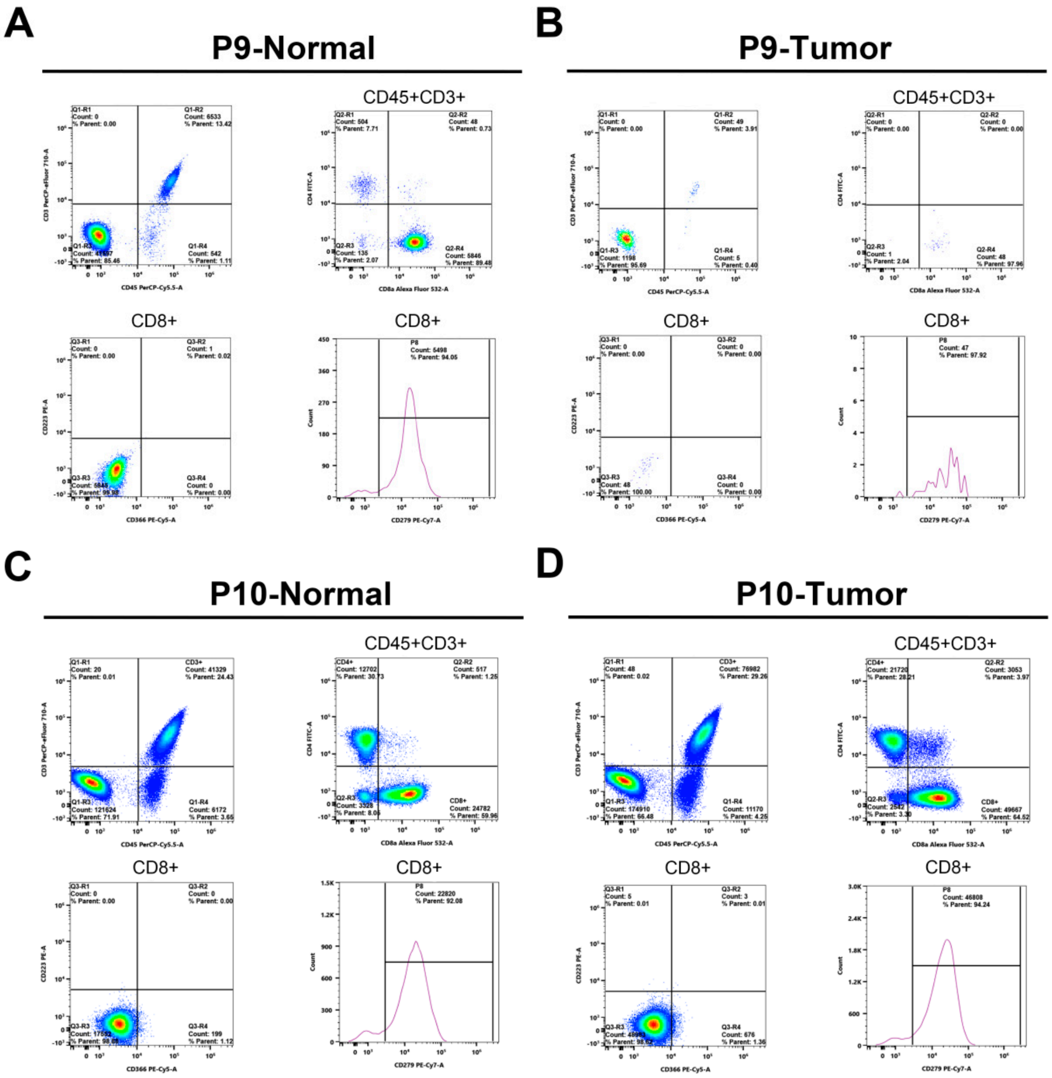

Figure S33 Flow cytometry comparison of T-cell abundance and exhaustion status between paired normal and PCa tissues from Patient. T-cell proportions and exhaustion profiles in the normal (A) and PCa (B) tissues harvested from Patient 9. T-cell proportions and exhaustion profiles in the normal (C) and PCa (D) tissues harvested from Patient 10.

Supporting Tables

Table S1 Summarizes basic clinical and pathological data for all 10 enrolled PCa patients. GS stands for Gleason Score. All patients received no prior androgen deprivation or other anti-tumor endocrine therapy before tissue collection.

Patient number	Age	Bone metastasis	Pathological diagnosis	GS (biopsy)	Endocrine therapy
Patient 1	68	No	Prostate acinar adenocarcinoma	[4+4]-[5+5]	None
Patient 2	76	Yes	Prostate acinar adenocarcinoma	[4+4]	None
Patient 3	72	No	Prostate acinar adenocarcinoma	[5+4]	None
Patient 4	55	Yes	Prostate acinar adenocarcinoma	[4+4]	None
Patient 5	72	No	Prostate acinar adenocarcinoma	[4+4]	None
Patient 6	66	No	Prostate acinar adenocarcinoma	[3+3]	None
Patient 7	69	No	Prostate acinar adenocarcinoma	[4+3]-[4+4]	None
Patient 8	58	No	Prostate acinar adenocarcinoma	[4+4]	None
Patient 9	42	No	Prostate acinar adenocarcinoma	[3+4]-[3+5]	None
Patient 10	66	No	Prostate acinar adenocarcinoma	[3+4]	None

Table S2 Detection status of single-cell spatial transcriptomic data.

Gene Panel Used	Human RNA 6k Discovery
Number of Cells	154168
Number of FOV	385
Total Transcripts Detected	133395041
Mean Transcripts per cell	865.26
Mean Features per cell	587.3
Mean Transcripts per FOV	346480.63
Total Negative Probs Detected	490632
Mean Negative Probs per cell	3.18
Total Falsecode Detected	166236
Mean Falsecode per cell	1.08
% Counts on Targeted Genes	99.51
% Cells Passed QC	88.88
